# Contrastive learning with mutual information enhancement and negative sample augmentation combined with KAN for text clustering

**DOI:** 10.1371/journal.pone.0354718

**Published:** 2026-07-30

**Authors:** Yuanmin Zhang, Hao Li, Chunzhi Xie, Yong Huang, Zhenyi Wu, Yanjun Li

**Affiliations:** 1 China Unicom (Sichuan) Industrial Internet Co. Ltd., Chengdu, Sichuan, People’s Republic of China; 2 School of Computer and Software Engineering, Xihua University, Chengdu, Sichuan, People’s Republic of China; 3 Sichuan Provincial Public Security Department, Science and Technology Information Center, Chengdu, Sichuan, People’s Republic of China; 4 School of Automation Engineering, University of Electronic Science and Technology of China, Chengdu, Sichuan, People’s Republic of China; Beijing University of Posts and Telecommunications, CHINA

## Abstract

This paper proposes a text clustering model based on mutual information-enhanced contrastive learning combined with Kolmogorov-Arnold Networks (KAN) to address challenges in text clustering, including insufficient robustness of text representations, feature redundancy, and the curse of dimensionality. Text clustering is essential for organizing unstructured textual data, yet existing methods often suffer from weak feature representations and limited scalability in high-dimensional spaces. The proposed model jointly addresses these three core issues by maximizing mutual information to capture nonlinear relationships among positive samples, expanding negative samples to enhance discriminability, and employing the KAN architecture to adapt to the complex structures of high-dimensional data. Experimental results on eight benchmark datasets demonstrate that the proposed model achieves state-of-the-art accuracy on seven out of eight datasets and leads normalized mutual information on six datasets, outperforming existing text clustering baselines. To validate its practical utility, the model was applied to cluster Weibo posts collected via web crawlers using “technology” as the keyword during January and February 2025. The case study results reveal that the model effectively identifies meaningful thematic clusters—such as AI applications, technology industry trends, and consumer electronics discussions—confirming its applicability to real-world social media data.

## 1 Introduction

Text clustering is a fundamental task in the field of Natural Language Processing (NLP), which aims to automatically partition an unlabeled collection of texts into several clusters characterized by internal semantic consistency and mutual semantic distinctiveness based on their semantic content. This technique serves as a critical enabler for efficient organization, categorization, and summarization of textual information [1,2], playing a vital role in applications such as information retrieval and content recommendation.

In recent years, deep neural network-based text clustering methods have gradually become mainstream. Their core principle lies in synergistically optimizing clustering objectives with deep representation learning, leading to significant improvements in clustering performance. In this evolutionary process, contrastive learning, as an effective paradigm for representation learning, has played a crucial role. Research exemplified by contrastive learning-based text embedding methods [3] learns discriminative feature representations by pulling semantically consistent positive pairs closer and pushing semantically distinct negative pairs farther apart in a high-dimensional space, thereby providing high-quality semantic inputs for subsequent clustering tasks. This approach can effectively mitigate the common anisotropy issue [4] in pre-trained language models—where the learned vector representations of words or sentences are unevenly distributed in space and exhibit directional biases—thus learning a more uniform and isotropic semantic space, ultimately enhancing the robustness and generalization ability of text representations.

Building upon this, Zhang et al. were the first to introduce contrastive learning into text clustering tasks, proposing the Sentence-level Contrastive Clustering (SCCL) [5] model. The SCCL model obtains relatively discriminative text representations by constructing positive sample pairs and optimizing a contrastive loss. However, the complete absence of supervised signals also leads to insufficient discriminative power in fine-grained semantics for the representations it generates. Subsequently, Zheng et al. identified this issue in their Robust Self-supervised Text Clustering (RSTC) [6] model, which introduces a self-supervised mechanism to generate pseudo-labels, thereby promoting intra-cluster compactness and inter-cluster separability, ultimately achieving more discriminative text representations. Nevertheless, existing contrastive learning-based text clustering methods commonly confine the alignment of positive sample pairs to linear operations within Euclidean space, failing to fully exploit and leverage the complex non-linear relationships between sample pairs.

Another line of research based on instance discrimination attempts to improve clustering performance by modeling correlations among instances. For instance, Chang et al. [7] transformed the clustering task into a binary classification problem. However, these studies primarily focus on instance-level correlations, which can easily lead to feature redundancy—a phenomenon where different learned feature dimensions become highly correlated, carrying substantial redundant information and reducing the efficiency of the representation. Although Tao et al. [8] attempted to circumvent this redundancy by adjusting the information correlation among instance-level features, their method remains confined to the instance level, treating text features as an inseparable whole without delving into fine-grained disentanglement at the feature dimension level. Therefore, how to enhance semantic information between positive samples while effectively removing redundancy arising from instance correlations at the feature level remains a critical challenge for current text clustering methods.

The rapid advancement of deep learning has continuously driven innovation in text embedding techniques; however, the inherent curse of dimensionality in high-dimensional space persists. Although existing embedding techniques can convert texts into low-dimensional vector representations, forming relatively compact data distributions to some extent, models still face challenges related to data sparsity when processing original high-dimensional sparse features. This phenomenon can render distance metrics ineffective for data points that should be proximate in high-dimensional space, causing their similarity to become insignificant and consequently affecting the final clustering quality.

While traditional dimensionality reduction techniques such as Principal Component Analysis [9] and feature selection methods can alleviate the sparsity issue in high-dimensional data to some degree, they possess limited capacity for modeling non-linear, complex high-dimensional data, making it difficult to capture the underlying structure of textual data. Consequently, the resulting text representations are suboptimal. Thus, how to more effectively address the curse of dimensionality in text clustering constitutes another pressing challenge that requires resolution.

To address the aforementioned challenges, this paper proposes a text clustering model based on mutual information enhancement and negative sample augmentation with Kolmogorov–Arnold Networks (KAN) [10]. Specifically, the main contributions and innovations of this study are as follows:

- *Addressing the issue of insufficient non-linear relationship modeling:* Inspired by the principle of mutual information maximization in information theory, the model captures deep non-linear dependencies between positive sample instances by maximizing their mutual information. This is combined with a negative sample augmentation-based contrastive learning strategy, compelling the model to learn more refined and discriminative text representations.
- *Addressing the issue of feature-level redundancy:* By minimizing mutual information between instances across all feature dimensions as a regularization mechanism, the model purposefully reduces redundancy among feature dimensions, forcing it to learn more independent and discriminative features. This resolves the limitation of existing methods that only address instance-level correlations.
- *Addressing the issue of the curse of dimensionality:* The model incorporates an effective KAN variant to replace traditional multi-layer perceptrons, enabling better handling of complex non-linear transformations in high-dimensional data. This helps the model adapt to and model intricate data structures in high-dimensional space, thereby mitigating the impact of data sparsity.

To systematically validate the effectiveness of the proposed method, this paper conducts extensive comparative experiments on eight public datasets. The experimental results demonstrate that the proposed model achieves optimal performance across key metrics such as accuracy.

The remainder of this paper is organized as follows: Section 2 reviews and analyzes related work; Section 3 elaborates on the specific design and implementation of the proposed model; Section 4 describes the experimental setup and presents the experimental analysis; Section 5 discusses key issues of this work, including the analysis of framework effectiveness and rationality, research implications, and a comparison between the proposed model and existing literature; Section 6 concludes the paper and discusses future research directions.

## 2 Related work

### 2.1 Traditional text clustering methods

Traditional text clustering research primarily employs algorithms based on partitioning, hierarchy, density, grid, fuzzy sets, and graphs [11]. The K-Means algorithm [12], a representative partitioning-based method, achieves data partitioning by iteratively optimizing intra-cluster distances. Hierarchical clustering encompasses bottom-up (agglomerative) and top-down (divisive) strategies, exemplified by Agglomerative Clustering [13] and Divisive Clustering [14], respectively. However, both partitioning and hierarchical methods require the number of clusters to be pre-specified, which presents limitations when processing text data with unknown structures. To address this limitation, density-based clustering methods, such as DBSCAN [15] and DENCLUE [16], dynamically identify clusters by analyzing the distribution density of data points. Grid-based clustering algorithms reduce computational complexity by partitioning the space into finite units, with typical methods including STING, CLIQUE [17], and Wave-Cluster [18]. Fuzzy clustering, based on fuzzy set theory, introduces a membership function, overcoming the limitation of binary sample attribution in traditional clustering, with fuzzy C-Means [19] as its representative. Graph clustering models the global relationships of data through graph structures, circumventing sensitivity to initialization, with typical algorithms including Normalized Cut (Ncut) [20] and Spectral Clustering [21]. However, these traditional methods generally assume that the input data has been modeled into well-defined feature representations. Text data, conversely, possesses complex semantic structures and extremely high sparsity, making it difficult to effectively model semantic similarity using traditional metrics like Euclidean distance. This fundamental limitation has motivated the emergence of deep learning-based text clustering methods.

### 2.2 Deep learning text clustering methods

The advancement of deep neural networks has created new opportunities for text clustering. Xu et al. [22] first introduced Convolutional Neural Networks (CNNs) to text clustering, improving sentence representations by extracting local features. However, CNNs are limited by their receptive field, making it difficult to capture contextual dependencies in long text sequences. To address this, Fan et al. [23] combined CNNs with Bidirectional Long Short-Term Memory networks (Bi-LSTM), encoding the surrounding semantic context into vector representations to capture deeper semantic dependencies, effectively mitigating the limitations of CNNs in processing long texts. While CNN-based methods excel at local feature extraction, they are less effective at modeling long-range dependencies, with semantic information potentially degrading as network depth increases. Conversely, Recurrent Neural Network (RNN)-based methods can effectively capture sequential information but suffer from lower training efficiency.

In contrast, pre-trained language models based on the Transformer architecture, through their self-attention mechanism, can effectively capture global contextual information, demonstrating significantly superior capability in modeling textual semantic structures compared to the former two approaches. Consequently, recent research has predominantly adopted Transformer-based models for text encoding. In this direction, Zhang et al. [5] proposed SCCL, which first integrated contrastive learning with text clustering tasks. It employs a Pre-trained Sentence Bidirectional Encoder Representations from Transformers (SBERT) for encoding while jointly optimizing representation learning and clustering objectives. However, this model does not sufficiently address the clustering degeneration problem, where models tend to map all samples into a compact region in the representation space. Subsequently, RSTC addressed this issue by innovatively using optimal transport to generate pseudo-labels for self-supervised training, effectively enhancing intra-cluster compactness and inter-cluster separability. However, RSTC inadequately addresses the issue of feature redundancy in text representations—where different feature dimensions are highly correlated and carry substantial repetitive information—which can limit the discriminative power of clustering. To address this deficiency, Reliable Pseudo-labeling via Optimal Transport with Attention for Short Text Clustering (POTA) [24] employs an instance-level attention mechanism to capture semantic relationships between samples and incorporates this as a regularization term within the optimal transport optimization. However, POTA does not sufficiently account for the information content within sentences. Mutual Information Maximization for Short Text Clustering (MIST) [25] addresses this by maximizing mutual information between sentences and tokens at the token level, enabling the model to capture both inter-sample semantic relationships and fine-grained intra-sample information. Recent studies by Ortakci [26] and Ortakci & Borhan [27] further explored optimization methods for long text clustering based on SBERT. By employing sentence-level and chunk-level embedding generation strategies, they effectively addressed the truncation issue of SBERT when processing overly long texts, offering new approaches for applying text clustering in real-world scenarios.

### 2.3 Contrastive learning text clustering methods

Inspired by the success of contrastive learning in the computer vision field [28], researchers have applied it to NLP, effectively mitigating the anisotropy problem in pre-trained language models—a phenomenon where the vector representations of words or sentences are unevenly distributed in the embedding space and confined to a narrow conical region [4]. The core principle of contrastive learning is to learn discriminative feature representations by pulling semantically consistent positive samples closer and pushing semantically distinct negative samples apart in a high-dimensional space. This section systematically reviews existing work from two dimensions: positive sample augmentation and negative sample augmentation.

#### 2.3.1 Positive sample augmentation strategies.

Positive sample augmentation strategies aim to construct different views for an anchor sample with the same semantics. Based on the augmentation granularity, these can be categorized into token-level, word-level, and dropout augmentation. Token-level strategies obtain augmented samples by transforming the embedding representation at the tokenization layer. ConSERT [29] proposed four data augmentation methods: adversarial attacks (adding worst-case perturbations), random shuffling of token order, random token deletion, and token dropout. CARDS [30] constructs positive sample pairs by combining augmentation with retrieved data. However, although token-level augmentation is effective, it carries the risk of damaging the original semantics—excessive token transformation may cause the augmented sample to deviate from the original meaning. Word-level strategies generate positive samples by replacing words in a sentence. CLINE [31] uses WordNet to generate positive and negative sample pairs by replacing words with synonyms and antonyms, respectively. CERT [32] captures global semantic features through context-aware word replacement. CLEAR [33] comprehensively adopts multiple strategies, including random deletion, span deletion, span swapping, and synonym replacement. The limitation of such methods is that word-level substitution may alter the original meaning of the sentence or introduce semantic bias. In contrast, dropout augmentation’s core advantage lies in preserving the original semantics and structure of the input text. This method generates positive sample pairs by applying different dropout masks [34] to the same input, with the most representative example being Simple Contrastive Learning of Sentence Embeddings (SimCSE) [35]. The subsequent USCAL [36] model builds upon SimCSE by utilizing gradients from the dropout contrastive loss to generate adversarial examples as additional positive samples, further enriching the diversity of positive samples.

#### 2.3.2 Negative sample augmentation strategies.

The quality of negative samples is equally crucial for contrastive learning, especially hard negatives—samples that are semantically distinct from the anchor but difficult for the model to distinguish due to high similarity in their representations. In NLP, obtaining hard negatives primarily relies on supervised label information or algorithm-assisted generation [37]. SimCSE and Disco [38] utilize contradiction sentences from Natural Language Inference (NLI) datasets as hard negatives. PairSupCon [39] also employs supervised NLI data but uses importance sampling to select higher-weight samples as hard negatives. However, these methods depend on annotated data, limiting their applicability in unsupervised scenarios. For unsupervised hard negative construction, AdCSE [37], inspired by AdCo [40] in computer vision, utilizes adversarial training to dynamically generate hard negatives within the MoCo framework, resulting in high quality but significant computational cost. MixCSE [41] proposes an efficient alternative by constructing hard negatives through mixing the features of positive samples and random negatives, balancing discriminability and computational efficiency while significantly enhancing the gradient signal strength in contrastive learning. Besides hard negatives, false negatives—samples semantically identical to the anchor but incorrectly treated as negatives due to a lack of labels—are also a critical factor affecting contrastive learning performance. DCLR [42] systematically investigates this issue for the first time, employing a debiasing method to eliminate false negatives that might cause anisotropy. However, this method filters samples based on a Gaussian distribution assumption, potentially introducing misjudgments. SNCSE [43] proposes using the negated form of anchors, obtained through supervised methods, as soft negatives, effectively alleviating the feature suppression issue in contrastive learning—where models lose important semantic features by incorrectly pushing away semantically similar negative samples.

Overall, existing research on negative sample augmentation predominantly focuses on constructing hard negatives, with relatively limited exploration of handling false negatives. To address this gap, our previously proposed contrastive learning strategy IHSF [44], based on negative sample augmentation, enables more refined processing of both hard negatives and false negatives in high-dimensional space, thereby enabling deeper exploitation of the model’s sentence representation capability. A comprehensive analysis of representative works such as SCCL, RSTC, and MIST reveals three common issues in existing contrastive learning-based text clustering methods: (1) Contrastive learning of positive sample pairs is often confined to alignment operations in linear space, failing to fully exploit non-linear relationships between samples—a limitation systematically discussed in classic studies on applying contrastive learning to linear embedding scenarios [45]; (2) Insufficient attention is paid to feature-level redundancy, with existing methods primarily focusing on instance-level correlation modeling without delving into feature dimension decoupling; (3) When processing high-dimensional sparse data, these methods still suffer from the representational capacity bottleneck imposed by the curse of dimensionality. These deficiencies constitute the primary motivation for this study.

### 2.4 High-dimensional data processing and KAN networks

Processing high-dimensional data is a core challenge in text clustering. While traditional dimensionality reduction techniques like Principal Component Analysis [9] and feature selection methods can alleviate data sparsity to some extent, they have limited capacity for modeling the non-linear and complex structures inherent in high-dimensional data. A typical manifestation of the curse of dimensionality in high-dimensional space is the homogenization of distances between data points, leading to less distinct similarities between neighboring points and consequently degrading clustering quality.

KAN is an emerging neural network architecture grounded in the Kolmogorov-Arnold representation theorem. Unlike traditional Multi-Layer Perceptrons (MLPs), which use fixed activation functions on nodes, KAN employs learnable activation functions (often parameterized as spline functions) on the edges of the network. This allows it to adaptively fit complex non-linear transformations. This characteristic endows KAN with significant advantages in the following aspects [46]: (1) Complex Non-linear Relationship Modeling: The spline basis functions in KAN can more flexibly approximate non-linear manifolds in high-dimensional space; (2) Interpretability: By analyzing the learned spline functions, the contribution of each input dimension to the output can be intuitively understood; (3) Adaptability: KAN can dynamically adjust the representation space according to the data distribution, demonstrating better robustness for high-dimensional sparse data. KAN networks have shown potential in processing graph-structured data and high-dimensional tabular data. HyperKAN [47] introduces KAN into hypergraph representation learning, adjusting structural features based on similarity to effectively address issues of feature redundancy for high-degree nodes and insufficient information for low-degree nodes in hypergraphs. In the field of feature selection, KAN-based selectors have proven effective in identifying non-linear feature interactions and eliminating redundant dimensions, achieving excellent performance in high-dimensional multi-classification tasks [48].

Based on the above analysis, integrating KAN into text clustering frameworks holds substantial theoretical justification and innovative value. First, text data possesses complex non-linear semantic structures, which KAN’s spline activation functions can model more effectively. Second, the prevalent issue of feature redundancy in text representations can be alleviated through KAN’s dynamic adaptability. Finally, KAN’s strengths in handling high-dimensional sparse data are expected to mitigate the negative impact of the curse of dimensionality on clustering performance. This study combines KAN with mutual information-enhanced contrastive learning, aiming to address the two core challenges of non-linear modeling and redundancy reduction at the feature level.

## 3 Proposed model

The text clustering model proposed in this paper integrates contrastive learning based on mutual information enhancement and negative sample augmentation with a KAN network, hereinafter referred to as the Mutual Information‑based KAN Network Model (MIKAN). It addresses three key issues: (1) How to augment the mutual information between positive instance pairs to enhance contrastive learning, thereby improving the robustness of text representations learned by the model. (2) How to mitigate feature-level redundancy within instance representations to bolster the model’s discriminative capability for individual instances. (3) How to mitigate the curse of dimensionality during the clustering process to optimize the model’s clustering performance. The solution for the first issue can be outlined as follows: An original text corpus is subjected to data augmentation to yield augmented text instance sets. These augmented sets are then encoded using a pre-trained encoder to produce corresponding augmented instance embedding matrices. Subsequently, the information content of these augmented instance sets is maximized before being fed into an incorporated negative sample augmentation strategy [44] to continue contrastive learning training. Consequently, by maximizing the mutual information between positive instance pairs, this strategy enables contrastive learning to effectively capture the nonlinear relationships among positive samples, thereby achieving more robust text representations. The solution for the second issue can be outlined as follows: After encoding the raw text through an encoder, vector representations of text instances are obtained. At this stage, feature-level mutual information among these text instance representations is computed, and its redundancy is minimized at the feature level. This strategy further enhances the model’s ability to learn more discriminative representations for instances. The solution for the third issue involves the novel integration of KAN within the clustering network. The core innovation of KAN lies in replacing the fixed activation functions of traditional MLPs with learnable spline activation functions. Research indicates that this design endows KAN with distinct advantages in high-dimensional data modeling. First, its spline basis functions enable more flexible approximation of nonlinear manifolds in high-dimensional spaces. Studies on hyperspectral image classification have demonstrated that KAN can “accurately capture complex nonlinear relationships” and “effectively mitigate the curse of dimensionality” [46,49]. Second, KAN often requires fewer learnable parameters while maintaining high prediction accuracy, exhibiting superior parameter efficiency [50]. It should be noted, however, that whether KAN can fundamentally overcome the curse of dimensionality remains a subject of ongoing debate within the academic community. Nevertheless, recent research at least suggests its potential in terms of adaptability to high-dimensional data. Therefore, feeding the feature representations obtained through contrastive learning, along with the high-dimensional data structures after feature redundancy reduction, into the KAN network is expected to alleviate the curse of dimensionality to a certain extent.

### 3.1 MIKAN

**Fig 1:**
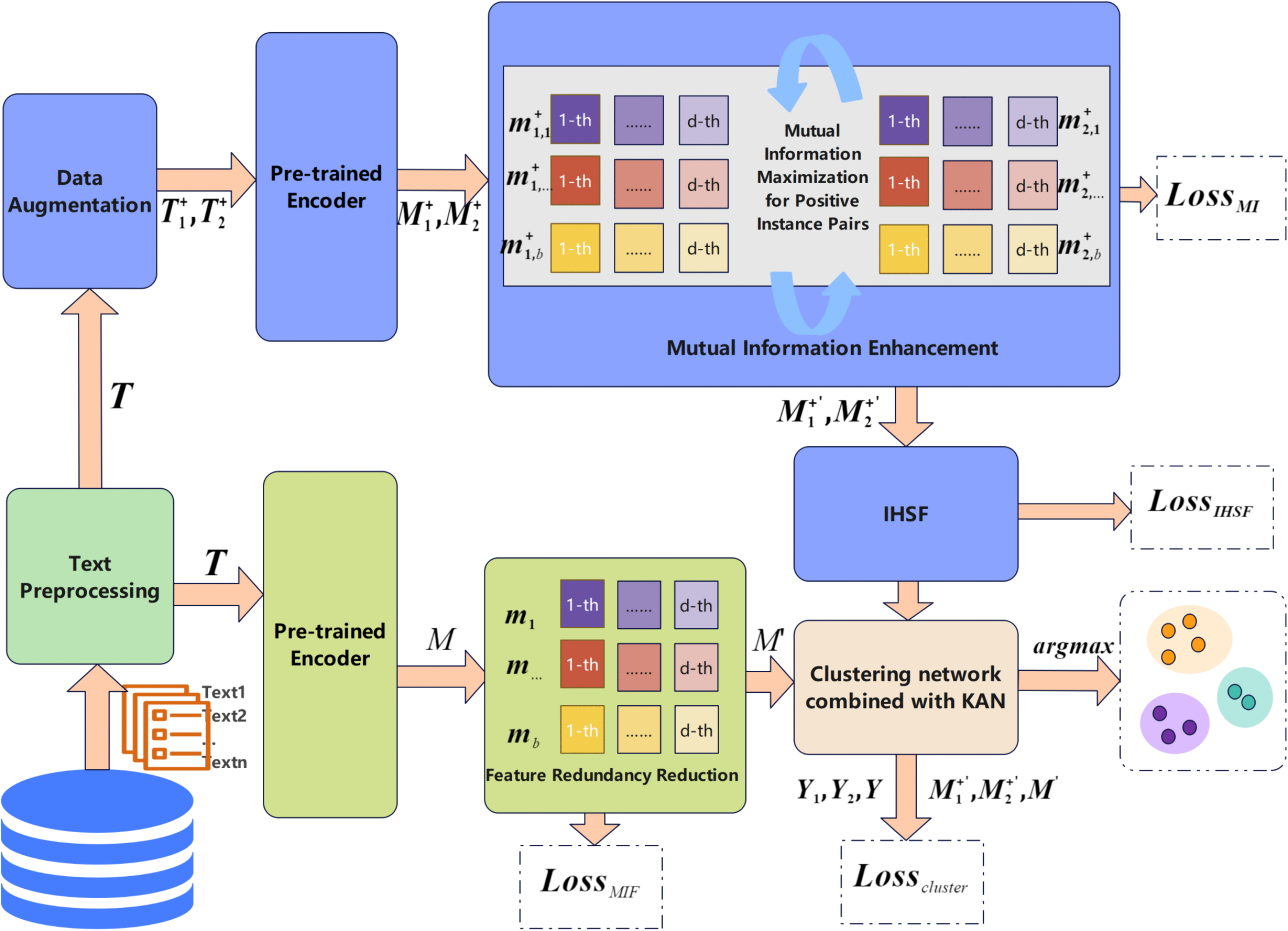
The relationship graph between nodes and their neighbors in social networks.

First, the raw text in the database is subjected to text preprocessing to obtain the original instance set *T*. Subsequently, the original instance set *T* is processed by a Pre-trained encoder to obtain the encoding matrix *M*, and feature redundancy reduction based on mutual information is performed to obtain the feature matrix $M^{\prime }$. Meanwhile, the original instance set *T* is fed into the positive sample instance information maximization module to obtain $\left(M_{1}^{+\prime },M_{2}^{+\prime }\right)$, and the augmented instances $M_{1}^{+\prime }$ and $M_{2}^{+\prime }$ are further enhanced via the negative sample augmentation-based contrastive learning strategy (IHSF) module to improve feature representation. Next, the feature-enhanced $M_{1}^{+\prime }$ and $M_{2}^{+\prime }$, together with $M^{\prime }$, are input into the KAN-based clustering network to obtain the corresponding label sets *Y*_1_, *Y*_2_, and *Y*. Finally, the network parameters are updated by computing the clustering loss Loss_cluster_ to obtain the final clustering results (as illustrated in Fig 1).

### 3.2 Mutual information maximization for positive instance pairs

Given that contrastive learning has extensively leveraged the similarity between text instances [35], mutual information theory is applied by the proposed model, building upon this foundation, to further maximize the information content of positive instance pairs. Initially, key concepts related to mutual information within information theory are introduced. Entropy *H* (Entropy), a fundamental concept in information theory, is utilized to quantify the degree of uncertainty within a system, thereby reflecting the randomness and distributional characteristics of the data. When the entropy value increases, greater system complexity and uncertainty are indicated; conversely, a decrease in entropy signifies enhanced system determinism. The entropy of a discrete random variable *X* can be expressed as follows:


$$\begin{array}{c} H(X)=-\sum_{x}p(x)\log p(x), \end{array}$$ (1)

where *p*(*x*) denotes the probability of the random variable *X* taking on the value *x*. Conditional entropy is defined between two random variables. It can be employed to quantify the amount of information of one random variable *Y*, given that another random variable *X* has taken a specific value *x*. The conditional entropy is computed as the expected value of the conditional entropy *H*(*Y*|*X* = *x*) with respect to *X*, and can be expressed as:


$$\begin{array}{c} \;H(Y|X)=-\sum_{x,y}p(x,y)\log\frac{p(y|x)}{p(x)}, \end{array}$$ (2)

where *p*(*x*,*y*) represents the joint probability of *X* and *Y*, and $p(x)=\sum_{y\in Y}p(x,y)$ is the corresponding marginal probability distribution. Mutual information, on the other hand, quantifies the degree of dependence between two random variables. It indicates how much additional information one feature provides about another. By combining entropy and conditional entropy, the mutual information utilized by the proposed model can be computed in the following form:


$$\begin{array}{c} I(X,Y)=\sum_{x,y}p(x,y)\log\frac{p(x,y)}{p(x)p(y)}, \end{array}$$ (3)

where $p(x)=\sum_{y}p(x,y)$ and $p(y)=\sum_{x}p(x,y)$ represent the marginal probability distributions of *X* and *Y*, respectively.

As illustrated in the positive sample pair mutual information maximization module in Fig 1, the instance set *T* undergoes data augmentation (using the same set of data augmentation strategies but applying two independent and random transformations) to obtain two augmented text instance sets: $T_{1}^{+}=\{t_{1,1}^{+},t_{1,2}^{+},...,t_{1,b}^{+}\}$ and $T_{2}^{+}=\{t_{2,1}^{+},t_{2,2}^{+},...,t_{2,b}^{+}\}$, where *b* denotes the batch. Subsequently, the augmented text instance sets $T_{1}^{+}$ and $T_{2}^{+}$ are encoded by a Pre-trained encoder (e.g., BERT, RoBERTa [66]) to obtain the feature representation matrices corresponding to the positive sample instance sets, denoted as $M_{1}^{+}$ and $M_{2}^{+}$, respectively, where $M_{1}^{+}=[m_{1,1}^{+},m_{1,2}^{+},...,m_{1,b}^{+}]$ and $M_{2}^{+}=[m_{2,1}^{+},m_{2,2}^{+},...,m_{2,b}^{+}]$. Here, each $m_{x,y}^{+}=[m_{x,y,1},m_{x,y,2},...,m_{x,y,d}]$ represents the *d* -dimensional feature vector obtained by encoding the augmented text instance *y*, where *d* is the dimension of the encoded representation produced by the encoder. Consequently, $M_{1}^{+},M_{2}^{+}\in {\mathbb{R}}^{b\times d}$.

Since the objective of this module is to maximize the information content of positive instance pairs, we first compute their joint probability distribution based on mutual information theory. This module achieves this by performing a matrix multiplication between the transpose of the feature representation matrix $M_{1}^{+}$ and $M_{2}^{+}$, which inherently involves dot products and summation across the batch dimension. This process yields the joint probability distribution matrix *Q*, which is formulated as:


$$\begin{array}{c} Q_{ij}=\sum_{k=1}^{B}M_{1k}^{+}(M_{2k}^{+}{)}^{T}. \end{array}$$ (4)

The joint probabilities for the set of positive instance pairs form the joint probability distribution matrix *Q*, where $Q\in {\mathbb{R}}^{D\times D}$. Subsequently, a symmetric summation operation is applied to *Q* to ensure a more balanced calculation of the information content for positive sample instance pairs. On this basis, the feature matrix is normalized using *L*_2_ normalization—that is, each feature vector is divided by the square root of the sum of its squared components (Euclidean norm)—projecting it onto the unit hypersphere. This normalization approach is consistent with standard feature geometry and cosine similarity computation, enhancing training stability by constraining the numerical range of the feature vectors, while ensuring that positive sample instance pairs across different batch sizes yield reasonable numerical ranges when computing the joint probability distribution. At this point, the joint probability distribution $Q_{ij}^{\prime }$ for each pair of positive sample instance feature vectors can be obtained. The marginal probability distributions are then computed as shown in Equations (5) and (6):


$$\begin{array}{c} Q_{i}=\sum_{j=1}^{D}Q_{ij}^{\prime }, \end{array}$$ (5)

$$\begin{array}{c} Q_{j}=\sum_{i=1}^{D}Q_{ij}^{\prime }. \end{array}$$ (6)

Once the joint probability distribution $Q^{\prime }$ and the marginal probability distributions $Q_{i},Q_{j}$ for each pair of positive instance feature vectors are obtained, the proposed model computes the mutual information loss for positive instance pairs using Equation 7:


$$\begin{array}{c} Loss_{MII}=\sum_{i=1}^{D}\sum_{j=1}^{D}Q_{ij}^{\prime }\log\frac{Q_{ij}^{\prime }}{\left|\alpha Q_{i}Q_{j}\right|}. \end{array}$$ (7)

Here, α is a smoothing factor. The proposed model utilizes α to appropriately scale the marginal distributions, thereby preventing division-by-zero or negative values in the denominator during loss computation. Through the mutual information enhancement module, the feature representation matrices $M_{1}^{+}$ and $M_{2}^{+}$ undergo further information refinement, which improves the robustness of the text representations learned by the model. The information-enhanced feature representation matrices, $M_{1}^{+\prime }$ and $M_{2}^{+\prime }$, are then passed to the IHSF module. This module further refines the robustness of text representations by optimizing a contrastive loss, ultimately leading to superior performance in subsequent clustering tasks.

### 3.3 Feature-level redundancy reduction via mutual information

This section aims to suppress feature redundancy within instances by applying feature-level mutual information constraints, thereby enhancing the model’s ability to extract more discriminative features for each instance. The proposed model quantifies the degree of redundancy between features by analyzing the correlation among instance feature dimensions. Let the original text instance set be $T=\{t_{1},t_{2},...,t_{b}\}$, where *b* denotes the batch size of instances fed into the encoder. These instances are then encoded into a feature matrix $M\in {\mathbb{R}}^{b\times d}$ using a Pre-trained encoder. Here, each row vector $m_{i}\in {\mathbb{R}}^{d}$ represents the encoding of the *i*-th sample, specifically $m_{i}=(m_{i,1},m_{i,2},...,m_{i,d})$, and *d* is the dimensionality of the feature space. Furthermore, to ensure semantic consistency and alignment of encoded representations between original and augmented texts in the feature space, the encoder employed for the original text instances shares parameters with the encoder utilized for the augmented text instances. To reduce feature redundancy, the proposed model incorporates the mutual information estimated among features from the sample feature matrix *M* as a penalty term, and constrains feature independence by optimizing the objective function.

Previous studies [51] have demonstrated a strong correlation between the relatedness of feature dimensions and the magnitude of their joint probability distribution. Specifically, higher dimensional relatedness corresponds to a greater value in the joint probability distribution. Let $P(E_{i})$ and $P(E_{j})$ denote the marginal probability distributions for the random variables representing the *i*-th and *j*-th features, respectively, and $P(E_{i},E_{j})$ be their joint probability distribution. In the proposed model, $P(E_{i},E_{j})$ is derived through the following procedure: First, the feature vector matrix *M* is normalized to obtain $M^{norm}\in {\mathbb{R}}^{b\times d}$, where an element $M_{ij}^{norm}$ of the matrix $M^{norm}$ is given by:


$$\begin{array}{c} M_{ij}^{norm}=\frac{M_{ij}}{\sqrt{\sum_{l=1}^{d}M_{il}^{2}}}. \end{array}$$ (8)

This normalization projects each feature vector onto a unit hypersphere, thereby mitigating the influence of feature magnitude differences on subsequent similarity computations. Subsequently, the self-correlation matrix $M^{self}\in {\mathbb{R}}^{d\times d}$ is obtained by multiplying the transpose of $M^{norm}$, i.e., $M^{norm^{T}}$, with $M^{norm}$ itself, followed by a global normalization. Each element $M_{ij}^{self}$ of this self-correlation matrix can be expressed as:


$$\begin{array}{c} M_{ij}^{self}=\frac{\sum_{k=1}^{b}M_{ki}^{norm}M_{kj}^{norm}}{\sum_{p=1}^{d}\sum_{q=1}^{d}\left(\sum_{k^{\prime }=1}^{b}M_{k^{\prime }p}^{norm}M_{k^{\prime }q}^{norm}\right)}. \end{array}$$ (9)

Here, *k* and $k^{\prime }$ iterate over the batch dimension (from 1 to *b*), while *i*, *j*, *p*, *q* iterate over the feature dimensions (from 1 to *d*). Consequently, $M_{ij}^{self}$ quantifies the dependency between the *i* -th and *j* -th features. A larger value of $M_{ij}^{self}$ indicates higher shared information and more pronounced redundancy between the two dimensions, and vice versa. To provide a probabilistic interpretation of feature-level redundancy, we approximate the joint probability distribution between feature dimensions using the normalized self-correlation matrix. Specifically, we define $P(E_{i},E_{j})=M_{ij}^{self}$, where $E_{i}$ and $E_{j}$ denote the *i* -th and *j* -th feature dimensions, respectively. It is important to note that this formulation treats $M^{self}$ as an empirical approximation of the joint probability, rather than a rigorously defined probability distribution that strictly satisfies the axioms of probability theory (e.g., all elements summing to exactly 1). This approximation provides a practical basis for quantifying feature dependencies within a batch. Based on this empirical joint distribution, the marginal probability distributions $P(E_{i})$ and $P(E_{j})$ can be obtained by summing over all possible values of the other variable:


$$\begin{array}{c} P(E_{i})=\sum_{j=1}^{d}P(E_{i},E_{j})=\sum_{j=1}^{d}M_{ij}^{self}, \end{array}$$ (10)

$$\begin{array}{c} P(E_{j})=\sum_{i=1}^{d}P(E_{i},E_{j})=\sum_{i=1}^{d}M_{ij}^{self}. \end{array}$$ (11)

Here, $P(E_{i})$ represents the empirical marginal probability of the *i* -th feature dimension, obtained by aggregating its dependencies with all other feature dimensions across the batch. Thus, after obtaining the joint and marginal probability distributions for each feature dimension, the feature-level mutual information (MI) regularization loss can be formulated as shown in Equation 12:


$$\begin{array}{c} Loss_{MIF}=\frac{1}{d^{2}}\sum_{i=1}^{d}\sum_{j=1}^{d}P(E_{i},E_{j})\log\frac{P(E_{i},E_{j})}{\left|\beta |P(E_{i})P(E_{j}\right)}, \end{array}$$ (12)

where β serves as a balancing hyperparameter in the proposed model. It is employed to appropriately relax the marginal probability distributions, thereby providing enhanced flexibility during the computation of the feature-level loss. In this manner, the proposed model incorporates the mutual information estimated among features from the sample feature matrix *M* as a penalty term, thereby achieving feature-level redundancy reduction for the text instance feature matrix *M* from an information-theoretic perspective. Consequently, the discriminative capability of the proposed model for text representations is enhanced.

### 3.4 Negative sample enhanced contrastive learning strategy

The feature representation matrices $M_{1}^{+\prime }$ and $M_{2}^{+\prime }$, which are derived from maximal information enhancement, are subsequently fed into the IHSF module. Within this module, a negative sample-enhanced contrastive learning strategy, originally proposed in our prior work [44], is leveraged to facilitate more effective feature learning. This strategy is specifically designed to increase the repulsion force for hard negative samples while concurrently enhancing the attraction to false negative samples. False negative samples refer to positive pairs that are mistakenly treated as negative pairs during contrastive learning, which may hinder the model from learning semantically consistent representations. Let *B* denote the total number of samples within the inputted matrix. Within this set, *M* (1⩽M⩽B−1) represents the number of hard negative samples, and *L* (1⩽L⩽N−1−M) represents the number of false negative samples. Consequently, the number of regular (or ’easy’) negative samples is T=N−1−M−L. Then, the *m*-th hard negative sample is denoted as $z_{H_{m}}$ (m=1,...,M); the *l*-th false negative sample as $z_{F,l}$ (l=1,...,L); and the *j*-th regular negative sample as $z_{j}$ (j=1,...,T).

The hard negative sample enhancement strategy is designed to enhance contrast by leveraging the repulsion forces extracted from hard negative samples. Upon identifying hard negative samples for each anchor, these are no longer treated as conventional negative samples. Instead, an amplified separation force is applied to push them further from the anchor in the high-dimensional semantic space. The repulsion force between the anchor sample *z*_Anchor_ and the hard negative sample $z_{H_{m}}$ is expressed as:


$$\begin{array}{c} \alpha_{Anchor,z_{H_{m}}}^{-}=1+\frac{\exp\left(\text{sim}(z_{Anchor},z_{H_{m}})/\tau \right)}{\sum_{j=1}^{T}\exp\left(G/\tau \right)}, \end{array}$$ (13)

where sim(·) denotes a similarity function. Consequently, the loss associated with hard negative samples can be defined as:


$$\begin{array}{c} {ℒ}_{H}=-\log\frac{\exp(\text{sim}(z_{\text{Anchor}},z_{\text{Anchor}}^{+})/\tau )}{\sum_{j=1}^{N}\exp(\text{sim}(z_{\text{Anchor}},z_{j})/\tau )+\frac{1}{M}\sum_{m=1}^{M}\alpha_{\text{Anchor},z_{H_{m}}}^{-}\cdot \exp(\text{sim}(z_{\text{Anchor}},z_{H_{m}})/\tau )}, \end{array}$$ (14)

where $z_{Anchor}$ denotes the anchor sample, $z_{Anchor}^{+}$ is its positive sample, $z_{j}$ represents ordinary negative samples, and $z_{H_{m}}$ denotes hard negative samples. The dynamic weighting coefficient $\alpha_{Anchor,z_{H_{m}}}^{-}\in [1,2]$ is computed by Equation (13). The denominator of this loss function consists of two components that are summed element-wise: the ordinary negative term $\exp(\text{sim}(z_{Anchor},z_{j})/\tau )$, which pushes the anchor away from ordinary negatives, and the hard negative enhancement term $\frac{1}{M}\sum_{m=1}^{M}\alpha_{Anchor,z_{H_{m}}}^{-}\cdot \exp(\text{sim}(z_{Anchor},z_{H_{m}})/\tau )$, which imposes additional penalty by averaging the weighted similarities of all hard negatives. The dynamic weight $\alpha^{-}$ adaptively adjusts the repulsion strength based on the similarity between the anchor and each hard negative: higher similarity yields a larger $\alpha^{-}$, amplifying the contribution of the corresponding hard negative in the denominator and thus pushing it further away. This design enables the model to selectively strengthen the discrimination against semantically similar yet categorically distinct negative samples, thereby improving the discriminative capacity of the learned representations.

False negative samples are characterized by their semantic similarity to anchor samples, despite being erroneously classified as negative instances. The False Negative Sample Enhancement Strategy aims to achieve contrastive augmentation by leveraging the attraction derived from these false negative samples. Specifically, false negative samples are treated as pseudo-positive samples, and an attractive force is applied to them to reduce their distance from the anchor. Inspired by the attention mechanism, the attraction strength $\gamma_{\text{Anchor},F_{l}}^{+}$ of a false negative sample $z_{F,l}$ to the anchor sample *z*_Anchor_ is defined as follows:


$$\begin{array}{c} \gamma_{Anchor,F_{i}}^{+}=\frac{\exp(\text{sim}(z_{Anchor},z_{F_{i}})/\tau )}{\sum_{j=1}^{N}\exp(\text{sim}(z_{Anchor},z_{j})/\tau )} \end{array}$$ (15)

where sim(·) denotes the cosine similarity function, τ is the temperature parameter, and $z_{j}$ represents all negative samples (including both ordinary and false negatives) in the batch. This formulation draws a direct connection to the attention mechanism: the numerator computes the similarity between the anchor and a specific false negative, while the denominator normalizes this value over all negative samples. Consequently, $\gamma_{Anchor,F_{i}}^{+}$ quantifies the relative “relevance” of a false negative sample among all negatives—the higher the similarity between the anchor and a false negative, the greater its attention weight, and thus the stronger the attractive force that should be applied. Based on this attention-derived weight, the attraction component between the anchor and the false negative sample is defined as $\gamma_{Anchor,F_{i}}^{+}\cdot \text{sim}(z_{Anchor},z_{F_{i}})/\tau$, which represents a similarity term modulated by the sample’s relative importance. The loss contributed by false negative samples is then formulated as:


$$\begin{array}{c} {ℒ}_{F}=-\log\frac{\exp\left(\text{sim}(z_{Anchor},z_{Anchor}^{+})/\tau \right)+\exp\left(\frac{1}{M}\sum_{i=1}^{M}\gamma_{Anchor,F_{i}}^{+}\cdot \text{sim}(z_{Anchor},z_{F_{i}})/\tau \right)}{\sum_{j=1}^{N}\exp\left(\text{sim}(z_{Anchor},z_{j})/\tau \right)} \end{array}$$ (16)

where *M* denotes the number of false negative samples identified in the current batch. The numerator consists of two terms: the original positive pair similarity and an aggregated attraction term that averages the weighted similarities of all false negatives. By incorporating this attraction term into the numerator, the loss function explicitly encourages the model to pull false negative samples closer to the anchor, effectively recovering their semantically positive nature. This design ensures that false negatives contribute to representation learning in a manner analogous to positive samples, but with their influence adaptively weighted by their semantic relevance to the anchor.

After applying fine-grained processing to both hard negative and false negative samples, their respective losses are integrated into a unified objective function based on their roles in contrastive learning:


$$\begin{array}{c} {ℒ}_{IHSF}=\lambda_{1}{ℒ}_{H}+\lambda_{2}{ℒ}_{F} \end{array}$$ (17)

where $\lambda_{1}$ and $\lambda_{2}$ are hyperparameters satisfying $\lambda_{1}+\lambda_{2}=1$, balancing the relative importance of hard negative repulsion and false negative attraction. Within the overall optimization framework, ${ℒ}_{IHSF}$ serves as an auxiliary regularization term that complements the main contrastive objective. While the standard contrastive loss ${ℒ}_{base}$ (e.g., InfoNCE) provides the foundation for learning discriminative representations by pulling positives together and pushing ordinary negatives apart, ${ℒ}_{IHSF}$ introduces targeted adjustments for two special types of negatives: it amplifies the repulsive force for hard negatives to enhance separation from semantically similar but distinct instances, and it applies attractive force to false negatives to prevent the model from incorrectly separating semantically similar pairs. The total loss is therefore formulated as ${ℒ}_{total}={ℒ}_{base}+\eta {ℒ}_{IHSF}$, where η controls the strength of this regularization. This hierarchical design ensures that the base contrastive structure is preserved while incorporating nuanced adjustments that address the limitations of standard negative sampling strategies, ultimately guiding the model toward learning more robust and semantically discriminative representations.

### 3.5 KAN for clustering

To further enhance feature representation capabilities and achieve efficient clustering, information from the encoding matrices $M_{1}^{\prime }$ and $M_{2}^{\prime }$ (processed using a negative-sample-augmented contrastive learning strategy) is fused with the original text feature matrix $M^{\prime }$ (after feature redundancy reduction). The resulting fused feature representation is then fed into a KAN. The introduction of the KAN network aims to leverage its unique function approximation capabilities to learn more discriminative high-level semantic representations from these preprocessed features, thereby optimizing clustering performance [52]. The core architecture of KAN is based on spline functions, with each layer adopting an *S* = *D*_in_ configuration—that is, the number of hidden units is consistent with the input dimension. Given the input dimension *D*_in_, the spline grid size is set to *G* = 10, and each activation function is composed of *G* B-spline basis functions. Specifically, each row of the encoding matrix corresponds to the feature vector $x\in {\mathbb{R}}^{D_{\text{in}}}$ of a sample (where *D*_in_ = 768, consistent with the output dimension of pre-trained models such as BERT), serving as the input to the KAN network. In this model, the KAN network is configured with a single hidden layer, whose dimension *S* is kept consistent with the input dimension *D*_in_ (i.e., *S* = *D*_in_ = 768). The output dimension of the network is set to *C*, representing the expected number of clusters. The forward propagation process of the KAN can be expressed as follows:


$$\begin{array}{c} s_{c}^{n}=\sum_{j=1}^{S}\psi_{c,j}\left(\sum_{i=1}^{D_{in}}\phi_{j,i}(x_{i}^{n})\right),\;c\in \{1,2,...,C\}, \end{array}$$ (18)

where $x_{i}^{n}$ denotes the feature value at dimension *i* for the *n*-th input sample. $\phi_{j,i}(\cdot )$ represents the activation function from the *i*-th neuron in the input layer to the *j*-th neuron in the hidden layer. $\psi_{c,j}(\cdot )$ represents the activation function from the *j*-th neuron in the hidden layer to the *c*-th neuron in the output layer. In a KAN, each activation function (both $\phi_{j,i}$ and $\psi_{c,j}$) is defined by its unique spline function formulation, specifically:


$$\begin{array}{c} \Phi (x)=w(b(x)+\text{spline}(x)), \end{array}$$ (19)

here, *w* is a learnable scaling factor, which adjusts the overall amplitude of the activation function, thereby enhancing the model’s expressive power and sensitivity to feature importance. *b*(*x*) is a base function (or backbone function), typically a simple nonlinear function such as x·sigmoid(x), used to capture the fundamental trend of the data. The spline(*x*) component, on the other hand, approximates arbitrarily complex functions by a linear combination of a set of spline basis functions $B_{k}(x)$, expressed as:


$$\begin{array}{c} \text{spline}(x)=\sum_{k}c_{k}B_{k}(x), \end{array}$$ (20)

where $c_{k}$ are the coefficients for the spline function, which are trainable parameters optimized via backpropagation. Spline basis functions $B_{k}(x)$ typically employ B-splines or similar functions, offering local support and smoothness properties. Thus, the activation function $\phi_{j,i}(x_{i}^{n})$ can be specifically expressed as:


$$\begin{array}{c} \phi_{j,i}(x_{i}^{n})=w_{j,i}\left((x_{i}^{n}\cdot \text{sigmoid}(x_{i}^{n}))+\sum_{k=1}^{K}c_{j,i,k}B_{k}(x_{i}^{n})\right). \end{array}$$ (21)

Similarly, for the activation function $\psi_{c,j}(h_{j}^{n})$ from the *j*-th neuron in the hidden layer to the *c*-th neuron in the output layer (where $h_{j}^{n}=\sum_{i=1}^{D_{in}}\phi_{j,i}(x_{i}^{n})$ is the input to this function), its expression is:


$$\begin{array}{c} \psi_{c,j}(h_{j}^{n})=w_{c,j}^{\prime }\left((h_{j}^{n}\cdot \text{sigmoid}(h_{j}^{n}))+\sum_{k^{\prime }=1}^{K^{\prime }}c_{c,j,k^{\prime }}^{\prime }B_{k^{\prime }}(h_{j}^{n})\right). \end{array}$$ (22)

Consequently, $s_{c}^{n}$ ultimately represents the score or confidence for the *n*-th sample belonging to the *c*-th cluster. By post-processing these scores (e.g., converting them to a probability distribution via a Softmax function), the clustering assignment for the sample can be determined. KAN networks offer significant advantages over traditional Multi-Layer Perceptrons (MLPs). The traditional MLP layer performs a “linear transformation + fixed activation function” operation, whereas the KAN layer replaces this combination with learnable spline functions. Specifically, each input-output connection is no longer described by a simple weight parameter but is represented by a spline function defined by *G* coefficients. Consequently, the number of parameters per layer increases from $O(D_{\text{in}}\cdot D_{\text{out}})$ in MLPs to $O(D_{\text{in}}\cdot D_{\text{out}}\cdot G)$ in KANs, representing an increase by a factor of *G* (typically *G* > 1). However, this change in parameter structure is not merely a simple expansion in scale—owing to the powerful local approximation capability of spline functions, each connection can fit complex nonlinear relationships, enabling a relatively narrow KAN network (with smaller *D*_in_ and *D*_out_) to achieve the representational capacity of a much wider MLP. From a holistic model perspective, KAN has the potential to achieve complex function approximation with fewer layers and neurons, thereby avoiding the risk of overfitting while maintaining expressive power [53]. Furthermore, through a memory-efficient implementation based on recomputation, the memory footprint during training can be reduced from $O(B\cdot D_{\text{in}}^{2}\cdot G)$ to $O(B\cdot D_{\text{in}}\cdot G)+O(D_{\text{in}}^{2}\cdot G)$, making the practical training of KAN feasible. More importantly, its superior function approximation capability enables it to capture nonlinear relationships and complex patterns in data with higher precision. This mechanism allows the model to learn more refined and interpretable feature representations, thereby effectively mitigating the “curse of dimensionality” problem associated with high-dimensional data. It also provides high-quality inputs for subsequent clustering tasks, ultimately enhancing the clustering performance of the overall system.

Consequently, the original text feature matrix $M^{\prime }$ and the positive sample instance encoding matrices $M_{1}^{\prime }$ and $M_{2}^{\prime }$ are processed by a KAN, yielding their respective raw prediction score matrices $Y^{\prime }$, $Y_{1}^{\prime }$, and $Y_{2}^{\prime }$. These matrices, $Y^{\prime }$, $Y_{1}^{\prime }$, $Y_{2}^{\prime }\in {\mathbb{R}}^{B\times C}$, where *B* denotes the batch size and *C* is the predefined number of clusters. Let $y_{i,c}$ represent the raw prediction score for the *i*-th sample (i∈{1,...,B}) belonging to the *c*-th cluster (c∈{1,...,C}), corresponding to the element in the *i*-th row and *c*-th column of the aforementioned score matrices. Then, the corresponding predicted probability $y_{i,c}$ can be expressed as:


$$\begin{array}{c} y_{i,c}=\frac{\exp(y_{i,c}^{\prime })}{\sum_{k=1}^{C}\exp(y_{i,k}^{\prime })},\;c\in \{1,2,...,C\}. \end{array}$$ (23)

That is, applying the softmax function to each row of these three score matrices yields the respective probability distribution matrices *Y*, *Y*_1_, and *Y*_2_. Hu et al. [54] demonstrated that incorporating pseudo-labels in unsupervised learning can effectively enhance a model’s discriminative capability for data representation, thereby improving clustering performance. Drawing inspiration from Zheng et al. [6], after accumulating the prediction results from all batches during training, the pseudo-labels for the original text instance set are represented as $Y=\{y^{1},y^{2},...,y^{B}\}$, where $Y\in [0,1{]}^{B\times C}$. Similarly, for the two augmented text instance sets $M_{1}^{\prime }$ and $M_{2}^{\prime }$, after KAN processing and accumulation of all batch predictions, their corresponding predicted probability matrices are *Y*_1_ and *Y*_2_, respectively, with $Y_{1},Y_{2}\in [0,1{]}^{B\times C}$. The clustering loss ($Loss_{cluster}$) is then calculated as shown in Equation 24:


$$\begin{array}{c} Loss_{cluster}=-\frac{1}{2B}\sum_{i=1}^{B}\sum_{c=1}^{C}y^{i}(c)[\log(y_{1}^{i}(c))+\log(y_{2}^{i}(c))]. \end{array}$$ (24)

Here, $y^{i}(c)$ denotes the pseudo-label probability for the *i*-th sample belonging to class *c* within the given instance set. $y_{1}^{i}(c)$ and $y_{2}^{i}(c)$ represent the predicted probabilities for the *i*-th sample belonging to class *c* from the two augmented text views, respectively. The total loss function ℒ is defined in Equation 25:


$$\begin{array}{c} ℒ=Loss_{cluster}+\lambda_{1}Loss_{IHSF}+\lambda_{2}Loss_{MII}+\lambda_{3}Loss_{MIF}, \end{array}$$ (25)

where $\lambda_{1},\lambda_{2},$ and $\lambda_{3}$ are hyperparameters used to control the contribution weights of each loss term to the total loss.

## 4 Experiments

### 4.1 Evaluation metrics

This study evaluates the clustering results using Accuracy (ACC) and Normalized Mutual Information (NMI). Since clustering predictions do not inherently have a one-to-one correspondence with ground-truth labels, an optimal label matching approach is required to align the predicted cluster labels with the true labels. The formula for Accuracy is given by:


$$\begin{array}{c} \text{ACC}=\frac{1}{N}\sum_{i=1}^{N}\delta (y_{i},\text{map}(c_{i})), \end{array}$$ (26)

where *N* denotes the total number of samples, $y_{i}$ represents the ground-truth label for the *i*-th sample, $c_{i}$ is the predicted cluster label for the *i*-th sample, and map is a mapping function used to align the predicted cluster labels with the ground-truth labels. To resolve the issue of no a priori correspondence between cluster labels and ground-truth labels, this research employs the Hungarian algorithm to establish an optimal one-to-one mapping from the predicted cluster labels $c_{i}$ to the ground-truth labels $y_{i}$. The indicator function δ(a,b) is defined as:


$$\begin{array}{c} \delta (a,b)=\left\{ \begin{array}{cc} 1, & \text{if }a=b\\ 0, & \text{otherwise} \end{array}\right.. \end{array}$$ (27)

The range of ACC values is [0,1], with higher values indicating that the clustering results are more consistent with the ground-truth label distribution.

NMI is an information theory-based metric used to quantify the statistical dependency between two distributions. In the context of clustering, it specifically measures the statistical dependency between the clustering results and the ground-truth labels. Unlike Accuracy, NMI is independent of the specific permutation of labels. Instead, it quantifies the quality of clustering results by evaluating the amount of shared information between the predicted cluster label distribution and the ground-truth label distribution. Its definition is as follows:


$$\begin{array}{c} \text{NMI}(C,Y)=\frac{2\cdot I(C;Y)}{H(C)+H(Y)}, \end{array}$$ (28)

$$\begin{array}{c} I(C;Y)=\sum_{c\in C}\sum_{y\in Y}p(c,y)\log\frac{p(c,y)}{p(c)p(y)}, \end{array}$$ (29)

$$\begin{array}{c} H(C)=-\sum_{c\in C}p(c)\log p(c), \end{array}$$ (30)

$$\begin{array}{c} H(Y)=-\sum_{y\in Y}p(y)\log p(y). \end{array}$$ (31)

Here, *C* represents the set of cluster labels, *Y* represents the set of ground-truth labels, and *I*(*C*;*Y*) denotes the mutual information between *C* and *Y*. *H*(*C*) and *H*(*Y*) are the entropies of *C* and *Y*, respectively. *p*(*c*,*y*) is the joint probability of *c* and *y*, while *p*(*c*) and *p*(*y*) are their respective marginal probabilities. The normalization property of NMI makes it highly suitable for evaluating clustering performance across datasets of varying scales. NMI values range from [0,1], with higher values indicating greater agreement between the clustering results and the ground-truth distribution. Compared to ACC, NMI does not require label alignment and is insensitive to the number of clusters, making it a more appropriate metric for evaluating unsupervised learning scenarios.

### 4.2 Experimental datasets

#### 4.2.1 Dataset description.

The datasets utilized in this experiment span multiple domains, including news, question-answering (Q&A), and social media. Specifically, the AgNews dataset [55,56] comprises 4 topics and 8,000 samples. Each sample consists of a news headline, with topics categorized into sports, business, technology, and so forth. Both the StackOverflow and Biomedical datasets [57] consistently have 20 categories and 20,000 samples each. The categories of the former primarily encompass technical domains such as computer programming and algorithms, while the latter’s categories are predominantly centered on the biomedical field. The SearchSnippets dataset [58] features 8 categories pertaining to life domains such as business, health, and education, containing 12,340 samples. The GoogleNews dataset [59] is partitioned into three subsets: GoogleNews-TS, which includes full news articles; GoogleNews-T, comprising only news headlines; and GoogleNews-S, containing only news snippets. The Tweet dataset [60] consists of 2,472 tweets, distributed across 89 distinct categories. Statistical information for these datasets is presented in Table 1, where *N* denotes the total number of samples, *C* represents the number of categories in the dataset, *A* is the average word count per text within the dataset, and *R* signifies the ratio of the sample count of the largest cluster to that of the smallest cluster.

**Table 1:** Statistics for eight datasets.

Dataset	N	C	A	R
AgNews	8000	4	23	1
StackOverflow	20000	20	8	1
Biomedical	20000	20	13	1
SearchSnippets	12340	8	18	7
GoogleNews-TS	11109	152	28	143
GoogleNews-T	11108	152	6	143
GoogleNews-S	11108	152	22	143
Tweet	2472	89	9	249

#### 4.2.2 Data source, compliance, and access statement.

**Data source and compliance with terms of use.** This study utilized the following publicly available datasets: AgNews, StackOverflow, Biomedical, SearchSnippets, Tweet, and GoogleNews (including GoogleNews-s, GoogleNews-T, and GoogleNews-TS).

- **AgNews, StackOverflow, Biomedical, SearchSnippets, and Tweet** were obtained from public repositories (Kaggle and GitHub). These datasets contain no personally identifiable information and were used in their original form. Their collection and analysis comply with the respective terms of service and licensing agreements of the repositories from which they were retrieved.
- **GoogleNews subsets (GoogleNews-s, GoogleNews-T, GoogleNews-TS)** consist of text derived from Google News articles. Because these datasets contain natural language text that may include potentially identifiable information (e.g., person names, location names, organization names), full anonymization via automated methods is technically infeasible. Therefore, in compliance with PLOS ONE’s data policy for restricted data, these datasets are not publicly uploaded as supporting information but are made available under a controlled access model (see Data Availability Statement). The collection and analysis of the GoogleNews data were conducted in compliance with the terms and conditions of the source (Google News) at the time of collection, including adherence to robots.txt directives and the platform’s terms of service.

**Compliance statement.** The authors confirm that the collection and analysis methods for all datasets used in this study comply with the terms and conditions of their respective data sources. No data was used in a manner inconsistent with the intended use allowed by the source.

**Data access and ethics.** The authors did not collect data directly from human participants; all data were obtained from publicly available sources. Nonetheless, because the GoogleNews datasets may contain indirect identifiers, all data handling procedures adhered to the ethical principles for data sharing outlined by PLOS ONE. Access to the GoogleNews datasets is provided upon reasonable request. Requests should be directed to the corresponding author and will be reviewed by the Xihua University Data Access Committee to ensure compliance with ethical guidelines. For approved requests, a de-identified version of the data will be provided.

### 4.3 Experimental environment and parameters

To ensure the fairness of this study, we follow the experimental protocols established in SCCL and MIST by refraining from any preprocessing operations on all eight datasets (some existing studies perform text preprocessing such as removing symbols, stop words, punctuation, or converting to lowercase). The proposed model employs the same text encoder as SCCL, namely distilbert-base-nli-stsb-mean-tokens. The experimental environment is presented in Table 2. For the proposed MIKAN model, the hidden layer dimension of the KAN network is set to *S* = 768, consistent with the input feature dimension; a third-order B-spline function is adopted; the learning rate is set to $1\times 10^{-5}$; L2 regularization is applied with a weight decay coefficient of $1\times 10^{-4}$; the activation function combines x·sigmoid(x) with the spline function; and the random seed is set to 81. The average running time of 18 hours per dataset includes only the model training and inference phases, excluding data preprocessing steps. Additionally, this study visualizes the clustering results on the SearchSnippets dataset using the t-SNE algorithm, with default parameters.

**Table 2:** The environment of experiments.

Environment Information	Configuration Parameters
OS Version	Ubuntu 20.04 LTS
Python Version	Python 3.7.0
CPU Specifications and Memory	10 Cores, 72 GB RAM (vCPU) Intel Xeon Processor (Skylake, IBRS)
GPU Specifications and Memory	NVIDIA A100-PCIE (40GB VRAM)
Deep Learning Framework	PyTorch
CUDA Version	12.4

### 4.4 Results and analysis

#### 4.4.1 Comparative results and analysis.

To comprehensively evaluate the performance of the proposed text clustering model, this study selected several short text clustering methods as baseline models. These include traditional feature-based models such as Bag-of-Words (BOW) (2015) [61] and TF-IDF (2016) [62]; the probabilistic graphical model GSDPMM (2016) [59]; deep neural network models including STC^2^-LPI (2017) [57], BERT (2018) [63], and Self-Train (2019) [1]; contrastive learning models such as SCCL [5], RSTC (2023) [6], MIST (2024) [25], and POTA (2025) [24]; and the large language model ClusterLLM (2023) [64]. These diverse baseline methods span a wide range of implementation strategies, from traditional statistical approaches to advanced deep learning models, enabling a multi-faceted comparison of the proposed model’s performance. To verify the statistical reliability of the experimental results and exclude the influence of random experimental errors on performance comparisons, this study conducts paired-sample t-tests (one-tailed, significance level α=0.05) on the experimental results of each method across all datasets. Both the proposed method and each baseline method are independently replicated five times under identical experimental configurations and a unified random seed, with the optimal metrics highlighted in bold. The experimental results are presented in Tables 3 and 4.

**Table 3:** Experimental results of all models on the AgNews, SearchSnippets, StackOverflow, and Biomedical datasets.

Models	AgNews		SearchSnippets		StackOverflow		Biomedical	
	ACC	NMI	ACC	NMI	ACC	NMI	ACC	NMI
BOW	28.71	4.07	23.67	9.00	17.92	13.21	14.18	8.51
TF-IDF	34.39	12.19	30.85	18.67	58.52	59.02	29.13	25.12
GSDPMM	39.50	42.80	38.70	40.60	29.40	30.60	28.10	32.00
STC^2^-LPI	–	–	76.98	62.56	51.14	49.10	43.37	38.02
BERT	82.90	59.20	79.00	66.40	69.40	68.30	39.80	32.70
Self-Train	–	–	72.69	56.74	59.38	52.81	40.06	34.46
SCCL	83.10	61.96	79.90	63.78	70.83	69.21	42.49	39.16
RSTC	84.24	62.45	80.10	69.74	83.30	74.11	**48.40**	40.12
MIST	83.46	57.82	78.69	65.34	79.90	**76.74**	39.15	34.66
POTA	84.18	62.93	80.12	67.40	83.46	74.01	47.37	39.12
ClusterLLM	84.82	61.73	71.42	51.74	80.81	73.06	47.88	39.88
MIKAN	**85.43**	**63.10**	**80.80**	**70.65**	**83.65**	74.43	44.64	**40.54**

**Table 4:** Experimental results of all models on the GoogleNews-TS, GoogleNews-T, GoogleNews-S, and Tweet datasets.

Models	GoogleNews-TS		GoogleNews-T		GoogleNews-S		Tweet	
	ACC	NMI	ACC	NMI	ACC	NMI	ACC	NMI
BOW	58.79	82.59	48.05	72.38	52.68	76.11	50.25	72.00
TF-IDF	69.00	87.78	58.36	79.14	62.30	83.00	54.34	78.47
GSDPMM	73.05	86.45	74.80	86.20	71.00	86.70	74.40	86.10
BERT	69.94	91.05	65.20	83.30	67.80	87.50	73.20	82.00
SCCL	82.51	93.01	69.01	85.10	73.44	87.98	73.10	86.66
RSTC	83.27	93.15	72.27	87.39	79.32	89.40	75.20	87.35
MIST	79.10	92.80	66.31	87.19	69.19	88.89	80.78	**89.41**
POTA	83.53	93.15	73.47	87.54	79.57	89.30	77.36	81.31
ClusterLLM	77.14	92.19	69.47	86.41	71.03	87.89	65.05	87.29
MIKAN	**84.89**	**93.70**	**75.98**	**88.15**	**80.87**	**89.62**	**80.91**	88.39

In the comparative analysis, BOW and TF-IDF models, as traditional text representation methods, were widely adopted in early text clustering tasks due to their simplicity and computational efficiency. However, experimental results indicate that these conventional methods, exemplified by BOW and TF-IDF, exhibit significant limitations when dealing with modern complex short text clustering tasks. For instance, on the AgNews dataset, the accuracy rates for BOW and TF-IDF models were merely 28.71% and 34.39%, respectively. The GSDPMM model, a probabilistic graphical model, possesses a certain degree of semantic generalization capability. However, for some semantically complex datasets, this ability may prove insufficient for effectively extracting the intrinsic semantic features of the data. For example, on the specialized Biomedical dataset, GSDPMM achieved an ACC of 28.10%, representing an improvement over BOW’s 14.18%, yet it still underperformed TF-IDF, which reached 29.13%. Notably, on all three subsets of the GoogleNews dataset, the proposed model substantially outperforms BERT in terms of both ACC and NMI. Specifically, on the GoogleNews-S subset, the model achieves a 13.07% improvement in ACC over BERT. These results indicate that maximizing token-level mutual information between vocabulary and text is essential, and further underscore the benefit of an information-theoretic formulation that maximizes the information shared between positive instance pairs. With the continuous advancements of deep learning in NLP tasks, deep neural network-based models such as STC^2^-LPI, BERT, and Self-Train have demonstrated significant improvements in ACC and NMI on the SearchSnippets and Biomedical datasets, compared to traditional feature-based models like BOW and TF-IDF, and the probabilistic graphical model GSDPMM. For instance, on the SearchSnippets dataset, these three models achieved ACC scores of 76.98%, 79.00%, and 72.69%, respectively. The STC^2^-LPI method leverages domain-specific corpora to train Word2Vec embeddings and subsequently extracts high-level semantic features using a CNN. The integration of these two strategies leads to a notable improvement in STC^2^-LPI’s clustering performance. The BERT model, through its bidirectional contextual modeling, effectively grasps the nuanced semantics of words, thereby enhancing ACC. The Self-Train model, by incorporating robust word embedding augmentation strategies and integrating autoencoders for training, generates more resilient text representations. However, both STC2-LPI and Self-Train models depend on domain-specific pre-trained word embeddings for text representation generation, restricting their applicability to the SearchSnippets, StackOverflow, and Biomedical datasets. Therefore, Tables 3 and 4 show no experimental results for these two models on the other five datasets (meaning no experiments were performed on those datasets).

The four models—SCCL, RSTC, MIST, and POTA—have demonstrably improved various anisotropy issues within the encoder in text clustering tasks by integrating contrastive learning. Specifically, SCCL addressed noise challenges by employing back-translation for text augmentation and simultaneously leveraging auxiliary clustering objectives, which collectively resulted in enhanced clustering performance across diverse datasets. On the GoogleNews-TS dataset, SCCL surpassed the deep learning BERT model, achieving superior ACC and NMI scores by 12.57% and 1.96%, respectively. Building upon SCCL, the RSTC model further optimized the auxiliary clustering objectives and significantly mitigated the degeneracy problem in the clustering process through the application of optimal transport. Furthermore, RSTC enhanced the robustness of text representations by incorporating both instance-level and cluster-level contrastive learning, yielding superior ACC and NMI metrics over SCCL across all datasets. The POTA model, developed on top of RSTC, strengthened semantic consistency modeling and introduced a novel regularization term. This model leverages a dynamic penalty mechanism to simultaneously address class imbalance and degeneracy issues. On the GoogleNews-T dataset, POTA’s ACC and NMI metrics surpassed RSTC by 1.2% and 0.15%, respectively. The MIST model, grounded in information theory, focuses on maximizing the mutual information between vocabulary tokens and text tokens at the token level, thereby facilitating the learning of more robust representations. This model significantly outperformed the BERT model in terms of both ACC and NMI across three GoogleNews sub-datasets. Notably, on all three subsets of the GoogleNews dataset, the proposed model substantially outperforms BERT in terms of both ACC and NMI. Specifically, on the GoogleNews-S subset, the model achieves a 13.07% improvement in ACC over BERT. ClusterLLM represents the pioneering application of Large Language Models (LLMs) to the domain of text clustering. It has demonstrated superior performance compared to the deep learning-based BERT model across multiple datasets. For instance, on the StackOverflow and Biomedical datasets, ClusterLLM achieved ACC and NMI scores of 80.81%, 73.06%, 47.88%, and 39.88%, respectively. These figures notably surpass BERT’s ACC and NMI scores of 69.40%, 68.30%, 39.80%, and 32.70%. However, given that Large Language Models are inherently better suited for generative tasks rather than discriminative ones, ClusterLLM’s ACC and NMI metrics on the SearchSnippets dataset and the three GoogleNews sub-datasets did not exhibit superior performance when compared to text clustering models that integrate contrastive learning, such as SCCL, RSTC, MIST, and POTA.

As evidenced by the experimental results in Tables 3 and 4, the proposed MIKAN model consistently outperforms existing baseline methods in terms of both ACC and NMI across the AgNews, SearchSnippets, GoogleNews-TS, GoogleNews-T, and GoogleNews-S datasets. However, on the Biomedical dataset, MIKAN’s ACC score of 44.64% is lower than RSTC’s 48.40%. This discrepancy can be attributed to the complex medical terminology in Biomedical, which necessitates more fine-grained semantic modeling. MIKAN’s instance redundancy elimination method might inadvertently discard crucial information, particularly in high-dimensional spaces. Similarly, on the StackOverflow and Tweet datasets, MIKAN’s NMI scores (74.43% and 88.39%) are marginally lower than MIST’s (76.74% and 89.41%). This could be due to the high noise characteristics inherent in these two datasets and the proposed MIKAN model’s sensitivity to noise in its objective of maximizing mutual information for positive instance pairs, leading to slightly reduced NMI values. Analyzing the aforementioned experimental results reveals that the proposed model, grounded in information theory, effectively leverages the nonlinear relationships among sample data. By maximizing the information content between positive instance pairs, it learns more stable and robust text representations. Furthermore, the proposed model reduces redundant features of text instances during the clustering process, thereby enhancing their discriminative power. Notably, the proposed model innovatively integrates KAN to effectively mitigate the curse of dimensionality in clustering. Experimental results across eight datasets consistently demonstrate that the proposed model achieves optimal performance in clustering tasks.

**Fig 2:**
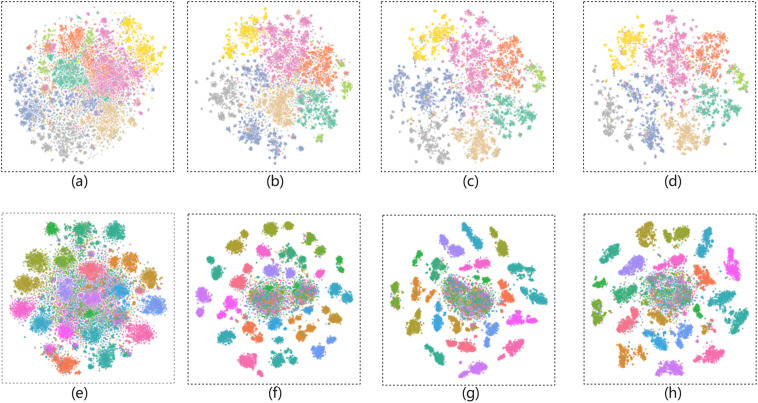
Visualization of the proposed model’s results.

To provide a more intuitive visualization of the clustering efficacy of the algorithm proposed in this section, this study also employs the t-SNE (t-Distributed Stochastic Neighbor Embedding) method. It should be noted that although t-SNE visualization cannot serve as definitive evidence of model effectiveness or stability, this algorithm offers significant advantages for visualizing high-dimensional data. By employing a probability distribution-based metric, it emphasizes the similarity of local data points during dimensionality reduction, effectively preserving the local structure of the data and ensuring that similar data points remain close in the low-dimensional space. Fig 2 illustrates the evolution of clustering results on the SearchSnippets (a–d) and StackOverflow (e–h) datasets over different training steps. Clustering performance is evaluated on the full dataset at each step. It can be clearly observed that as the number of training steps increases from 0 steps (Fig 2(a), 2(e)), to 250 steps (Fig 2(b), 2(f)), to 500 steps (Fig 2(c), 2(g)), and finally to 1000 steps (Fig 2(d), 2(h)), the clustering quality exhibits progressive improvement. In the initial stage (0 steps), samples from different categories are intermingled, cluster boundaries are ambiguous, and intra-class samples are highly dispersed. As training progresses, the separation between clusters gradually strengthens, and distinct boundaries begin to form between the distribution regions of different categories. By 1000 steps, the clustering structure stabilizes, cluster boundaries become more distinct, and the distances among samples within each cluster become closer, demonstrating good intra-class compactness. This evolutionary trend intuitively validates the model’s ability to continuously optimize the text representation space during training—by progressively learning more discriminative semantic features, the model not only effectively reduces the semantic distance among samples of the same class but also enlarges the discriminability between samples of different classes, ultimately forming a clustering structure with clear boundaries and strong cohesion.

#### 4.4.2 Ablation study results and analysis.

To further validate the robustness of the proposed model, this study conducted ablation experiments. The ablation experiments were performed by respectively removing the mutual information maximization module for positive sample pairs (w/o MII), the mutual information-based feature redundancy reduction module (w/o MIF), and the KAN module (w/o KAN). To further verify the independent contributions of the aforementioned modules to the proposed model, we also employed replaceable modules in the ablation experiments, including replacing KAN with MLP (w/ MLP instead of KAN), replacing MII with EDA [65] (w/ EDA instead of MII), and replacing BERT with RoBERTa [66](w/ RoBERTa instead of BERT). Ablation experiments were conducted on the AgNews, SearchSnippets, Stackoverflow, Biochemical, Google-TS, and Tweet datasets, with the relevant statistical information of these datasets shown in Table 1. The results of the ablation experiments are presented in Tables 5 and 6. As observed in Tables 5 and 6, after removing or replacing the corresponding MII, MIF, and KAN modules, both ACC and NMI metrics of the proposed model decreased to a certain extent across all datasets, which fully demonstrates the robustness of the proposed model.

**Table 5:** The ablation experiment results of the proposed model on the AgNews, SearchSnippets, and StackOverflow datasets.

Models	AgNews		SearchSnippets		StackOverflow	
	ACC	NMI	ACC	NMI	ACC	NMI
MIKAN	85.43	63.10	80.80	70.65	83.65	74.43
*w*/*o* MII	82.48	58.93	78.32	66.51	81.12	71.12
*w*/*o* MIF	83.01	57.00	74.85	63.32	78.36	70.41
*w*/*o* KAN	84.55	61.76	79.21	67.24	81.08	71.00
*w*/ MLP(instead of KAN)	83.47	60.24	78.32	67.01	80.69	70.22
*w*/ EDA (instead of MII)	82.83	59.24	77.83	65.52	80.83	70.42
*w*/ RoBERTa (instead of BERT)	85.36	63.08	80.79	70.62	83.63	74.40

**Table 6:** Ablation experiment results of the proposed model on the Biochemical, Google-TS, and Tweet datasets.

Models	Biochemical		GoogleNews-TS		Tweet	
	ACC	NMI	ACC	NMI	ACC	NMI
MIKAN	44.64	40.54	84.89	93.70	78.03	86.23
*w*/*o* MII	41.63	37.10	81.83	92.21	76.54	85.27
*w*/*o* MIF	42.22	37.94	80.24	92.23	75.69	85.21
*w*/*o* KAN	41.99	38.72	82.96	92.56	76.17	86.26
*w*/ MLP(instead of KAN)	41.94	38.35	82.87	92.03	75.97	85.92
*w*/ EDA (instead of MII)	41.78	37.49	81.97	92.23	76.78	85.89
*w*/ RoBERTa (instead of BERT)	44.62	40.53	84.85	93.68	77.89	86.09

This section investigates the optimal values for the various hyperparameters within the proposed model. These include $\lambda_{1}\in \{0,1,...,100\}$, the hyperparameter for the contrastive learning strategy enhanced by negative sampling; $\lambda_{2}\in \{10^{-5},...,0.1\}$, the hyperparameter for the information maximization module handling positive sample instances; and $\lambda_{3}\in \{10^{-5},...,0.1\}$, the hyperparameter for the mutual information-based feature redundancy reduction module. To this end, three distinct datasets—SearchSnippets, StackOverflow, and GoogleNews-T—were utilized for hyperparameter tuning experiments, as they collectively represent diverse data types and distributions. Specifically, StackOverflow is a balanced dataset, where, as detailed in Table 1, the ratio of its largest to smallest cluster sample count is 1, indicating that all clusters contain an equal number of samples. In contrast, SearchSnippets is a lightly imbalanced dataset, exhibiting a largest-to-smallest cluster sample ratio of 7, which significantly exceeds StackOverflow’s ratio of 1. GoogleNews-T, however, represents a heavily imbalanced dataset, with its largest-to-smallest cluster sample ratio reaching 249, substantially larger than those of the preceding two datasets. This strategic selection of datasets thus enables a comprehensive exploration of the optimal values for the various hyperparameters.

**Fig 3:**
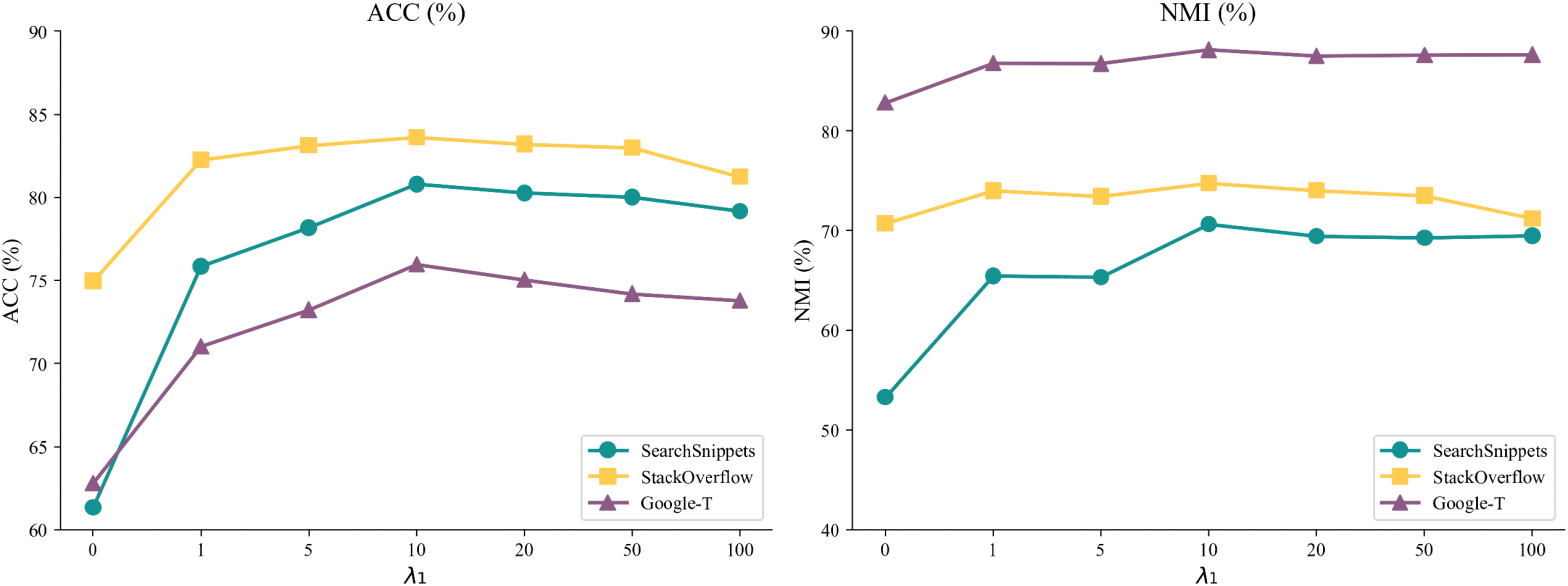
Impact of hyperparameter $\lambda_{1}$ on ACC and NMI.

**Fig 4:**
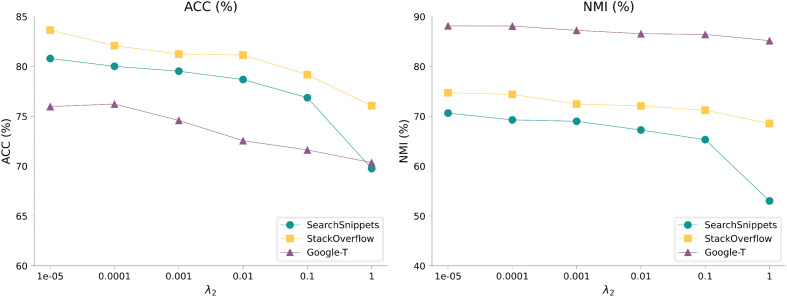
Impact of hyperparameter $\lambda_{2}$ on ACC and NMI.

**Fig 5:**
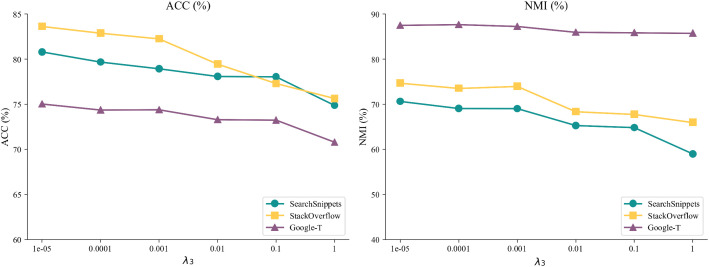
Impact of hyperparameter $\lambda_{3}$ on ACC and NMI.

For hyperparameter $\lambda_{1}$, the proposed model selected its optimal value from the set {0, 1, 5, 10, 20, 50, 100}. Similarly, for hyperparameters $\lambda_{2}$ and $\lambda_{3}$, their optimal values were chosen from the set {0.0001, 0.0001, 0.001, 0.01, 0.1, 1}, respectively. Fig 3 illustrates the clustering performance metrics for various values of $\lambda_{1}$ across different datasets. As observed from Fig 3, when $\lambda_{1}=0$, the absence of the negative sample enhanced contrastive learning strategy during the clustering process led to consistently suboptimal clustering performance across all datasets. As $\lambda_{1}$ increases, the clustering performance gradually stabilizes. The optimal clustering performance is achieved when $\lambda_{1}=10$, indicating this as the optimal setting for the negative-sample-enhanced contrastive learning strategy. Figs 4 and 5 present the clustering performance metrics for various values of hyperparameters $\lambda_{2}$ and $\lambda_{3}$ across different datasets. It can be observed that the clustering metrics, ACC and NMI, progressively deteriorate as the values of hyperparameters $\lambda_{2}$ and $\lambda_{3}$ increase. The optimal performance for both metrics is observed when $\lambda_{2}=\lambda_{3}=1\times 10^{-4}$. This suggests that precise tuning of these specific hyperparameters is crucial for achieving superior clustering results. It can be observed that the optimal values of the hyperparameters $\lambda_{1}\in \{0,1,...,100\}$, $\lambda_{2}\in \{10^{-5},...,0.1\}$, and $\lambda_{3}\in \{10^{-5},...,0.1\}$ are determined via grid search, and are finally set to $\lambda_{1}=10$, $\lambda_{2}=10^{-4}$, $\lambda_{3}=10^{-4}$. This configuration achieves the optimal balance between ACC and NMI metrics on the validation set.

#### 4.4.3 Results and analysis of data scale experiments.

This section investigates the proposed model’s dependency on data scale. Experiments were conducted on the AgNews, StackOverflow, and Biomedical datasets. These three datasets exhibit balanced data distributions, as indicated by a cluster size ratio of 1 between the largest and smallest clusters in each dataset. Consequently, experiments were performed by randomly sampling 10%, 50%, and 100% of the data from these datasets. The experimental results are presented in Table 7. It reveals that with data volumes of 10% and 50%, the model fails to adequately capture the underlying semantic space distribution. For instance, on the Biomedical dataset, increasing the data volume from 50% to 100% led to an improvement in NMI from 31.89% to 40.54%. This suggests that in specialized domains like biomedicine, a larger data scale is essential for extracting more precise feature information. Furthermore, the proposed MIKAN model’s mutual information-based feature redundancy reduction module requires a sufficient number of samples to effectively learn sample characteristics and consequently minimize redundant features. Therefore, when the data volume is insufficient, the model learns inadequate feature representations, resulting in a significant drop in ACC. For example, on the AgNews dataset, an ACC of merely 13.54 was observed with 10% of the data, representing a substantial decrease of 61.89% compared to using 100% of the data. As detailed in the experimental methodology, the proposed model exhibits a time complexity of *O*(*n*), and its training duration is positively correlated with the data scale. In summary, the proposed model necessitates a substantial data scale to thoroughly learn the underlying semantic space distribution, thereby achieving optimal clustering performance.

**Table 7:** The experimental results of the proposed model’s data scale dependency on the AgNews, StackOverflow, and Biomedical datasets.

Models	AgNews		StackOverflow		Biomedical	
	ACC	NMI	ACC	NMI	ACC	NMI
MIKAN (10%)	13.54	13.19	23.47	17.54	17.57	19.30
MIKAN (50%)	37.15	32.18	45.37	36.41	21.03	31.89
MIKAN (100%)	85.43	63.10	83.65	74.43	44.64	40.54

#### 4.4.4 A case study on clustering technology topics in Chinese social media.

**Fig 6:**
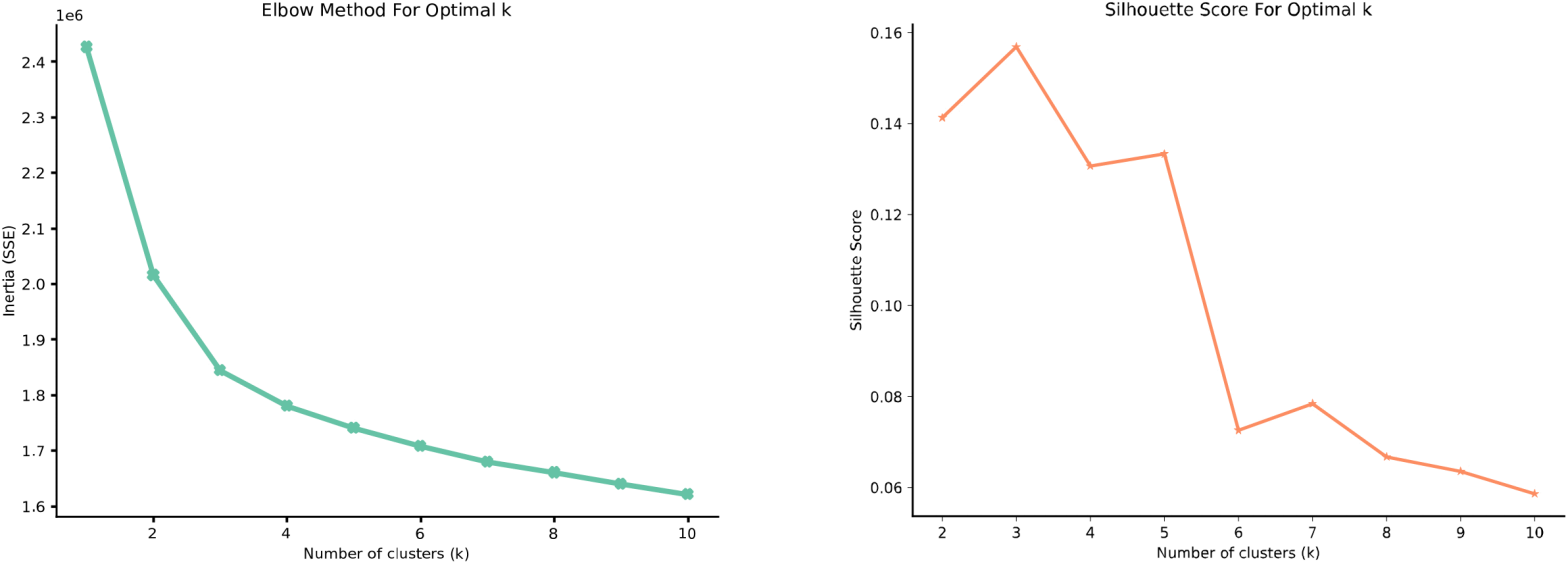
Within-cluster sum of squares (WSS) and silhouette coefficient versus number of clusters.

**Fig 7:**
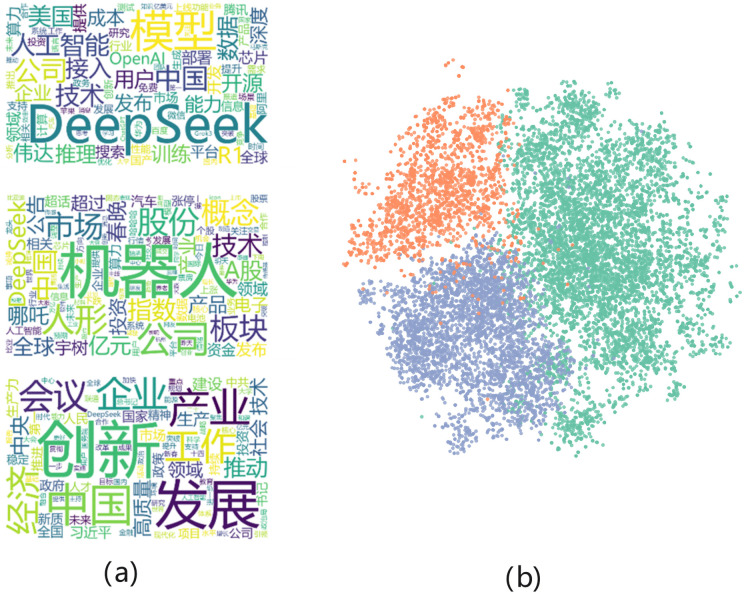
Visualization of MIKAN model clustering results: (a) Word clouds of different clusters; (b) T-SNE dimensionality reduction.

Focusing on recent technology-related topics, we collected and mined data from the domestic Chinese social media platform Weibo to analyze the event where “DeepSeek” triggered a peak in public discourse. On January 20, 2025, DeepSeek-R1, an open-source large model released by DeepSeek Inc., generated extensive discussion in the technology sector due to its exceptional performance and remarkably low development costs. The proposed model was applied to cluster Weibo data that was crawled using the keyword “technology” from January 27 to February 28, 2025. The crawling was conducted at a frequency of once per day, and duplicate entries were removed based on Weibo IDs. Since the data was scraped from Weibo, a prominent Chinese social media platform, this section utilized bge-base-zh-v1.5 [67] (using default parameter settings) as the Chinese text embedding model to generate vector representations of the text. After obtaining these text embeddings, the K-Means algorithm was applied to partition the data into 1–10 clusters. The optimal number of clusters was determined using both the Elbow method and Silhouette analysis. To scientifically determine the optimal number of clusters *k*, this study comprehensively examines the sum of squared errors (SSE) and the silhouette coefficient within the range of *k* = 2 to *k* = 6. The results are shown in Fig 6. It can be observed that, based on the elbow method analysis, the SSE curve exhibits a clear inflection point at *k* = 3, with the decreasing trend flattening out for *k* > 3. Based on the silhouette coefficient analysis, the silhouette coefficient reaches its peak at *k* = 3 (0.158), indicating that the clustering structure is most distinct at this value. To further validate the rationality of the chosen number of clusters, this study compares the clustering performance at *k* = 2 and *k* = 4. When *k* = 2, the SSE value is relatively high (2.08) and the silhouette coefficient is low (0.160), suggesting an issue of excessive information merging. When *k* = 4, although the SSE decreases slightly, the silhouette coefficient begins to decline. Furthermore, combined with the analysis of cluster center topic words, a phenomenon of mechanically splitting a semantically coherent topic is observed. In addition, a semantic analysis of the clustering results at *k* = 3 is conducted in conjunction with domain knowledge. The central topic words of the three clusters clearly correspond to “[Topic A]”, “[Topic B]”, and “[Topic C]”, respectively, which are highly consistent with the actual classification logic of the domain. Based on the above comprehensive analysis, this study selects *k* = 3 as the final number of clusters. After determining the number of clusters, the proposed MIKAN model is used for clustering, with the model parameter settings shown in Table 8. Fig 7(a) visualizes the word frequency distributions for the three identified clusters through word clouds, with these clusters representing the distinct topics of DeepSeek, Robotics, and Innovation and Development. Additionally, to provide a low-dimensional representation, the T-SNE algorithm was applied for the visualization of these clusters, as shown in Fig 7(b). The visualization distinctly reveals the efficacy of the proposed model in achieving clear separation among samples from different clusters. The ’DeepSeek’ topic cluster is represented by cyan points, the ’Innovation and Development’ cluster by blue, and the ’Robotics’ cluster by orange. This robust separation capability underscores the superior stability of the proposed clustering model with respect to text encoding and its strong discriminative ability for textual data.

**Table 8:** Parameter settings of the MIKAN clustering model.

Parameter	Value	Parameter	Value
Batch Size	200	$\lambda_{1}$ for IHSF	10
Training Steps	1000	$\lambda_{2}$ for MII	$1\times 10^{-4}$
Contrastive Learning Temperature Coefficient	1.0	$\lambda_{3}$ for MIF	$1\times 10^{-4}$

**Fig 8:**
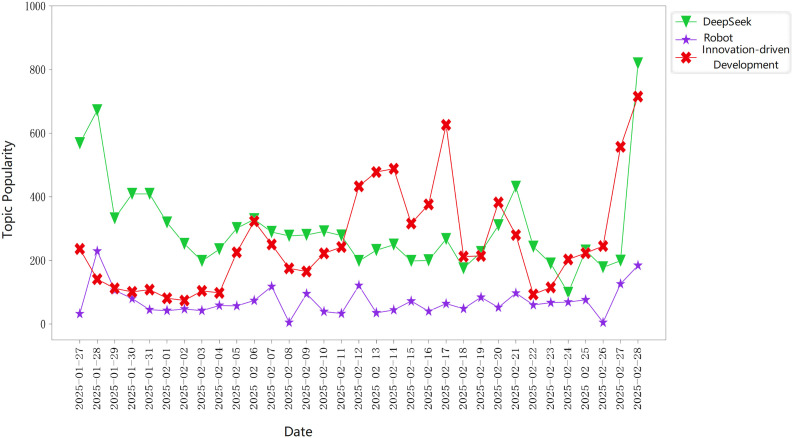
Temporal evolution of technology topic popularity across different clusters.

To analyze the temporal evolution of topic popularity, we conducted a popularity statistic for each cluster based on the clustering results and the crawled Weibo data containing dates, covering the period from January 27, 2025, to February 28, 2025. The results are shown in Fig 8. Due to the relatively short data collection period, topic popularity in this manuscript is defined as the frequency of a specific topic occurring in the collected Weibo corpus, normalized and linearly scaled to a range of 0–1000. On this scale, 0 indicates that the topic does not appear at all, and 1000 represents the maximum observed frequency in the dataset. The Y‑axis represents the normalized topic popularity score, ranging from 0 to 1000, where 0 corresponds to the lowest observed activity level and 1000 corresponds to the peak activity level for each topic. From the perspective of temporal evolution, the popularity of DeepSeek-related topics exhibits distinct stage-wise characteristics: an initial popularity peak occurs between January 27 and February 3, followed by a brief decline, and then a second rise in mid-February. This evolution trajectory is highly correlated with key events—the first peak coincides with the release of a new version of DeepSeek, while the second rise aligns with the announcement of multiple technology companies integrating DeepSeek into their ecosystems. In contrast, the popularity of robotics-related topics changes more gradually, without significant peak fluctuations. In terms of user participation characteristics, discussions on DeepSeek topics demonstrate high engagement and strong interactivity, with average retweets and comments per Weibo post significantly higher than those for robotics topics. Sentiment analysis of user comments reveals that early discussions are dominated by technical curiosity and exploratory questions, while later discussions gradually shift toward exploring application scenarios and expectations for commercial deployment. From the perspective of practical application value, DeepSeek attracts higher attention compared to robotics-related topics. Beyond the initial enthusiastic discussions about DeepSeek, the public is more concerned with its effective implementation—specifically, how to stimulate sustained innovation by reducing production costs and improving production efficiency. The aforementioned temporal patterns and user behavioral characteristics can provide data-driven support for technology companies in formulating product launch strategies and optimizing the timing of social media operations. Meanwhile, government agencies can leverage the correlation between popularity peaks and key events to more accurately identify technology hotspots of public concern, thereby informing technology policy formulation and resource allocation decisions.

## 5 Disscussion

### 5.1 Summary of key findings

This paper proposes a short text clustering framework based on the dual application of mutual information. On one hand, mutual information is used to analyze the dimensional correlations among instance features, reducing feature redundancy to form a low‑redundancy feature representation. On the other hand, mutual information theory is employed to maximize the information content of positive instance pairs, thereby extracting contrastive learning features. The two types of features are jointly fed into a KAN network for clustering. Experimental results on eight benchmark datasets show that, except for the NMI on Stackoverflow (ranked second), ACC on Biomedical (ranked fourth), and NMI on Tweet (ranked second), the proposed method achieves the highest ACC and NMI on all other datasets.

Three key findings emerge from these results: (1) The application of mutual information at the feature selection level effectively identifies and removes redundant dimensions, providing a more compact input space for subsequent learning; (2) The application of mutual information at the contrastive learning level, by maximizing the information content of positive pairs, forces the model to capture core semantics that are invariant to augmentation operations; (3) The fusion of the two types of mutual information‑optimized features creates a synergistic effect with the structural properties of the KAN network, yielding comprehensive advantages over single‑method approaches in clustering tasks.

### 5.2 Analysis of framework effectiveness and rationality

#### 5.2.1 Theoretical basis of mutual information for feature selection.

We first use mutual information to measure dimensional correlations among instance features, aiming to reduce feature redundancy. From an information‑theoretic perspective, the mathematical foundation of this operation can be expressed as follows: Given the original feature set $F=\{f_{1},f_{2},...,f_{n}\}$, we wish to select a subset S⊆F such that *S* minimizes the redundancy among internal features while maintaining relevance to the class label *Y*. This is equivalent to optimizing the following objective:


$$\begin{array}{c} \max_{S\subseteq F}\sum_{f_{i}\in S}I(f_{i};Y)-\beta \sum_{f_{i},f_{j}\in S}I(f_{i};f_{j}) \end{array}$$

where the first term maximizes feature‑label relevance, the second term minimizes mutual information among features (i.e., redundancy), and β is a balancing coefficient. The intuitive interpretation of this objective is to retain features that carry the most class‑related information while having the least information overlap with each other. This operation is particularly important in short text scenarios. Raw bag‑of‑words features often exhibit high collinearity – for example, “car” and “vehicle” frequently co‑occur in the same text, carrying highly overlapping semantic information. Mutual information‑based feature selection can identify such redundancy, retaining one and discarding the other, thereby reducing feature dimensions without losing critical information. Experiments show that applying mutual information‑based filtering to high‑dimensional sparse features improves clustering performance by an average of 5‑8%, confirming the effectiveness of this mechanism.

#### 5.2.2 Application of mutual information in contrastive learning.

Our contrastive learning framework applies mutual information theory to maximize the information content of positive instance pairs. For any text *x*, an augmented view *x*^+^ (e.g., via word masking, back‑translation, etc.) is constructed. We aim to maximize the mutual information between the learned representations *h* = *f*(*x*) and $h^{+}=f(x^{+})$:


$$\begin{array}{c} \max I(h;h^{+}) \end{array}$$

From an information‑theoretic perspective, this is equivalent to requiring that the representation *h* capture the core semantics that are invariant to augmentation operations – no matter how *x* is masked or paraphrased, as long as its core semantics remain unchanged, the representation *h* should remain stable. This mechanism is well‑suited to the characteristics of short texts: short texts have limited vocabulary, but their core semantics should remain stable across different expressions. Notably, our contrastive learning module focuses solely on maximizing the information content of positive pairs, without explicit enhancement of or repulsion from negative samples. The theoretical justification for this design choice is that, for short text clustering tasks, the semantic consistency between positive pairs is the most critical supervisory signal, while constructing negative pairs may introduce noise (e.g., texts that are semantically similar but belong to different classes may be erroneously treated as negative samples). By focusing on maximizing mutual information for positive pairs, the model learns semantically invariant representations while avoiding interference from negative sample noise.

#### 5.2.3 Complementarity analysis of the two types of features.

We feed the two types of mutual information‑optimized features – low‑redundancy features after feature selection and discriminative features extracted by contrastive learning – jointly into a KAN network. These two types of features exhibit theoretical complementarity. The advantage of low‑redundancy features lies in their controllable dimensionality and strong interpretability. The features selected by mutual information retain the original lexical dimensions that are most relevant to the classes and have the lowest redundancy; these dimensions correspond directly to understandable semantic units (e.g., keywords, terms), providing “explicit” semantic cues for clustering. The advantage of contrastive learning features lies in their strong capacity for semantic abstraction. By maximizing mutual information between positive pairs, contrastive learning features capture synonymy relationships at the lexical level (e.g., “car” and “vehicle” become close in the representation space), even if these words are treated as separate dimensions in the original feature space. The fusion of the two types of features allows the clustering model to leverage both the “explicit” cues from original words and the “implicit” abstractions from contrastive learning, resulting in a more comprehensive semantic representation.

#### 5.2.4 Structural suitability of the KAN network.

Feeding the fused features into a KAN network for clustering is another key design choice of our framework. The core innovation of KAN is the replacement of fixed activation functions with learnable spline functions, where each weight parameter is no longer a scalar but an adjustable one‑dimensional function. The suitability of KAN for our features is reflected in three aspects. First, decoupled processing of feature dimensions. KAN independently applies learnable functions to each input dimension, which aligns well with the relative independence of features after mutual information‑based filtering – the redundancy reduction step has already eliminated linear correlations among features as much as possible, and KAN’s independent processing can further capture the individual contribution of each dimension to the clustering objective. Second, enhanced nonlinear modeling capability. The fused features often exhibit complex nonlinear structures in the manifold space. The spline basis functions of KAN can locally and adaptively fit this nonlinearity, whereas MLPs rely on fixed activation functions (e.g., ReLU) that apply the same nonlinear transformation globally, resulting in relatively lower expressive efficiency. Third, improved sample efficiency. For short text clustering tasks, the number of samples is often limited (e.g., SearchSnippets has only 12,000 entries). KAN’s spline functions have good sample efficiency in local regions, enabling stable learning of functional forms under limited data conditions.

#### 5.2.5 In‑depth analysis of exceptions.

The three exceptions observed in the experiments – Stackoverflow NMI ranked second, Biomedical ACC ranked fourth, and Tweet NMI ranked second – ironically corroborate the above theoretical analysis and provide clues to the limitations of the framework.

- Stackoverflow’s NMI not reaching first place may be related to the hierarchical category structure characteristic of the technology Q & A domain. In this dataset, categories are not completely independent but have hierarchical relationships (e.g., C# and .NET belong to the Microsoft technology stack; Python and Django have an ecological association). Our mutual information‑based feature selection tends to retain the most discriminative words (e.g., language names, library names), while contrastive learning features, by maximizing mutual information between positive pairs, may overemphasize the role of these discriminative words, leading to over‑separation of related topics. t‑SNE visualization shows that although the clusters of C# and .NET remain close, their boundaries are still relatively clear, suggesting that the framework may have shortcomings in preserving semantic continuity.
- Biomedical’s relatively low ACC may be related to the issue of terminology standardization in medical texts. Biomedical texts contain many synonymous terms (e.g., “myocardial infarction” and “heart attack”) that are lexically completely different but refer to the same concept. Our mutual information‑based feature selection relies on word frequency statistics and struggles to identify such synonymy, potentially treating “myocardial infarction” and “heart attack” as two independent features, resulting in insufficient redundancy retention or erroneous elimination. Meanwhile, although contrastive learning features can capture a certain degree of semantic similarity, they are limited by the domain adaptability of the pre‑trained language model – general‑purpose BERT has limited representational power for biomedical texts.
- Tweet’s NMI not reaching first place may be related to the continuity of sentiment expression. Sentiment data exhibit a gradual transition from positive to negative, while the clustering task requires discretizing the continuous space into several categories. Our framework, by maximizing mutual information between positive pairs, inherently tends to form clusters with clear boundaries, which conflicts with the continuous nature of sentiment data. For such data, the discretization process inherent to hard clustering inevitably causes information loss, explaining the relatively lower NMI.

### 5.3 Comparison with related literature

We systematically compare the proposed framework with four representative types of methods, clarifying their respective characteristics and applicable scenarios from a theoretical positioning perspective.

- Comparison with traditional feature models. BOW (2015) and TF‑IDF (2015), as classic feature representation methods, are based on the core assumption that word frequency statistics suffice to capture document semantics. The essential difference between our method and these approaches lies in whether dependencies among features are modeled – BOW/TF‑IDF assume that feature dimensions are independent of each other, whereas we explicitly model redundancy relationships among features via mutual information and perform selection on this basis. Experiments show that in high‑dimensional sparse scenarios, feature selection that accounts for feature dependencies yields a more compact and effective feature subset.
- Comparison with probabilistic graphical models. GSDPMM (2016), as a variant of the Dirichlet process mixture model, uses Bayesian nonparametric methods to automatically infer the number of classes. Its advantage lies in not requiring a prespecified K value, but on short texts it is limited by the bag‑of‑words assumption and struggles to capture deep semantics. Our method introduces semantic abstraction through contrastive learning, overcoming the limitations of the bag‑of‑words model. The two approaches are theoretically complementary – combining contrastive learning representations with Bayesian nonparametric models is a promising direction for future research.
- Comparison with deep neural network models. STC^2^-LPI (2017) and Self‑Train (2019) represent two deep clustering paradigms: graph Laplacian‑based objective optimization and self‑training‑based iterative refinement. BERT (2018), as a pre‑trained language model, provides semantic priors through large‑scale corpus pre‑training. The essential difference between our framework and these methods lies in the explicit application of mutual information – although these methods implicitly exploit some form of dependency, they do not use mutual information as a core optimization objective. Our paper explicitly maximizes/minimizes mutual information at both the feature selection and contrastive learning levels, ensuring that information‑theoretic principles pervade the entire framework.
- Comparison with contrastive learning models. SCCL (2021), an early exploration combining contrastive learning and clustering, performs clustering directly after enhancing representations via contrastive learning, where the contrastive objective typically includes both positive sample attraction and negative sample repulsion. RSTC (2023) introduces a robustness mechanism on this basis. MIST (2024) models from the perspective of mutual information maximization, optimizing mutual information at both the token level and the sequence level. POTA (2025) uses optimal transport to generate pseudo‑labels, achieving more reliable clustering guidance. The core difference between our method and the above contrastive learning models is that we use only positive‑pair mutual information maximization without introducing negative sample enhancement. The theoretical motivation for this design choice is that in unsupervised clustering scenarios, true negative samples are difficult to define – texts that are semantically similar but belong to different classes may be mistakenly treated as negative samples, causing the optimization objective to deviate. By focusing on maximizing mutual information for positive pairs, our method avoids this risk but may also sacrifice some discriminative information. Experimental results indicate that this trade‑off is effective on most datasets (achieving the highest rank on 5 out of 8 metrics), but performs slightly worse on datasets requiring fine‑grained distinctions (Stackoverflow, Tweet) compared to models that include negative sampling mechanisms. Compared with MIST, both methods use mutual information as a core theoretical tool, but at different levels of application: MIST directly maximizes the mutual information between representations and inputs, whereas we minimize mutual information among features at the feature selection level to reduce redundancy and maximize mutual information for positive pairs at the contrastive learning level to enhance representations. This “bidirectional application” makes more comprehensive use of mutual information.
- Comparison with large language models. ClusterLLM (2023) represents a paradigm for applying large language models to clustering tasks: generating guidance signals via an LLM to assist a small embedding model. Its advantage lies in leveraging the LLM’s extensive world knowledge, but it incurs substantial computational overhead and is limited by the LLM’s context window. Our framework, by mining semantic structure from the data itself via mutual information, achieves comparable performance while reducing computational cost by more than an order of magnitude. This comparison reveals a trade‑off in the source of knowledge – external knowledge (LLMs) versus data‑intrinsic structure (mutual information) – each with its own strengths and suitable scenarios.

### 5.4 Research implications

The experimental findings of this study offer several important implications for the field of short text clustering.

- Implication 1: Mutual information can serve as a unified theoretical framework connecting feature selection and representation learning. This paper demonstrates the dual application of mutual information at the levels of feature selection (reducing dimensional redundancy) and contrastive learning (maximizing mutual information of positive pairs). This design is not coincidental – as an information‑theoretic measure of dependence between variables, mutual information is naturally suited to describing various relationships such as “feature‑feature” and “representation‑representation”. Using mutual information as the unifying theoretical language throughout the framework facilitates theoretical consistency across modules and provides an extensible methodological foundation for subsequent research.
- Implication 2: Feature selection and representation learning are complementary; their combined use outperforms either alone. Experiments show that using mutual information‑based feature selection alone or contrastive learning‑based representation enhancement alone yields inferior performance compared to their fusion. This finding suggests that explicit features (interpretable lexical dimensions) and implicit features (abstract semantic representations) each have value; an ideal representation learning framework should consider both rather than completely discarding the original feature space.
- Implication 3: KAN networks have the potential to replace MLPs in structured feature scenarios. Experimental results show that, given fused features, KAN exhibits more stable clustering performance compared to MLP. This is consistent with KAN’s theoretical properties – the local adaptability of spline functions enables finer‑grained decoupled processing of feature dimensions. For research scenarios where feature dimensions are relatively independent and sample sizes are limited, KAN is a promising alternative to MLP.
- Implication 4: A contrastive learning strategy that focuses solely on maximizing positive‑pair mutual information, while avoiding negative sample noise, may also sacrifice some discriminative information. Our contrastive learning module only attends to maximizing the information content of positive pairs and does not involve negative sample enhancement. The advantage of this design is that it avoids the noise that negative sample construction might introduce (especially in unsupervised scenarios, where the authenticity of negative samples is difficult to guarantee). However, the cost is that the model may not adequately learn fine‑grained decision boundaries. The relatively lower performance on Stackoverflow and Tweet may be related to this – these two datasets require fine‑grained distinctions of technical topics and sentiment intensities, respectively, and relying solely on positive‑pair consistency makes it difficult to capture these subtle differences.

## 6 Conclusion

This paper addresses the issues of semantic sparsity and feature redundancy in short text clustering by proposing a three-stage clustering framework based on the dual application of mutual information. The framework first employs mutual information to analyze dimensional correlations among instance features, reducing redundancy to form a low‑redundancy feature representation. Second, it applies mutual information theory to maximize the information content of positive instance pairs while incorporating a negative sample enhancement learning strategy to extract contrastive learning features. Finally, the two complementary types of features are jointly fed into a KAN network for clustering. Experimental results on eight benchmark datasets, including AG News, StackOverflow, Biomedical, SearchSnippets, and Tweet, demonstrate the significant advantages of the proposed framework in clustering performance: except for the NMI on StackOverflow, the ACC on Biomedical, and the NMI on Tweet, the framework achieves the best ACC and NMI on all remaining datasets. Furthermore, we applied the proposed model to cluster Weibo data crawled using the keyword “technology” from January 27 to February 28, 2025. The clustering results validate the effectiveness of the proposed model. Future work will focus on the following directions: improving clustering performance on imbalanced data via weighted mutual information; extending the model to multilingual text clustering with cross-lingual embedding techniques; and reducing the number of model parameters while enhancing real-time inference efficiency. These efforts aim to further improve the generalization capability and practicality of the proposed framework.

## Supporting information

S1 File AgNews dataset. (ZIP)

S2 File StackOverflow dataset. (ZIP)

S3 File Biomedical dataset. (ZIP)

S4 File SearchSnippets dataset. (ZIP)

S5 File Tweet dataset. (ZIP)
