# Supplementary material for: Contrastive learning with mutual information enhancement and negative sample augmentation combined with KAN for text clustering
Source: PLoS One. 2026 Jul 30;21(7):e0354718. doi: 10.1371/journal.pone.0354718 (PMC13422851; doi:10.1371/journal.pone.0354718)
Supplement: Attachment — Submitted filename: response letter (major revison).docx [file pone.0354718.s007.docx]

**Response Letter**

Dear Editor and Reviewers,

We are grateful for the insightful feedback and valuable suggestions provided by you and the esteemed reviewers on our manuscript (No.: PONE-D-25-55666; Title: Contrastive Learning with Mutual Information Enhancement and Negative Sample Augmentation Combined with KAN for Text Clustering; Authors: Yuanmin Zhang, Hao Li,Chunzhi Xie,Yong Huang, Zhenyi Wu,Yanjun Li) submitted to PLOS One. Following a comprehensive review of the comments received, we have diligently made all the recommended revisions to our manuscript. All modifications in the manuscript are marked with red font for reviewer-related revisions and blue font for improvements in organization and language, to ensure clarity and traceability.

Please do not hesitate to contact us should you have any further questions or require additional details regarding our revised submission. We kindly request acknowledgment of this revision's receipt and look forward to your response.

Sincerely,

Chunzhi Xie

On behalf of all co-authors

mail: xcz\_xihua@sina.com

Address: School of Computer and Software Engineering, Xihua University, Chengdu 610039, China

**Response to reviewers**

*Reviewer #1:*

*The manuscript presents an experimental evaluation and a technically sophisticated framework. The topic is relevant, and the proposed approach shows potential. However, several issues limit the scientific rigor of the contribution in its current form. Addressing the concerns outlined below is necessary to strengthen the manuscript and improve its credibility.*

**Response：**

We sincerely appreciate you for the thorough, professional, and insightful evaluation of our manuscript. We are grateful for your recognition of the relevance of our topic, the technical soundness of the proposed framework, and the potential of our experimental evaluation. We fully acknowledge that the scientific rigor and credibility of the manuscript need to be further strengthened. In response to all your constructive comments and suggestions, we have carefully revised the manuscript point-by-point, supplemented sufficient literature support, adjusted overly assertive expressions, clarified technical definitions and assumptions, added a dedicated Discussion section, improved the analysis of experimental results and visualizations, and polished the overall presentation. We believe these revisions have greatly improved the academic quality, rigor, and readability of the manuscript. Thank you again for your valuable and constructive comments.

*1. Insufficient Bibliographic Support for Conceptual and Methodological Claims
A major concern throughout the manuscript is the lack of adequate bibliographic support for several conceptual and methodological assertions. This issue is particularly evident in:
• Statements regarding the limitations of contrastive learning in linear embedding spaces;
• General discussions on feature redundancy, anisotropy in embedding spaces, and the curse of dimensionality, which are treated as well-known phenomena but are often not supported by foundational or recent studies;
• Assertions that specific mechanisms, such as mutual information maximization, feature-level redundancy reduction, or KAN-based transformations, effectively or significantly mitigate issues, without references to prior theoretical or empirical work motivating these expectations.*

**Response：**

We sincerely appreciate your valuable and constructive comment. In the revised manuscript, we have comprehensively addressed this issue by supplementing authoritative, foundational, and recent references for all relevant assertions, specifically:

- For statements regarding the limitations of contrastive learning in linear embedding spaces, we have cited classic and cutting-edge studies that systematically discuss the constraints of contrastive learning when applied to linear embedding scenarios, clarifying the theoretical basis for our related claims.
- For general discussions on feature redundancy, embedding space anisotropy, and the curse of dimensionality, we have added foundational works that define and validate these phenomena, as well as recent studies that extend related research, ensuring these concepts are supported by sufficient academic evidence rather than being treated as self-evident.
- For assertions about the effectiveness of specific mechanisms (mutual information maximization, feature-level redundancy reduction, KAN-based transformations), we have referenced prior theoretical studies that establish the rationality of these mechanisms and empirical works that verify their effectiveness in similar tasks, clearly linking our approach to existing academic achievements.

All added references are carefully selected to be representative and relevant, and we have ensured consistent formatting between in-text citations and the reference list. These revisions significantly strengthen the academic foundation of the manuscript and enhance the persuasiveness of our conceptual and methodological claims. Thank you again for your patient and meticulous guidance on our work.

*2. Overly Assertive Language and Strength of Claims
The manuscript frequently employs strong and absolute phrasing (e.g., “effectively solves”, “unequivocally outperforms”, “state-of-the-art performance”). In several instances, these claims are not proportionally supported by statistical significance analysis, controlled comparisons, or evaluations across a sufficiently broad range of experimental conditions. Stronger claims should be reinforced with additional quantitative evidence or references to prior studies reporting similar outcomes.*

**Response：**

We sincerely appreciate your insightful and constructive comment. In the revised manuscript, we have comprehensively revised the language throughout the text to address this issue:

We have replaced all strong and absolute phrases (e.g., “effectively solves”, “unequivocally outperforms”, “state-of-the-art performance”) with modest, academically appropriate expressions (e.g., “can effectively alleviate”, “achieves competitive performance compared with”, “demonstrates promising performance relative to existing methods”), ensuring our claims are prudent and consistent with academic norms.

For claims that lacked sufficient support, we have supplemented additional quantitative evidence, including statistical significance analysis (e.g., t-tests) to verify the reliability of experimental results, expanded controlled comparisons with baseline methods, and extended evaluations across a broader range of experimental conditions to enhance the persuasiveness of our conclusions.

For stronger claims regarding the effectiveness of our proposed method, we have referenced prior studies that report similar outcomes, establishing a clear link between our findings and existing academic research to further reinforce the rationality of our assertions.

We have carefully checked the entire manuscript to ensure no overly assertive language remains, and all claims are now proportionally supported by evidence or relevant references. These revisions significantly improve the scientific rigor and credibility of the manuscript. Thank you again for your patient and meticulous guidance on our work.

*3. Implicit Technical Assumptions and Lack of Definitions
Several technical assumptions are implicitly treated as common knowledge, despite not being universally established. Examples include:
• The relationship between mutual information magnitude and semantic dependency at the feature level;
• The assumed superiority of learnable spline-based activations over linear transformations in high-dimensional clustering contexts;
• The generalization capability of KAN architectures in mitigating the curse of dimensionality.
While these assumptions may be plausible, they should be either supported by references or clearly framed as hypotheses validated empirically in this study. In addition, several technical terms (e.g., training steps, topic popularity,* *false negative samples) are introduced without definition or are defined only after being used. Definitions should be provided at first occurrence.*

**Response：**

We sincerely appreciate your careful and constructive comment. In the revised manuscript, we have made thorough revisions as follows:

- We have explicitly stated and justified all previously implicit technical assumptions. For the relationship between mutual information and feature-level semantic dependency, the superiority of learnable spline-based activations over linear transformations in high-dimensional clustering, and the generalization ability of KAN in alleviating the curse of dimensionality, we have either added supporting references or clearly described them as hypotheses that are verified through empirical experiments in this work.
- We have checked all technical terms and provided clear definitions at their first occurrence, including training steps, topic popularity, false negative samples, and other key expressions.

These changes ensure that all assumptions are well-supported and all terminologies are clearly explained, which greatly improves the logical consistency and readability of the manuscript. Thank you again for your patient and meticulous guidance on our work.

*4. Limited Engagement with Alternative or Conflicting Viewpoints
Although the Related Work section is extensive, it primarily emphasizes literature that supports the proposed approach. The manuscript would benefit from a more balanced discussion that also considers:
• Alternative paradigms addressing redundancy or anisotropy in embedding spaces;
• Potential drawbacks or trade-offs associated with mutual information–based regularization;
• Known limitations of contrastive learning frameworks that may also apply to the proposed method.*

**Response：**

We sincerely appreciate your professional and constructive comment. In the revised manuscript, we have significantly expanded and improved the Related Work section to provide a more impartial review:

We have added a systematic discussion of alternative paradigms for addressing embedding space redundancy and anisotropy, comparing their ideas, strengths, and limitations with our method.

We have explicitly analyzed the potential drawbacks and trade-offs associated with mutual information–based regularization, providing a more comprehensive view of its mechanism.

We have also supplemented a discussion on the inherent limitations of contrastive learning frameworks and have objectively explained which challenges may also apply to our proposed model.

These revisions make the literature review more comprehensive, balanced, and persuasive, and better highlight the positioning and contributions of this work. Thank you again for your patient and meticulous guidance on our work.

*5. Absence of a Dedicated Discussion Section
While the Results section includes localized interpretations of experimental outcomes, the manuscript lacks a dedicated Discussion section. This limits higher-level integration of findings and weakens the connection between empirical results and the broader literature. Although not strictly mandatory, the inclusion of a Discussion section is strongly recommended given the methodological complexity of the proposed framework.*

**Response：**

We sincerely appreciate your valuable and professional suggestion. In the revised manuscript, we have added a new, independent Discussion section. In this section, we systematically summarize and interpret the key experimental findings, analyze the effectiveness and rationality of the proposed framework, discuss the insights obtained from the experiments, compare our results with relevant studies in the literature, and objectively address the limitations and potential future improvements of the proposed method. This addition greatly enhances the logical completeness, interpretability, and academic rigor of the manuscript. Thank you again for your patient and meticulous guidance on our work.

*6. Dependence on Hyperparameters and Design Choices
The performance of the proposed method appears sensitive to hyperparameter settings (e.g., λ values, batch size, number of clusters). While hyperparameter tuning is mentioned, several architectural and experimental choices, such as encoder selection, KAN configuration, and loss weighting, are not sufficiently justified through references or systematic empirical analysis. Where possible, ablation studies or references to prior work should be used to justify key design decisions.*

**Response：**

We sincerely appreciate your insightful and constructive comment. In the revised manuscript, we have comprehensively supplemented the justification and analysis for all critical hyperparameters and architectural decisions:

- We have added systematic sensitivity analysis for important hyperparameters including λ, batch size, and number of clusters, clearly demonstrating their influence on model performance and the rationale behind our final selection.
- We have supplemented detailed justification for encoder selection, KAN structure configuration, and loss function weighting strategies, supported by relevant references and additional ablation experiments to verify the rationality and effectiveness of these design choices.
- We have clearly explained the motivation and experimental evidence for each key design decision to ensure transparency and sufficiency of justification.

These revisions significantly strengthen the reliability and persuasiveness of the experimental design and model architecture. Thank you again for your patient and meticulous guidance on our work.

*7. Interpretation of Visual Analyses
Visualizations based on techniques such as t-SNE and temporal topic popularity trends are informative, but their interpretations are occasionally too strong. Given the qualitative and parameter-sensitive nature of these methods, their illustrative role should be explicitly acknowledged, and they should not be treated as direct quantitative evidence of superiority.* *In particular, the concept of “topic popularity” requires a clear quantitative definition. The scale used in the corresponding figures (e.g., 0–1000) should be explicitly explained in the text.
Specific Comments
• Consider adding a paragraph at the end of the Introduction outlining the structure of the manuscript.
• Several paragraphs are overly long. Shorter paragraphs (ideally no more than 10–12 lines) would improve readability.
• Figures and tables should be placed closer to their first citation in the text and should be explicitly referenced before appearing.
• Some acronyms and abbreviations are not defined at first occurrence. Please ensure all abbreviations are spelled out in full upon first use.*

**Response：**

We sincerely appreciate your careful and detailed comments. In the revised manuscript, we have addressed all points as follows:

- We have revised the interpretation of t-SNE and temporal topic popularity visualizations. We explicitly acknowledge that these figures serve a qualitative and illustrative purpose rather than providing direct quantitative evidence of performance superiority. We have also added a clear quantitative definition of “topic popularity” and explicitly explained the scale range (e.g., 0–1000) in the text and figure captions.
- We have added a paragraph at the end of the Introduction to clearly outline the overall structure of the manuscript.
- We have split all overly long paragraphs into shorter ones (generally within 10–12 lines) to improve readability.
- We have adjusted the positions of all figures and tables to ensure they appear immediately after their first citation in the text and are properly referenced before they appear.
- We have checked all acronyms and abbreviations carefully and ensured that each abbreviation is defined in full at its first occurrence throughout the manuscript.

We believe these revisions have greatly improved the standardization, clarity, and readability of the manuscript. Thank you again for your patient and meticulous guidance on our work.

*Abstract
• Lines 13–14: Consider removing the specific days and stating the time frame more generally, for example as “during January and February 2025”.*

**Response：**

We thank you for the suggestion. We have revised the text to replace specific days with the general time frame “during January and February 2025” in Lines 13–14. Thank you again for your patient and meticulous guidance on our work.

*Introduction
• Lines 18–20: Additional references are required to support the claims made in this passage.*

**Response：**

Thank you for the constructive comment. We have carefully reviewed Lines 18–20 and added several relevant and authoritative references to fully support the claims and statements in this passage. The corresponding citations have been inserted into the text and listed in the reference section accordingly. Thank you again for your patient and meticulous guidance on our work.

*•Lines 25–32: Several key concepts (e.g., contrastive learning, anisotropy, and linear alignment strategies) are introduced without sufficient explanation. Brief contextualization would improve clarity and accessibility for readers less familiar with representation learning.*

**Response：**

Thank you for this valuable suggestion. We have carefully revised Lines 25–32 and added brief contextual explanations for the key concepts, including contrastive learning, anisotropy, and linear alignment strategies. These additional descriptions will improve clarity and make the work more accessible to readers who are less familiar with representation learning. The corresponding revisions have been made in the manuscript.

*Line 33: Please spell out the acronym “SCCL” at its first occurrence in the text. This should be consistently verified for all acronyms throughout the manuscript.*

**Response：**

Thank you for your careful check. We have spelled out the full name of the acronym “SCCL” at its first occurrence in the manuscript. In addition, we have carefully reviewed and verified all other acronyms throughout the paper to ensure consistency in their first-time definition. Thank you again for your patient and meticulous guidance on our work.

*•Line 76: Consider replacing “pitting” with “evaluating”.*

**Response：**

Thank you for the careful suggestion. We have replaced “pitting” with “evaluating” at Line 76 as recommended, and have double-checked the wording throughout the manuscript for accuracy. Thank you again for your patient and meticulous guidance on our work.

*•**Certain technical terms and claims (e.g., “**robust text representations”) could be phrased more precisely or briefly.*

**Response：**

Thank you for this constructive comment. We have carefully revised the technical terms and claims (e.g., “robust text representations”) throughout the manuscript to make the expressions more precise and concise. Thank you again for your patient and meticulous guidance on our work.

*•Simplifying long sentences, particularly in the paragraph introducing the proposed method, would improve accessibility.*

**Response：**

Thank you for this helpful suggestion. We have simplified the long sentences throughout the manuscript, especially in the paragraph introducing the proposed method, to improve readability and accessibility. Thank you again for your patient and meticulous guidance on our work.

*2. Related Work
• Line 79: Consider replacing “Related work” with “Related Work” for consistency.*

**Response：**

We appreciate your careful check. We have revised “Related work” to “Related Work” at Line 79 for consistency throughout the manuscript. Thank you again for your patient and meticulous guidance on our work.

*• Line 88: Consider using “CNNs”.
• Line 158: Consider using “NLP”.*

**Response：**

Thank you for your careful suggestions. We have used “CNNs” at Line 88 and “NLP” at Line 158 as recommended. Thank you again for your patient and meticulous guidance on our work.

*• Line 161: Please correct the missing space in “generation.For”.*

**Response：**

Thank you for your careful check. We have corrected the missing space in “generation.For” at Line 161 to “generation. For”, and have reviewed the manuscript for similar formatting issues.

*• Line 163: The citation “Pairsupcon” is not numbered and does not appear in the References list.*

**Response：**

Thank you for pointing out this issue. We have corrected the citation “Pairsupcon” at Line 163 by adding the correct reference number and including the full reference in the References list. We have also double-checked all citations and references for consistency throughout the manuscript. Thank you again for your patient and meticulous guidance on our work.

*• Splitting long paragraphs and consolidating overlapping descriptions would improve readability.*

**Response：**

Thank you for this constructive suggestion. We have split the long paragraphs and consolidated the overlapping descriptions throughout the manuscript, which has greatly improved the readability of the paper. Thank you again for your patient and meticulous guidance on our work.

*3. Proposed Model
•* *Line 196: Consider replacing “dubbed” with “termed”.*

**Response：**

Line 196:replaced. Thank you for your patient and meticulous guidance on our work.

*• Line 196: The acronym “MIKAN” is introduced without definition. Please spell out its full meaning at first mention.*

**Response：**

Thank you for pointing out this oversight. We have spelled out the full meaning of the acronym “MIKAN” at its first mention in Line 196, ensuring clarity for all readers. We have also double-checked to confirm consistency with other acronyms throughout the manuscript. Thank you again for your patient and meticulous guidance on our work.

*• Line 201: Consider replacing “ameliorate” with “mitigate”.
• Lines 207–208: Consider using only “contrastive learning” to avoid redundancy.
• Line 208: Consider replacing “Evidently” with “Consequently” to soften the assertiveness of the statement.
• Line 210: Consider rephrasing as “achieving more robust …” for improved clarity.
• Line 221: Consider replacing “**ameliorate” with “**mitigate” for consistency.*

**Response：**

Thank you for these detailed and constructive suggestions. We have carefully revised the manuscript as recommended:

- Replaced “ameliorate” with “mitigate” at Line 201;
- Used only “contrastive learning” in Lines 207–208 to avoid redundancy;
- Replaced “Evidently” with “Consequently” at Line 208 to make the statement less assertive;
- Replaced Line 210 as “achieving more robust …” for improved clarity;
- Replaced “ameliorate” with “mitigate” at Line 221 for consistency.All revisions have been implemented in the manuscript.

Thank you again for your patient and meticulous guidance on our work.

*• Some statements appear overly assertive (e.g., claims that certain mechanisms “fully capture” specific properties). Softening these formulations would help maintain a cautious scientific tone.*

**Response：**

We sincerely appreciate your careful and constructive comment. In the revised version, we have carefully revised all such strong statements throughout the text. We have replaced overly assertive phrases with more modest, rigorous, and academically appropriate formulations (e.g., “effectively capture”, “better model”, “reasonably characterize”), while preserving the core meaning. These revisions ensure the manuscript maintains a prudent, objective, and scientific writing style. Thank you again for your patient and meticulous guidance on our work.

*Certain theoretical statements, such as those regarding the flexibility of KANs due to learnable activation functions, would benefit from supporting references.*

**Response：**

We sincerely appreciate your careful and constructive comment. In the revised manuscript, we have added relevant and authoritative references that justify the flexibility and representation power of KANs enabled by learnable activation functions. These citations provide solid theoretical support for our related claims and improve the scientific rigor of the manuscript. Thank you again for your patient and meticulous guidance on our work.

*• While the overall workflow is clearly presented, briefly clarifying where and how mutual information optimization and feature-level redundancy reduction are implemented within the architecture would further strengthen clarity and reproducibility.*

**Response：**

We sincerely appreciate your constructive and valuable comment. In the revised manuscript, we have added brief but clear explanations to explicitly illustrate where and how mutual information optimization and feature-level redundancy reduction are integrated and implemented in the overall workflow and network architecture. We have also refined the description of the corresponding computational modules to ensure the implementation details are sufficiently transparent and reproducible.

These revisions make the architectural design more understandable and enhance the repeatability of the proposed framework. Thank you again for your patient and meticulous guidance on our work.

*3.1 MIKAN*
*• Lines 224–226: This passage appears redundant and may be removed, as the same definition is already provided in Lines 194–196.
• Figure 1: Consider using the label “Pre-trained Encoder” for consistency. In addition, the same shade of green is used at two different stages, which may unintentionally suggest that these elements correspond to the same dataset or representation.*

**Response：**

We sincerely appreciate your careful and detailed comments on Section 3.1 and Figure We have addressed both points thoroughly in the revised manuscript.

- Regarding Lines 224–226: We agree that this passage is redundant because the same definition has already been given in Lines 194–196. We have removed these redundant sentences to improve conciseness and readability.
- Regarding Figure 1: We have revised the label to “Pre-trained encoder” to ensure consistency with terminology used elsewhere in the manuscript. We have also adjusted the color scheme so that different stages no longer share the same shade of green, avoiding any misleading implication that they correspond to the same dataset or feature representation.

These changes enhance the clarity, consistency, and presentation quality of the manuscript. Thank you again for your patient and meticulous guidance on our work.

*3.2 Mutual Information …
• The definition of entropy may need revision. Entropy is typically defined for a random variable rather than a specific realization. Accordingly, the notation should need to be changed from H(x) to H(X) (Formula 1), with the summation taken over the support of the random variable.*

**Response：**

We sincerely appreciate your careful and professional comment on the definition of entropy and related notation. In the revised manuscript, we have corrected Formula 1 by changing the notation from H(x) to H(X) to reflect that entropy is defined for a random variable X. We have also revised the corresponding description to emphasize that the summation is performed over the support of the random variable. These corrections ensure the theoretical formulation is rigorous and consistent with standard information theory. Thank you again for your patient and meticulous guidance on our work.

*3.3 Feature-level Redundancy …*

*Line 342 and 380: Consider removing the word “**Evidently”.
• Line 369:* *Please ensure consistent use of quotation marks for ‘easy’.
• The notation appears to be inconsistent throughout the section: batch size is alternately denoted as “b” and “B”, and feature dimensionality as “d” and “D”. Adopting a single notation would improve clarity.
•The normalization of the feature matrix may require clarification. Dividing by the sum of squared feature values does not correspond to standard L2 normalization. If the intention is to project features onto the unit hypersphere, normalization would typically involve the square root of the sum of squared elements, which directly affects feature geometry and similarity computation.
• The probabilistic interpretation of the self-correlation matrix Mself appears to be unclear. While treating a normalized affinity matrix as an approximation of a joint probability distribution can be acceptable in practice, this assumption should be explicitly stated. As currently written, P(Ei, Ej) seems to be treated as a true probability distribution without explicitly satisfying the axioms of probability.
• The definitions of the marginal probability distributions P(Ei) and P(Ej) appear to be ambiguously formatted and do not clearly specify the summation indices. If this interpretation is correct, providing explicit formulations would improve clarity and reproducibility.
• The role of the balancing parameter β is only briefly described. A clearer theoretical interpretation of β (e.g., as smoothing or scaling parameter) would strengthen the proposed loss function.*

**Response：**

We sincerely appreciate you for the careful and thorough comments on Section 3.3. We have addressed every concern in detail as follows:

- Word usage: We have removed the word “Evidently” at Lines 342 and 380 to maintain a cautious and objective scientific tone.
- Quotation marks: We have standardized the quotation marks for the term ‘easy’ to ensure consistent formatting throughout the manuscript.
- Notation consistency: We have unified all notations in this section: batch size is denoted consistently as B, and feature dimensionality as D, to avoid ambiguity.
- Feature normalization: We have clarified the exact formulation of feature matrix normalization. We have revised the description to explicitly distinguish between sum-of-squares scaling and standard L₂ normalization (which uses the square root of the sum of squared elements), and we have clearly explained how our normalization projects features onto the unit hypersphere, ensuring consistency with standard feature geometry and similarity computation.
- Probabilistic interpretation of self-correlation matrix Mₛₑₗբ: We have explicitly stated the assumption that the normalized affinity matrix is used as an approximation of a joint probability distribution. We have clarified the interpretation of P(Eᵢ, Eⱼ) and emphasized that it represents an empirical probabilistic approximation rather than a strictly normalized probability distribution, making the theoretical foundation more transparent.
- Marginal distributions P(Eᵢ) and P(Eⱼ): We have revised and clearly formatted the definitions of the marginal probability distributions, with explicit summation indices and unambiguous mathematical expressions to improve readability and reproducibility.
- Interpretation of balancing parameter β: We have added a clearer theoretical interpretation of β, explaining its role as a smoothing and scaling parameter that balances the contribution of different components in the loss function. This strengthens the motivation and theoretical soundness of the proposed loss.

All revisions improve the precision, consistency, and rigor of the methodology. Thank you again for your thorough review and patient guidance.

*3.4 Negative Sample ...
• Equations (13–16): Please correct the typo “Achor” to “Anchor” throughout the equations.
The notation used throughout the section appears to be inconsistent. The total number of samples is denoted interchangeably as “B” and “N”, while additional symbols such as “M”, “L”, and “T” are introduced without clear and unified definitions.
The loss function associated with hard negative samples (Eq. 14) appears difficult to interpret, as it combines regular negatives, hard negatives, and weighting terms. The interaction between the amplified repulsion term and the standard contrastive denominator may require further clarification.
 While the attraction mechanism proposed for false negative samples is conceptually reasonable, the formulation of the attraction weight and the corresponding loss functions (Eq. 15 and 16) may benefit from clearer theoretical justification. Although an analogy to attention mechanisms is mentioned, this connection does not appear to be formally established.
The role of the proposed loss LIHSF within the overall optimization framework is not explicitly stated. The authors should clarify whether this loss functions as a primary objective or as an auxiliary regularization term, and how it interacts with the other loss components of the model.*

**Response：**

We sincerely appreciate you for the careful and insightful comments on Section 3.4. We have addressed all concerns thoroughly as follows:

- Typo correction: We have corrected the typo “Achor” to “Anchor” in Equations (13–16) and throughout the entire section.
- Notation consistency: We have unified all inconsistent notations in Section 3.4. The total number of samples is consistently denoted as N, and all newly introduced symbols including M, L, and T are clearly defined at their first occurrence to avoid ambiguity.
- Interpretability of hard negative loss (Eq. 14): We have added a clearer explanation of the loss function for hard negative samples, explicitly clarifying how regular negatives, hard negatives, and weighting terms are combined, and how the amplified repulsion term interacts with the standard contrastive denominator.
- Theoretical justification for attraction weight and loss (Eqs. 15–16): We have strengthened the theoretical justification for the attraction mechanism on false negative samples and the corresponding loss formulations. We have formally established the connection to attention mechanisms and provided clearer motivation to improve the rationality and interpretability of the design.
- Role of loss LIHSF: We have explicitly clarified the role of LIHSF in the overall optimization framework, specifying whether it serves as a main objective or an auxiliary regularization term, and explaining how it interacts with other loss components to guide model optimization.

These revisions significantly improve the clarity, consistency, and theoretical rigor of Section 3.4. Thank you again for your patient and meticulous guidance on our work.

*3.5 Kolmogorov–Arnold Networks …*

*• Lines 420–422: The authors are encouraged to add an appropriate reference.
• Lines 453–454: Please consider adding a reference.
• Line 461: Please ensure consistent use of quotation marks for “curse of dimensionality”.
• While the mathematical formulation of the KAN forward propagation and activation functions appears generally correct, claims regarding model efficiency may require clarification. The assertion that KANs substantially reduce the number of parameters compared to fully connected layers is not sufficiently justified.
 The statement that the proposed approach alleviates the “curse of dimensionality” appears somewhat strong. While KANs can improve function approximation and representation expressiveness in high-dimensional spaces, a more cautious formulation indicating partial mitigation of high-dimensional effects may be more appropriate.
The computational complexity and scalability of the KAN clustering module are not discussed. Given the use of spline functions and the configuration S = Din, a brief discussion of computational and memory implications would strengthen the section.
•While the clustering loss based on pseudo-labels and cross-view consistency is conceptually appropriate, key operational details remain unclear, such as whether pseudo-labels are updated iteratively, whether gradient flow is stopped during pseudo-label generation, and how training stability is ensured.
The rationale for producing three separate score matrices (Y’, Y’1, and Y’2) and their precise role within the overall optimization process should be described more explicitly.*
**Response：**

We sincerely appreciate you for the careful and constructive comments on Section 3.5. We fully agree that proper citations, consistent notation, cautious scientific claims, clear computational complexity analysis, and detailed implementation descriptions are essential to improve the rigor, clarity, and reproducibility of the manuscript. We have carefully addressed all of the concerns as follows:

- Added references for Lines 420–422 and Lines 453–454:

We have added appropriate and authoritative references to support the statements in Lines 420–422 and Lines 453–454, strengthening the theoretical background and literature basis of the KAN-related descriptions.

- Consistent quotation marks for “curse of dimensionality”:

We have standardized the quotation marks for the phrase “curse of dimensionality” at Line 461 to ensure consistent formatting throughout the manuscript.

- Clarification of model efficiency and parameter reduction claims:

We have revised and clarified the claims regarding the efficiency of KANs. We now provide clearer justification for how KANs reduce the number of parameters compared with standard fully connected layers, supported by theoretical analysis and relevant references, to ensure the statement is well grounded.

- Softened claims about alleviating the “curse of dimensionality”:

We have revised the strong statement about fully mitigating the “curse of dimensionality” to a more cautious and accurate formulation. We now state that the proposed approach partially mitigates high-dimensional challenges and improves representation expressiveness in high-dimensional spaces, which is more scientifically rigorous.

- Added discussion of computational complexity and scalability:

We have supplemented a brief analysis of the computational complexity and memory usage of the KAN clustering module, especially regarding the use of spline functions and the setting S=Din. This additional discussion improves the transparency and completeness of the module design.

- Clarified implementation details of pseudo-label clustering loss:

We have explicitly clarified key operational details, including whether pseudo-labels are updated iteratively, whether gradients are detached during pseudo-label generation, and how training stability is maintained. These details significantly improve the reproducibility of the method.

- Explicit explanation of three score matrices Y′, Y1′, Y2′:

We have added a clear description of the motivation for generating three distinct score matrices, as well as their specific roles and interactions within the overall optimization framework. Their contributions to cross-view consistency and clustering guidance are now explicitly explained.

All revisions have improved the accuracy, rigor, and readability of Section 3.5. Thank you again for your patient and meticulous guidance on our work.

*4.2 Experimental Dataset
• While the definition of the dataset statistics (N, C, A, and R) is clear, the relevance of the imbalance ratio “R” could be more explicitly discussed.*

**Response：**

We sincerely appreciate your constructive comment. In the revised manuscript, we have added a clear explanation of the significance and role of the imbalance ratio R in characterizing the degree of class imbalance within the datasets. We have clarified how imbalance affects clustering performance and why R serves as an important metric for evaluating the robustness of the proposed method under imbalanced distributions. This additional explanation strengthens the motivation and interpretability of the experimental setup. Thank you again for your patient and meticulous guidance on our work.

*4.3 Experimental Environment and Parameters
• Line 544: Please ensure consistent quotation formatting for the model name.
• The reported average runtime per dataset (18 h) should be clarified, for example by indicating whether it includes data preprocessing steps.
• When referring to the use of t-SNE for visualization, please clarify whether default parameters were used or specify the key parameter settings adopted.*

**Response：**

We sincerely appreciate your careful and constructive comments on Section 4.3. We have addressed all of the points thoroughly in the revised manuscript as follows:

- Consistent quotation formatting: We have standardized the quotation marks for the model name at Line 544 to ensure consistent formatting throughout the manuscript.
- Clarification of average runtime: We have clarified that the reported average runtime of 18 hours per dataset refers only to model training and inference, and we have explicitly stated whether data preprocessing is included. This makes the experimental reporting more transparent and reproducible.
- Clarification of t-SNE parameters: When describing t-SNE for visualization, we have explicitly specified whether default parameters were used or listed the key parameter settings we adopted, ensuring full transparency of the visualization setup.

These revisions improve the consistency, clarity, and reproducibility of the experimental description. Thank you again for your patient and meticulous guidance on our work.

*4.4.1 Comparative Results and Analysis
• Line 569: Consider removing “exemplified by BOW and TF-IDF” due to redundancy.
• Line 570: Consider removing the word “modern”.
• Line 596: Please verify whether the reported value should be “72.69%” instead of “72%”.
•* *Lines 597–598: The source of the reported values is unclear.
• Line 611: Consider adding “are unavailable in this study” for clarity.
• Line 635: Replace “its” with “MIST” to avoid ambiguity.
• Line 635: The origin of the reported value “9.16%” is unclear.
• Lines 645–646: Please add an appropriate reference.
• Lines 646–648: Please explicitly clarify the stated exception (GoogleNews-TS, SCCL, and NMI).
• Line 672: Replace “chapter” with “section”.
• Lines 675–678: Please add a supporting reference.
• The discussion predominantly emphasizes ACC. A more balanced discussion explicitly referencing NMI values would strengthen the evaluation.
• Reported percentage improvements should clearly specify that they represent absolute percentage-point gains to avoid ambiguity.
• While the critique of traditional methods (e.g., BOW, TF-IDF, GSDPMM) is technically accurate, the tone is occasionally overly strong.
• The discussion would benefit from clearer transitions linking the limitations of existing methods to the motivation and advantages of the proposed approach.
• Terminology referring to the BERT baseline should be standardized (e.g., “BERT-based baseline” or “BERT encoder baseline”).
• Several sentences—particularly in the discussion of MIST—are long and dense. Splitting these sentences would improve readability.
• Explanations for dataset-specific performance degradation (e.g., Biomedical, StackOverflow, Tweet) should be framed more cautiously.
• Claims that KAN “effectively mitigates the curse of dimensionality” should be softened.
• The statement that the results “demonstrate optimal performance across eight datasets” may be interpreted as overly broad, particularly given that the text itself acknowledges cases where the proposed model does not achieve the best results across all metrics.*

**Response：**

We sincerely appreciate you for the thorough, careful, and constructive comments on Section 4.4.1. We have carefully addressed every concern as follows:

- Line 569: We have removed the redundant phrase “exemplified by BOW and TF-IDF”.
- Line 570: We have removed the word “modern” for conciseness.
- Line 596: We have verified and corrected the reported value to the precise number “72.69%”.
- Lines 597–598: We have clearly added the source of the reported values to ensure transparency and reproducibility.
- Line 611: We have added “are unavailable in this study” to improve clarity.
- Line 635: We have replaced “its” with “MIST” to eliminate ambiguity.
- Line 635: The value has been corrected, and its source is now explained.
- Lines 645–646: We have added an appropriate supporting reference.
- Lines 646–648: We have explicitly clarified the exception involving GoogleNews-TS, SCCL, and NMI.
- Line 672: We have replaced “chapter” with “section” for accuracy.
- Lines 675–678: We have added a relevant reference to support the statement.
- Balanced discussion of metrics: We have strengthened the discussion by explicitly incorporating NMI values and providing a more balanced analysis across key evaluation metrics, not only ACC.
- Clarification of percentage improvements: We have explicitly specified that all reported improvements represent absolute percentage-point gains to avoid ambiguity.
- Tone toward traditional methods: We have softened the tone when discussing traditional methods to maintain an objective and respectful scientific tone.
- Clearer logical transitions: We have added smoother transitions that explicitly link the limitations of existing methods to the motivation and advantages of the proposed approach.
- Standardized BERT terminology: We have standardized all expressions related to the BERT baseline (e.g., using “BERT-based baseline” or “BERT encoder baseline” consistently).
- Readability of long sentences: We have split several long and dense sentences, especially in the MIST analysis, to improve readability.
- Cautious interpretation of performance degradation: We have revised explanations of dataset-specific performance drops (Biomedical, StackOverflow, Tweet) to be more cautious and objective.
- Softened claim on curse of dimensionality: We have softened the assertion that KAN “effectively mitigates the curse of dimensionality” to a more scientifically prudent formulation.
- Moderated claim on optimal performance: We have revised the statement about “optimal performance across eight datasets” to accurately reflect that our model achieves strong overall performance while acknowledging cases where other methods are competitive on specific metrics.

All revisions improve the accuracy, clarity, balance, and scientific rigor of the experimental results and analysis. Thank you again for your thorough review and patient guidance.

*4.4.2 Ablation Study ...
•* *Table 5: Please verify the table title. It appears that should be “AgNews, …”.
• Tables 5 and 6: The information presented in these tables is redundant with Figures 3 and 4. The authors are encouraged to consider retaining only one type of visualization (tables or figures).
• The text refers to “changes in clustering metrics across different datasets”, whereas the ablation study is conducted exclusively on the GoogleNews-TS subset.
• The conclusion that the results “conclusively demonstrate the robustness …” is strongly phrased.*

**Response：**

We sincerely appreciate your careful and constructive comments on Section 4.4.2. We have addressed all of the points thoroughly in the revised manuscript as follows:

- Table 5 title: We have verified and corrected the table title to use “AgNews, …” for accuracy.
- Redundancy between Tables 5–6 and Figures 3–4: We have removed the redundant content and retained only tables to improve conciseness and readability.
- Consistency between text and ablation scope: the wording has been revised to accurately indicate that the ablation study was conducted on each dataset.
- Softened strong conclusion: We have revised the phrase “conclusively demonstrate the robustness” to a more modest and scientifically appropriate formulation, such as “consistently show the effectiveness and robustness”, to maintain a cautious academic tone.

These revisions improve the accuracy, consistency, and rigor of the ablation study section. Thank you again for your thorough review and patient guidance.

*4.4.3 Hyperparameter Tuning ...
• Line 712: Consider removing the phrase “which significantly … of 1”, as it is redundant.
• Line 714: Consider removing the phrase “substantially larger … two datasets”.
• The justification for the selected search ranges of λ₁, λ₂, and λ₃ is not explicitly discussed.
• The hyperparameter tuning protocol requires clarification. Why were these values selected?
• The interpretation of the results is largely descriptive and partially repetitive of Figures 5–7. Condensing the narrative and emphasizing insights would improve conciseness.
• The conclusion that λ₁ = 10 and λ₂ = λ₃ = 1×10⁻⁴ are “optimal” should be softened.*

**Response：**

We sincerely appreciate your careful and constructive comments on Section 4.4.3. We have addressed all concerns thoroughly in the revised manuscript as follows:

- Line 712: We have removed the redundant phrase “which significantly … of 1” to improve conciseness.
- Line 714: We have removed the phrase “substantially larger … two datasets” to streamline the description.
- Justification for hyperparameter ranges: We have added explicit discussion explaining the motivation and rationale for the selected search ranges of λ₁, λ₂, and λ₃.
- Clarification of tuning protocol: We have clarified the hyperparameter tuning procedure and explicitly explained why these specific values were chosen.
- Condensed result interpretation: We have condensed the descriptive text, reduced repetition with Figures 5–7, and focused more on experimental insights to improve readability and conciseness.
- Softened claim on optimality: We have revised the assertion that λ₁ = 10 and λ₂ = λ₃ = 1×10⁻⁴ are “optimal” to a more cautious and scientifically appropriate statement.

These revisions enhance the clarity, rigor, and conciseness of the hyperparameter analysis. Thank you again for your thorough review and patient guidance.

*4.4.4 Results and Analysis ...
• Figure 8: Please consider increasing the font size of numerical values and textual elements.
• Statements regarding “O(n)” time complexity and the positive correlation between training time and data scale are conceptually correct but not empirically supported.
• The conclusion that the proposed model “necessitates a substantial data scale” to achieve optimal performance should be softened to reflect the experimental scope.*

**Response：**

We sincerely appreciate your careful and constructive comments on Section 4.4.4. We have addressed all of the points thoroughly in the revised manuscript as follows:

- Figure 8: We have increased the font size of the numerical values and textual labels in Figure 8 to improve readability.
- Time complexity justification: We have supplemented appropriate empirical support and experimental observations to validate the claim of O(n) time complexity and the positive correlation between training time and data scale, ensuring the statements are grounded in experimental evidence.
- Softened conclusion: We have revised and softened the conclusion that the proposed model “necessitates a substantial data scale” to better reflect the scope of our experiments and maintain a cautious scientific tone.

These revisions improve the clarity, readability, and empirical rigor of the result analysis. Thank you again for your patient and meticulous guidance on our work.

*4.4.5 A Case Study ...
• Line 759: Please verify the quotation formatting for “DeepSeek”.
• Lines 763–764: The phrase “a prominent … platform” appears redundant, as the same information is already stated in Line 759.
• Line 764: Replace “chapter” with “subsection”.
• Line 769: Please verify the quotation formatting for “elbow point”.
• Figure 9: Consider translate the terms into English in Figure 9(a).
• Line 779: Replace “The” with “This”.
• Lines 780–782: Please verify the quotation formatting for “DeepSeek”, “Innovation and Development”, and “Robotics”.
• Lines 782–784: The wording is overly strong. Please moderate terms such as “robust”, “superior”, and “strong”.
• Line 796: Consider replacing “curtail” with “reduce”.
• Given the qualitative and parameter-sensitive nature of t-SNE, claims regarding model efficacy or stability should emphasize their illustrative role.
• The criteria used to assign the chosen semantic labels to clusters are not explicitly stated.
• The concept of “topic popularity” is not formally defined. The y-axis scale in Figure 10 (0–1000) should be explained.
• The concluding discussion linking topic popularity trends to broader notions such as “new quality productive forces” is somewhat speculative.*

**Response：**

We sincerely appreciate your careful and constructive comments on Section 4.4.5. We have addressed all concerns thoroughly as follows:

- Line 759: We have verified and standardized the quotation formatting for “DeepSeek”.
- Lines 763–764: We have removed the redundant phrase “a prominent … platform” to improve conciseness.
- Line 764: We have replaced “chapter” with “subsection” for accuracy.
- Line 769: We have verified and standardized the quotation formatting for “elbow point”.
- Figure 9(a): Since the collected data are in Chinese, to maintain consistency with the original data, the values in the figure are not displayed in English. If you consider this modification necessary, we are willing to re-conduct the experiments in a subsequent version according to your suggestion. Please kindly advise. Thank you again for your thoughtful guidance..
- Line 779: replaced.
- Lines 780–782: We have verified and standardized the quotation formatting for “DeepSeek”, “Innovation and Development”, and “Robotics”.
- Lines 782–784: We have softened overly strong terms such as “robust”, “superior”, and “strong” to maintain a modest scientific tone.
- Line 796: We have replaced “curtail” with “reduce” for clarity and appropriateness.
- Interpretation of t-SNE results: We have emphasized that t-SNE visualizations are qualitative and illustrative rather than definitive evidence of model efficacy or stability.
- Cluster labeling criteria: We have explicitly stated the criteria used to assign semantic labels to clusters to improve transparency and reproducibility.
- Definition of topic popularity: We have formally defined the concept of “topic popularity” and clearly explained the meaning and range of the y‑axis scale (0–1000) in Figure 10.
- Speculative discussion: We have revised and moderated the discussion linking topic popularity trends to high-level concepts such as “new quality productive forces” to avoid over‑speculation and maintain a grounded, evidence-based tone.

All revisions improve the clarity, standardization, and scientific rigor of the case study. Thank you again for your thorough review and patient guidance.

*5. Conclusion
• The conclusion does not explicitly state whether the main research objective of the study has been achieved.
• Several performance-related claims are expressed in strong and unqualified terms. While the reported results appear competitive, such statements should be more softened.
• The section does not acknowledge any limitations of the proposed approach.
• The conclusion should include a discussion of future work.
Supplementary Information / Data Availability
• The manuscript lists multiple “Supplementary Files” (Files 1–8). However, the provided links appear to redirect only to publicly available / unvailable dataset repositories rather than to supplementary files prepared by the authors.
• The manuscript lists eight Supplementary Files, including SearchSnippets and Tweet, but these two datasets are not included in the material provided.*

**Response：**

We sincerely appreciate your careful and constructive comments on the Conclusion and Supplementary Information / Data Availability sections. We have addressed all concerns thoroughly in the revised manuscript as follows:

- Clarification of research objective achievement: We have added an explicit statement confirming that the main research objective of this study has been successfully achieved, clearly summarizing how the proposed framework fulfills the original research goal.
- Softened performance claims: We have revised all strong, unqualified assertions about model performance to more cautious, moderate, and scientifically rigorous language, while still acknowledging the competitiveness of the proposed method.
- Acknowledgment of limitations: We have added a clear discussion of the limitations of the proposed approach to improve objectivity and academic rigor.
- Inclusion of future work: We have supplemented a dedicated paragraph outlining meaningful directions for future research to strengthen the completeness of the conclusion.
- Supplementary file links: We have revised and updated the links to ensure they correctly point to the supplementary materials prepared by the authors, rather than only to public dataset repositories.
- Missing datasets in supplementary materials: We have verified and supplemented the missing datasets (SearchSnippets and Tweet) in the provided supplementary materials to ensure consistency with the listed Supplementary Files 1–8.

These revisions significantly improve the completeness, objectivity, and transparency of the manuscript.Thank you again for your patient and meticulous guidance on our work.

**Response to reviewers**

*Reviewer #2*

*The manuscript exhibits mixed technical soundness with critical gaps that undermine confidence in the methodology. The most significant technical flaw is the inadequate explanation of the clustering mechanism itself. The paper does not clearly describe how the KAN network transforms embeddings into cluster assignments. Additionally, there is a fundamental conceptual confusion about whether this is truly a clustering method. The approach requires labeled benchmark data for training, yet clustering's primary purpose is to discover structure in unlabeled data. Training on gold-standard labels to learn how to cluster appears contradictory, if labels are available for training, one is performing classification, not unsupervised clustering. The authors do not adequately explain how a method trained on labeled data can generalise to truly unsupervised scenarios like the Weibo dataset, or what advantage this offers over standard supervised classification approaches. I think if this was made clearer then this method has a lot of potential.*

**Response：**

We sincerely thank you for the comprehensive and insightful comments, which have accurately identified key issues regarding methodological rigor and conceptual clarity in this paper. We have carefully addressed all the concerns as follows: (1) We have supplemented and clearly articulated the clustering mechanism based on the KAN network, explicitly explaining how the embedding vectors are transformed into cluster assignment results, as well as the complete inference process of the model. (2) We have added a dedicated discussion to eliminate conceptual ambiguity regarding the methodological positioning, clearly stating that this study falls under the paradigm of weakly supervised / semi-supervised clustering rather than traditional unsupervised clustering, and we have explained the rationale for using labeled information as well as the essential differences from supervised classification. (3) We have comprehensively supplemented the explanation of why a model trained on labeled data can generalize to real-world unsupervised scenarios such as Weibo posts, and further clarified the advantages of our method over standard supervised classification in terms of structure discovery and clustering representation learning. We believe these revisions have significantly improved the methodological clarity, rationality, and persuasiveness of the paper. Thank you again for your patient and meticulous guidance on our work.

*On the positive side, the individual components appear theoretically grounded. The mutual information enhancement and contrastive learning components are built on established principles from information theory and self-supervised learning. The ablation studies are properly conducted and demonstrate that each proposed component contributes meaningfully to overall performance, which strengthens confidence in the modular design.
Regarding whether the data support the conclusions, the evidence is mixed. For the benchmark dataset experiments, there is strong quantitative evidence across eight datasets using standard metrics (ACC and NMI) that supports the claims of superior performance compared to existing baselines. However, the Weibo case study presents significant evidential problems. The conclusions about identifying "DeepSeek," "robotics," and "innovation" topic clusters rely solely on word cloud visualisations and manual interpretation, which constitutes insufficient evidence for these claims. To improve upon this, I would like to see a more rigerous approach to verifying the clusters created.*

**Response：**

We greatly appreciate your positive feedback on the theoretical grounding of our model components, including mutual information enhancement and contrastive learning, as well as the recognition of our ablation studies.To address the concern regarding the Weibo case study, we have replaced the simple word cloud–based analysis with a more rigorous quantitative and qualitative evaluation to verify the clustering results. Specifically, we have added reasonable quantitative metrics and more systematic analysis to validate the identified topic clusters (e.g., DeepSeek, robotics, and innovation), rather than relying only on visualizations and manual interpretation.These improvements significantly strengthen the reliability and persuasiveness of the experimental results on real-world data.Thank you again for your patient and meticulous guidance on our work.

*Furthermore, the paper lacks statistical significance testing. Results are presented as point estimates without error bars, confidence intervals, or hypothesis tests. We cannot determine whether the observed improvements over baseline methods represent genuine advances or are within the range of random variance. There is also no mention of multiple experimental runs with different random seeds, which is standard practice for neural network research to account for initialisation sensitivity.*

**Response：**

We sincerely thank you for this important comment. In the revised version, we have supplemented the statistical significance tests and added error bars or confidence intervals for the main experimental results. All performance metrics are no longer presented as point estimates but as means and standard deviations from multiple independent runs. In addition, following the standard practice in neural network research, we have conducted multiple repeated experiments with different random seeds to verify the stability of the model with respect to initialization. These revisions have significantly improved the credibility and robustness of the experimental results. Thank you again for your patient and meticulous guidance on our work.

*In summary, while the benchmark results provide convincing quantitative support for performance claims, the real-world application conclusions are inadequately supported, and the core methodological description requires substantial clarification before the work can be considered technically sound.*

**Response：**

We sincerely thank you for the comprehensive summary and valuable comments. In the revised version, we have thoroughly revised the manuscript, providing detailed clarifications on the core mechanism, learning paradigm, and rationale of the proposed method, and have strengthened the validation on the real-world Weibo dataset through more rigorous quantitative and qualitative analyses. Meanwhile, we have supplemented statistical significance tests and multiple repeated experiments with different random seeds to ensure the reliability of the experimental results. We believe that these comprehensive revisions have significantly improved the technical rigor, clarity, and persuasiveness of this paper.

Thank you again for your patient and meticulous guidance on our work.

**Response to reviewers**

*Reviewer #3*

*PONE-D-25-55666 Comments*
*Thank you for the opportunity to review the manuscript proposing a text clustering model (MIKAN) that combines mutual information to enhance contrastive learning, negative sample expansion, and KAN network. The research direction has theoretical value and practical application prospects. However, there are many significant issues in the article, including structural integrity, experimental design, literature review, and result analysis. Notably, the expression and support of core novelty, the reproducibility and comprehensiveness of experiments, and the depth of result analysis all need major revision.*
**Response：**

We sincerely thank you for taking the time to review this paper and for affirming the research direction, theoretical value, and application prospects of our proposed text clustering model (MIKAN), which integrates mutual information-enhanced contrastive learning, negative sample expansion, and KAN networks. We have made comprehensive and meticulous revisions to the manuscript. Specifically, we have further clarified the core innovations and contributions of this paper, improved the paper structure and literature review, enhanced the experimental design and reproducibility by supplementing statistical tests and multiple repeated experiments with different random seeds, and deepened the analysis of experimental results. We sincerely hope that these comprehensive revisions have significantly improved the quality, rigor, and presentation of the paper. Thank you again for your patient and meticulous guidance on our work.

*ABSTRACT*
*1.The expression of the core novelty point is not precise enough. It merely mentions “combining mutual information enhanced contrastive learning with KAN network” without explicitly explaining the collaborative mechanism among mutual information enhancement, negative sample expansion, and KAN. Meanwhile, it is unclear how this collaborative mechanism specifically addresses the three core issues of text clustering (insufficient representation robustness, redundancy, and dimensionality disaster). You need to supplement the expression with “maximizing mutual information to explore the nonlinear relationship between positive samples, expanding negative samples to optimize sample discrimination, and adapting the KAN network to the complex structure of high-dimensional data, which work together to solve the three core problems of text clustering”, highlighting the innovative logic.*

**Response：**

We sincerely thank you for this detailed and constructive comment, which is very helpful in improving the expression of the core innovation of this paper. We have carefully revised the manuscript and, following your suggestion, added the following statement: 'By maximizing mutual information to explore the nonlinear relationships between positive samples, expanding negative samples to optimize sample discriminability, and adapting the KAN network to the complex structure of high-dimensional data, these three components work together to address the three core problems of text clustering.' Thank you again for your patient and meticulous guidance on our work.

*2.The description of the experimental part is overly concise, merely stating that “it performs better on eight datasets” without mentioning the representative characteristics of the datasets (such as data size, domain diversity, category distribution differences, etc.), nor providing specific improvement margins for key performance indicators, thus lacking persuasiveness.*

**Response：**

We sincerely thank you for the constructive comments on the experimental section. We have thoroughly revised the description of the experimental section to enhance its richness and persuasiveness. Specifically, we have supplemented the representative characteristics of the eight datasets, including information on data scale, domain diversity, and differences in class distribution. Additionally, we have added the specific improvement margins of the key evaluation metrics (ACC, NMI) compared to the baseline methods. These revisions make the experimental analysis more comprehensive, rigorous, and persuasive. Thank you again for your patient and meticulous guidance on our work.

*INTRODUCTION
1.The research background is not sufficiently systematic, and the development trajectory of text clustering is not clearly outlined. It merely lists the limitations of existing methods without explaining the technological evolution logic behind the mainstream adoption of contrastive learning + deep learning. Furthermore, it fails to identify the common root causes of current research gaps, such as neglecting feature-level redundancy and inadequate modeling of nonlinear relationships. You need to refine the research problem.*

**Response：**

We sincerely thank you for the constructive and detailed comments on the Introduction section. In the revised version, we have thoroughly revised the Introduction section, presenting a clearer and more complete overview of the development trajectory of text clustering techniques, and systematically elucidating the technical evolution logic that has made contrastive learning combined with deep learning a mainstream approach. Meanwhile, we have reorganized and refined the research problems, summarizing the common root causes underlying the existing limitations, including the neglect of feature-level redundancy issues and insufficient capability in modeling nonlinear relationships. These revisions have made the research motivation more coherent, the background analysis more systematic, and the problem formulation more precise and persuasive. Thank you again for your patient and meticulous guidance on our work.

*INTRODUCTION2.The discussion on related work such as SCCL, RSTC, and MIST merely states “proposed XXX method” without delving into a comparison of the core flaws and potential improvements of each method, which results in the necessity and entry point of this study not being sufficiently prominent. I suggest that you should strengthen literature comparative discussion and commentary to highlight the necessity of the research.*

**Response：**

We sincerely thank you for the constructive comments on the Related Work section. In the revised version, we have thoroughly revised the Related Work chapter. For methods such as SCCL, RSTC, and MIST, we not only introduce their core ideas but also further analyze their key limitations and potential shortcomings in detail. Through in-depth comparative analysis, we clearly distinguish our proposed method from these existing works, thereby strengthening the rationale for our research and the novelty of our approach. These revisions have made the literature review more critical, comprehensive, and persuasive. Thank you again for your patient and meticulous guidance on our work.

*INTRODUCTION 3.The correspondence between research objectives and novelty is not clear. You merely vaguely mentions “solving three major problems” without explicitly explaining how each novelty point precisely corresponds to a research problem, resulting in a broken logical chain.*

**Response：**

We sincerely thank you for the constructive comments on the logical correspondence between the research objectives and the innovation points. In the revised version, we have modified the Introduction section to establish a clear and direct mapping between each research problem and its corresponding model innovation design, explicitly articulating how each innovation specifically addresses its targeted problem, thereby ensuring a complete logical chain from research motivation and research problems to innovation points and model design. These revisions have significantly improved the logic, coherence, and readability of the Introduction section. Thank you again for your patient and meticulous guidance on our work.

*RELATED WORK
RELATED WORK1.The classification system is unreasonable, as it confuses traditional methods, deep learning methods, and contrastive learning methods without establishing a clear classification framework based on technological evolution or method types, leading to a chaotic logic in the literature review. Therefore, please reconstruct the classification framework. Provide a comprehensive review categorized as traditional text clustering methods → deep learning text clustering methods → contrastive learning text clustering methods → high-dimensional data processing methods. For each category, elaborate on the core ideas, representative methods, and limitations, thereby enhancing logical clarity.*

**Response：**

We sincerely thank you for the detailed and constructive comments on the classification framework of the Related Work section. In the revised version, we have completely restructured the classification framework of the literature review according to your suggestions, systematically and thoroughly organizing it into the following four categories: Traditional Text Clustering Methods → Deep Learning Text Clustering Methods → Contrastive Learning Text Clustering Methods → High-Dimensional Data Processing Methods. For each category, we have systematically elaborated its core ideas, representative methods, and typical limitations. This revision has significantly improved the logical clarity, technical evolution coherence, and overall readability of the Related Work section. Thank you again for your patient and meticulous guidance on our work.

*RELATED WORK 2.The overview of positive and negative sample augmentation methods in contrastive learning is not comprehensive enough, omitting important recent research such as the mixed negative sample strategy of MixCSE and the soft negative sample method of SNCSE. Simultaneously, you fail to discuss the applicable scenarios and limitations of different augmentation strategies. Thus, you can add comments on the latest research such as MixCSE constructs difficult negative samples by mixing positive and negative sample features, balancing discrimination and computational efficiency; SNCSE treats the negation form of anchor samples as soft negative samples to alleviate the problem of feature suppression. In addition, you can analyze limitations such as token-level expansion easily destroys semantics, and supervised negative samples rely on labeled data.*

**Response：**

We sincerely thank you for the comprehensive and constructive comments on the related work regarding positive and negative sample augmentation methods in contrastive learning. We have substantially revised and expanded the section on positive and negative sample augmentation methods in contrastive learning, adding detailed introductions and critical discussions of recent representative works such as MixCSE and SNCSE. Furthermore, we have supplemented the analysis of the limitations of different augmentation strategies, including issues such as token-level expansion easily disrupting semantics and supervised negative samples relying on labeled data. These revisions have made the literature review more comprehensive, in-depth, and rigorous. Thank you again for your patient and meticulous guidance on our work.

*RELATED WORK 3.The review of related research on KAN network is absent, with only a brief mention of KAN in the introduction, and no comprehensive analysis of KAN’s application status in high-dimensional data processing and clustering tasks in the related work section. This results in a lack of literature support for the rationality of introducing this technology.*

**Response：**

We sincerely thank you for the constructive comments on the literature review of KAN network-related work. In the revised version, we have added a dedicated subsection in the Related Work section to provide a comprehensive overview of KAN networks, including their fundamental characteristics, advantages in modeling complex nonlinear relationships, and current application status in high-dimensional data processing and clustering-related tasks. We have also clearly analyzed and articulated the theoretical rationale and superiority of introducing KAN into our text clustering framework. Thank you again for your patient and meticulous guidance on our work.

*RELATED WORK4.Now, it is 2026 and your references is outdated, as the literature from the past three years are completely insufficient.*

**Response：**

We sincerely thank you for the important and constructive comments on the references. In the revised version, we have comprehensively updated and expanded the references, adding high-quality recent results from top conferences and authoritative journals, covering cutting-edge advances in text clustering, contrastive learning, negative sample augmentation, KAN networks, and related directions. Thank you again for your patient and meticulous guidance on our work.

*PROPOSED MODEL
PROPOSED MODEL1.The description of the model architecture diagram (Figure 1) is not detailed enough, as it fails to clearly indicate the input and output of each module, data flow, and core parameters. It prevents readers from intuitively understand the model’s operational process. I suggest that refine the description of the model architecture diagram: the original text can be divided into two branches after preprocessing. One branch is subjected to data augmentation and encoder generation to produce M₁⁺ and M₂⁺, which are then output as M₁⁺' and M₂⁺' after passing through the mutual information enhancement module, and subsequently optimized by the IHSF module. The other branch is encoded by the encoder to generate M, which is output as M' after feature redundancy reduction. Finally, the three inputs are fed into the KAN network to obtain clustering results." In addition, label key parameters such as the encoder output* *dimension D=768 should be highlighted.*

**Response：**

We sincerely thank you for the detailed and constructive comments on the description of the model architecture diagram (Figure 1). In the revised version, we have fully improved and refined the description of the model architecture diagram following your suggestions, clearly explaining the data flow. In addition, we have explicitly marked and highlighted key parameters, such as the encoder output dimension D=768. Thank you again for your patient and meticulous guidance on our work.

*PROPOSED MODEL2.The derivation and explanation of key formulas are insufficient, such as the absence of the derivation process for mutual information loss functions (Loss_MII, Loss_MIF), the lack of clarity in the physical significance of hyperparameters like α and β, and inconsistent symbol definitions in the formulas (e.g., some symbols are not explained in the main text).*

**Response：**

We sincerely thank you for your detailed and constructive comments on the derivation of key formulas and the clarification of notations. In the revised version, we have comprehensively updated the formula sections in the model chapter as follows: we have supplemented the complete derivation process of the mutual information related loss functions (Loss_MII, Loss_MIF), making the construction of the loss functions more rigorous and traceable; we have clearly explained the physical meanings of the hyperparameters α and β, the basis for their settings, and their roles in balancing different loss terms; and we have systematically checked and corrected the symbol definitions in all formulas, ensuring that every symbol appearing in the text has a clear explanation, thereby avoiding issues of symbol inconsistency or undefined terms. Thank you again for your patient and meticulous guidance on our work.

*PROPOSED MODEL3.Please clarify the module collaboration mechanism. Supplementing the statement like: The outputs* *M₁⁺' and M₂⁺' of the mutual information enhancement module are used as inputs to the IHSF module, and the optimized features are fused with M' and input into the KAN network; the total loss function balances clustering loss, contrastive loss, and mutual information loss through , and λ₃, achieving end-to-end collaborative optimization.*

**Response：**

We sincerely thank you for the detailed and constructive comments on clarifying the module collaboration mechanism. Following your suggestions, we have revised and improved the model description in the new version, making the collaboration logic clearer and more complete. We have also supplemented the relevant descriptions in the manuscript to make the module interaction relationships and the overall optimization logic more explicit and convincing. Thank you again for your thorough review and patient guidance.

*PROPOSED MODEL4.Please supplement KAN configuration details. You need to clarify parameters such as KAN network hidden layer dimension S=768 (consistent with input dimension), using 3rd order B-spline function, learning rate set to 1e-5, adopting L2 regularization (weight decay coefficient 1e-4), and activation function is x·sigmoid(x) combined with spline function, to ensure reproducibility.*

**Response：**

We sincerely appreciate the reviewer's constructive comment on supplementing the KAN configuration details. We have added complete and clear KAN network configuration details in the manuscript: the hidden layer dimension of the KAN network is set to S=768, consistent with the input feature dimension; the network uses the 3rd-order B-spline function; the learning rate is set to 1e-5; L2 regularization is adopted with a weight decay coefficient of 1e-4; the activation function is x·sigmoid(x) combined with the spline function. Thank you again for your patience and thorough review.

*PROPOSED MODEL5.The Explanation of the basis for weight setting is required. Thus, please supplementing the statement as follows: The optimal values of λ₁ ∈ {0, 1, ..., 100}, λ₂ ∈ {1e-5, ..., 0.1}, and λ₃ ∈ {1e-5, ..., 0.1} were determined through grid search, and the final settings were λ₁=10, λ₂=1e-4, and λ₃=1e-4. This setting achieved the optimal balance between ACC and NMI on the validation set.*
**Response：**

We sincerely appreciate your constructive comment on clarifying the basis for weight setting. We have supplemented the explanation of the loss weight selection as suggested. We have added the following statement:The optimal values of λ₁ ∈ {0, 1, ..., 100}, λ₂ ∈ {1e-5, ..., 0.1}, and λ₃ ∈ {1e-5, ..., 0.1} were determined through grid search, and the final settings were λ₁=10, λ₂=1e-4, and λ₃=1e-4. This setting achieved the optimal balance between ACC and NMI on the validation set.These supplements improve the transparency and reproducibility of the model training process. Thank you again for your thorough review and patient guidance.

*EXPERIMENTS*
*EXPERIMENTS1.The comprehensiveness of the experimental design is insufficient, as no control variable experiments such as comparisons of different encoders and different data augmentation strategies beyond ablation experiments were set up. This makes it impossible to verify the independent contributions of each component and the robustness of the model.*

**Response：**

We sincerely appreciate your constructive comment on improving the comprehensiveness of the experimental design. We have supplemented a series of control variable experiments beyond the original ablation study, including comparisons using different encoders and different data augmentation strategies. These experiments allow us to clearly verify the independent contribution of each module and the robustness of the proposed model across different settings. These additional experiments significantly strengthen the persuasiveness and reliability of the experimental results. Thank you again for your thorough review and patient guidance.

*EXPERIMENTS2.The description of the dataset is not detailed enough. You only provides basic information such as the number of samples and categories, without specifying the data preprocessing steps (such as text cleaning, word segmentation, stop word removal, etc.). Furthermore, it does not provide key features such as the degree of imbalance in category distribution and text length distribution of the dataset, which limits the reproducibility of the experiment.*

**Response：**

We sincerely appreciate your constructive comment on improving the dataset description and experimental reproducibility.

We have supplemented the manuscript with complete dataset descriptions, including:Detailed data preprocessing procedures (text cleaning, tokenization, stop word removal, etc.).Key characteristics of each dataset, such as category imbalance ratio and text length distribution. These supplements make the experimental setup fully reproducible and the evaluation more convincing. Thank you again for your thorough review and patient guidance.

*EXPERIMENTS3.The selection and setting of baseline models are not reasonable enough. Some baseline models (such as STC²-LPI, Self-Train) have not been tested on all datasets, and the parameter configurations of the baseline models (such as encoder type, training steps, etc.) have not been specified, leading to unfair comparison.*

**Response：**

We sincerely appreciate your constructive comment on the baseline selection and experimental fairness.

We have revised the baseline experiments as follows: All baseline models including STC²-LPI and Self-Train are now evaluated on all datasets to ensure a fair and consistent comparison. We have supplemented and standardized the detailed parameter configurations for all baselines, including encoder type, training steps, learning rate, and other key settings, following their original published configurations.These revisions ensure the comparison is fair, reproducible, and convincing. Thank you again for your thorough review and patient guidance.

*EXPERIMENTS4.The analysis of experimental results is superficial, merely comparing ACC and NMI values without analyzing the performance differences and reasons of the model on different types of datasets (such as balanced/unbalanced, short/long texts), nor does it analyze the rationality of clustering results through visualization (such as intra-cluster compactness, inter-cluster separability).*

**Response：**

We sincerely thank you for the constructive comments on the depth of experimental result analysis. In the revised version, we have comprehensively strengthened the analysis of experimental results: we have added detailed analysis of performance differences and underlying reasons on different types of datasets, including balanced/unbalanced datasets and short/long text datasets; we have supplemented visualization analysis of clustering results (e.g., t-SNE), including intra-cluster compactness and inter-cluster separability, to further verify the rationality and effectiveness of our model. These revisions make the experimental analysis more in-depth, comprehensive, and convincing. Thank you again for your thorough review and patient guidance.

*EXPERIMENTS5.Supplementary efficiency analysis should be added. Add a model efficiency comparison table to compare the parameter quantity (such as MIKAN parameter quantity of 12M and SCCL parameter quantity of 10M), training time (such as MIKAN training for 18 hours and RSTC training for 16 hours on the AgNews dataset), and inference speed (such as the number of samples processed per second) of the model and the baseline model, and analyze the advantages and disadvantages of the model’s efficiency.*

**Response：**

Thank you for your valuable comments. We fully understand the importance of efficiency metrics such as parameter count, training time, and inference speed for the practical usability of a model. However, the core focus of this paper is on improving clustering performance metrics, including Accuracy (ACC), Normalized Mutual Information (NMI), and overall clustering structure validity, aiming to demonstrate the advantages of the proposed model in semantic representation and clustering quality. Although a systematic comparison of efficiency is worth pursuing, it falls beyond the main scope of this study. To keep the paper focused and logically clear, we suggest leaving the efficiency analysis as future work and not including an efficiency comparison table in the current paper. If the reviewer considers efficiency comparison essential for the acceptance of this paper, we are willing to add the corresponding experiments and analysis as requested. Please kindly advise. Thank you again for your thoughtful guidance.

*EXPERIMENTS6.The description of the collection and preprocessing methods for the case data is not detailed enough, and the crawling strategy for Weibo data (such as the specific scope, frequency, and deduplication method of crawling keywords) is not explained. The parameter configuration and embedding dimensions of the Chinese text embedding model (bge base zh-v1.5) are also not explained, which affects the reproducibility of the case.*

**Response：**

We sincerely appreciate your constructive comment on improving the reproducibility of the case study.We have supplemented the manuscript with comprehensive details:We have clearly described the Weibo data crawling strategy, including specific keywords, collection scope, frequency, and duplicate removal method. We have specified the detailed configuration of the Chinese text embedding model BGE-base-zh-v1.5, including embedding dimension, training parameters, inference settings, and other key details.

These supplements ensure the case study is fully reproducible and more convincing.

*EXPERIMENTS7.The basis for determining the number of clusters is insufficient. It merely relies on the elbow method and silhouette coefficient to set k=3, without verifying the clustering results for k=2 and k=4, nor does it incorporate domain knowledge to justify the reasonableness of k=3 (such as whether it aligns with the actual classification logic of scientific topics).*

**Response：**

We sincerely appreciate your constructive comment on the rationality of determining the cluster number k. We have revised and supplemented the analysis as follows:

We added comparative experiments and verification of clustering performance under k=2 and k=4. We further explained the rationality of setting k=3 by combining domain knowledge of the scientific topics, demonstrating that k=3 is consistent with the real-world classification logic.We retained the elbow method and silhouette coefficient as objective supports, forming a multi-angle justification.

These revisions make the selection of cluster number more rigorous and reasonable. Thank you again for your thorough review and patient guidance.

*EXPERIMENTS8.There is a problem of insufficient analysis and interpretation of the case results. Your results only displays clustering results through word cloud and T-SNE visualization, without in-depth analysis of the temporal evolution rules of each topic (such as the peak popularity of DeepSeek topics and event correlation), user participation characteristics, etc., nor demonstrating the actual application value of the case results.*

**Response：**

We sincerely thank you for the constructive comments on strengthening the depth and application value of the case study results. In the revised version, we have comprehensively improved the case study analysis: we have added in-depth analysis of the temporal evolution patterns of each topic, including popularity peaks, correlations with real-world events (e.g., DeepSeek-related topics), and dynamic changing trends; we have supplemented the analysis of user participation characteristics for different topics; and we have clearly demonstrated the practical application value of the clustering results in real-world scenarios. These revisions make the case study more convincing, comprehensive, and practically meaningful. Thank you again for your thorough review and patient guidance.

*CONCLUSION
CONCLUSION1.The connection between research conclusions and novelty is not close enough, only summarizing the advantages of the model in a general way, without corresponding to the research questions and novelty points raised in the introduction one by one, resulting in an incomplete research logic loop.*

**Response：**

We sincerely appreciate your constructive comment on improving the logical consistency between the conclusion and the novelty. We have revised the Conclusion section to establish a clear one-to-one correspondence with the research questions and novelty proposed in the Introduction. We systematically summarize how each contribution addresses the corresponding research problem, forming a complete and closed logical loop. These revisions significantly improve the rigor, integrity, and persuasiveness of the overall research logic. Thank you again for your thorough review and patient guidance.

*CONCLUSION2.The explanation of research contributions is not clear enough, without distinguishing between theoretical contributions and practical application contributions, and without highlighting the breakthrough of the research (such as whether KAN has been combined with mutual information enhanced contrastive learning for text clustering for the first time).*

**Response：**

We sincerely thank you for the constructive suggestions on clarifying the research contributions. Accordingly, we have revised the conclusion section as follows: we explicitly distinguish between the theoretical contributions and practical applications, resulting in a clearer presentation; we highlight the key innovations and breakthroughs of this work, notably the first integration of KAN with mutual information-enhanced contrastive learning for text clustering; and we systematically summarize the novelties of our approach and its advantages over existing methods. These revisions make the research contributions more explicit, prominent, and academically persuasive. Thank you again for your thorough review and patient guidance.

*CONCLUSION3.Your future research directions are too generalized, only mentioning “optimizing model performance” without proposing specific and feasible improvement directions. Some potential directions for your reference: optimizing the clustering performance of imbalanced data through weighted mutual information; extending the model to multi language text clustering, combined with cross language embedding techniques; reducing the number of model parameters and improve real-time inference efficiency.*

*Good luck!*

**Response：**

We sincerely thank you for the constructive suggestions on refining our future research directions. Following the your advice, we have revised and detailed our future research directions, providing concrete and actionable plans, including: optimizing clustering performance on imbalanced data via weighted mutual information; extending the model to multilingual text clustering combined with cross-lingual embedding techniques; and reducing the number of model parameters to improve real-time inference efficiency. These revisions make the future research directions more specific, feasible, and academically instructive.

Thank you again for your thorough review and patient guidance.

**Response to reviewers**

*Reviewer #4*

*This manuscript proposes a text clustering approach that integrates contrastive learning, information theory, and a Kolmogorov–Arnold Network (KAN) architecture to address major challenges in text clustering, including the curse of dimensionality and data redundancy. The authors enhance the contrastive learning framework through mutual information maximization strategies, thereby producing more robust text representations while minimizing feature-level redundancy. By increasing the information shared within positive pairs and reducing inter-feature redundancy, the model yields more discriminative representations. Furthermore, by replacing traditional multilayer perceptrons with a KAN architecture, the proposed approach improves its capacity to model complex, high-dimensional data structures. Experimental results on eight datasets demonstrate that the method achieves state-of-the-art performance in terms of accuracy and related evaluation metrics.*
**Response：**

We sincerely appreciate you for the careful reading and positive evaluation of our work. We are grateful for the recognition of our research motivation, framework design, and experimental effectiveness. According to the constructive comments and suggestions provided, we have carefully revised and improved the manuscript to further enhance its clarity, completeness, and academic rigor. We hope that the revised version can meet the requirements of the journal.

*The experimental design and results section are presented in a detailed and informative manner. The findings are comprehensively compared with existing studies in the literature, and the results are compelling. In Figure 2, the iterative performance improvements of the MIKAN model are visually illustrated. Additionally, a thorough ablation study is conducted to demonstrate the contribution of each component of the model. The methodology for determining the λ hyperparameters is also clearly explained.*

**Response：**

We sincerely appreciate you for the positive feedback and careful review of our experimental design and results. We are grateful for your recognition of the detailed and informative presentation, comprehensive comparisons with existing methods, convincing experimental results, clear visualization of the iterative performance improvements in Figure 2, thorough ablation study, and reasonable explanation of the hyperparameter λ selection methodology. We have further refined the manuscript to ensure better readability and academic quality. Thank you again for your valuable comments.

*Points for Improvement:
1.It is recommended that the authors clarify how eliminating correlation redundancy at the feature level differs from traditional dimensionality reduction techniques, and explicitly discuss the conceptual distinctions.*
**Response：**

We sincerely appreciate your constructive suggestion. In the revised manuscript, we have added a dedicated discussion to explicitly explain and compare the essential distinctions between the proposed redundancy elimination mechanism and conventional dimensionality reduction techniques (such as PCA, t-SNE, and feature selection). We have highlighted the differences in motivation, implementation mechanism, and effect on preserving discriminative information for text clustering. These revisions have significantly improved the theoretical clarity and rationality of the proposed method.

*2.The manuscript could be further strengthened by including references to transformer-based pre-trained models applied to text clustering, such as:
i.Yasin Ortakci,Revolutionary text clustering: Investigating transfer learning capacity of SBERT models through pooling techniques,Engineering Science and Technology, an International Journal,Volume 55,2024,101730,ISSN 2215-0986,*[*https://doi.org/10.1016/j.jestch.2024.101730*](https://doi.org/10.1016/j.jestch.2024.101730)*.
ii. Ortakci, Y., Borhan, B. Optimizing SBERT for long text clustering: two novel approaches with empirical insights. J Supercomput 81, 950 (2025).* [*https://doi.org/10.1007/s11227-025-07414-4*](https://doi.org/10.1007/s11227-025-07414-4)
**Response：**

We sincerely appreciate your valuable suggestion and the recommended high-quality references. In the revised version, we have cited the two recommended papers (Ortakci, 2024; Ortakci & Borhan, 2025) in the relevant sections of the literature review and model discussion. We have also supplemented the analysis of the research findings of these studies on SBERT-based text clustering, and compared their methods with our proposed framework to highlight the innovations and advantages of our model. These revisions have enriched the literature support of the manuscript and made the research positioning more clear.

### *3.On page 4, line 163, a reference citation appears to be missing and should be added.* Response：

### We sincerely appreciate your careful check and constructive reminder. In the revised version, we have supplemented the corresponding authoritative reference at this position in strict accordance with the journal’s citation format requirements, and ensured the consistency of the in-text citation and the reference list at the end of the paper. We apologize for this oversight in the original manuscript and have conducted a full check of the entire paper to avoid similar issues.

*4.The expression “integrates contrastive learning enhanced by mutual information and negative samples with KAN (Kolmogorov–Arnold Network)” and its variants are repeated multiple times throughout the manuscript. This recurring formulation, used to describe the proposed model, is somewhat redundant and could be streamlined to improve readability.*

**Response：**

We sincerely appreciate your careful reading and constructive suggestion on improving the readability of the manuscript. In the revised version, we have carefully streamlined and unified the descriptions of the proposed model, using more concise and consistent expressions while retaining the key technical points. We have also checked the full text to eliminate redundant statements and improve the overall fluency and readability of the manuscript.

*5.In Section 3.1, the explanation of the proposed MIKAN model based on Figure 1 includes numbered steps (e.g., (1), (2)); however, the chronological ordering and clarity of these steps are insufficient. Rather than enhancing understanding, the current structure tends to create confusion regarding the processing flow. This section would benefit from being reorganized with clearer sequencing and more explicit explanations of each stage.*
**Response：**

We sincerely appreciate your careful and constructive comment on the organization of Section 3.1. In the revised manuscript, we have completely reorganized and restructured Section 3.1, using a more logical and sequential structure to describe each stage of the model. We have also added more explicit explanations for each module and processing step to ensure the overall pipeline is intuitive and easy to follow. These revisions greatly improve the clarity and readability of the methodology.

*6.Although the experimental results are detailed, the ACC and NMI scores for the Biomedical dataset are noticeably lower than those for the other datasets. It is recommended that the authors further investigate and discuss the compatibility between the proposed method and the characteristics of the datasets, particularly addressing the reasons behind the relatively lower performance on the Biomedical dataset. Such an analysis would further strengthen the manuscript.*

**Response：**

We sincerely appreciate your insightful observation and constructive suggestion. In the revised version, we have supplemented a dedicated discussion section to investigate the compatibility between our proposed method and dataset characteristics, with a focus on the reasons for the relatively lower ACC and NMI scores on the Biomedical dataset:

1. We first analyzed the unique properties of the Biomedical dataset, such as its specialized terminology, complex sentence structures, dense domain knowledge, and higher semantic ambiguity compared to other datasets.
2. We then explored how these characteristics affect the model’s performance: for example, the domain-specific vocabulary may lead to insufficient semantic capture by the pre-trained encoder, and the complex logical relationships between medical concepts may increase the difficulty of mutual information mining in contrastive learning.
3. We also compared the performance of baseline models on the Biomedical dataset to verify whether the performance gap is a common challenge in this domain or specific to our method.

These analyses not only explain the performance difference but also provide valuable insights into the applicability and optimization direction of the proposed method in domain-specific text clustering tasks. Thank you again for your patient and meticulous guidance on our work.
