# Supplementary material for: Contrastive learning with mutual information enhancement and negative sample augmentation combined with KAN for text clustering
Source: PLoS One. 2026 Jul 30;21(7):e0354718. doi: 10.1371/journal.pone.0354718 (PMC13422851; doi:10.1371/journal.pone.0354718)
Supplement: Peer review history

# Peer review: Decision Letter 0

**DOI:** 10.1371/journal.pone.0354718.r001

**Authors:** Ning Cai

22 Feb 2026

PONE-D-25-55666

Contrastive Learning with Mutual Information Enhancement and Negative Sample Augmentation Combined with KAN for Text Clustering

PLOS One

Dear Dr. Xie,

Thank you for submitting your manuscript to PLOS ONE. After careful consideration, we feel that it has merit but does not fully meet PLOS ONE’s publication criteria as it currently stands. Therefore, we invite you to submit a revised version of the manuscript that addresses the points raised during the review process.

Please submit your revised manuscript by Apr 08 2026 11:59PM. If you will need more time than this to complete your revisions, please reply to this message or contact the journal office at plosone@plos.org. When you're ready to submit your revision, log on to https://www.editorialmanager.com/pone/ and select the 'Submissions Needing Revision' folder to locate your manuscript file.

Please include the following items when submitting your revised manuscript:

- A letter that responds to each point raised by the academic editor and reviewer(s). You should upload this letter as a separate file labeled 'Response to Reviewers'.
- A marked-up copy of your manuscript that highlights changes made to the original version. You should upload this as a separate file labeled 'Revised Manuscript with Track Changes'.
- An unmarked version of your revised paper without tracked changes. You should upload this as a separate file labeled 'Manuscript'.

If you would like to make changes to your financial disclosure, please include your updated statement in your cover letter. Guidelines for resubmitting your figure files are available below the reviewer comments at the end of this letter.

If applicable, we recommend that you deposit your laboratory protocols in protocols.io to enhance the reproducibility of your results. Protocols.io assigns your protocol its own identifier (DOI) so that it can be cited independently in the future. For instructions see: https://journals.plos.org/plosone/s/submission-guidelines#loc-laboratory-protocols. Additionally, PLOS ONE offers an option for publishing peer-reviewed Lab Protocol articles, which describe protocols hosted on protocols.io. Read more information on sharing protocols at https://plos.org/protocols?utm_medium=editorial-email&utm_source=authorletters&utm_campaign=protocols.

We look forward to receiving your revised manuscript.

Kind regards,

Ning Cai, Ph.D.

Section Editor

PLOS One

Journal Requirements:

When submitting your revision, we need you to address these additional requirements.

1. Please ensure that your manuscript meets PLOS ONE's style requirements, including those for file naming. The PLOS ONE style templates can be found at

https://journals.plos.org/plosone/s/file?id=wjVg/PLOSOne_formatting_sample_main_body.pdf and

https://journals.plos.org/plosone/s/file?id=ba62/PLOSOne_formatting_sample_title_authors_affiliations.pdf

2. In your Methods section, please include additional information about your dataset and ensure that you have included a statement specifying whether the collection and analysis method complied with the terms and conditions for the source of the data.

3. Thank you for stating the following in the Acknowledgments Section of your manuscript: [This work has been partially supported by Sichuan Science and Technology Program

(Grant No: 2023YFQ0044)]

We note that you have provided funding information that is not currently declared in your Funding Statement. However, funding information should not appear in the Acknowledgments section or other areas of your manuscript. We will only publish funding information present in the Funding Statement section of the online submission form.

Please remove any funding-related text from the manuscript and let us know how you would like to update your Funding Statement. Currently, your Funding Statement reads as follows: "The authors received no specific funding for this work.”

Please include your amended statements within your cover letter; we will change the online submission form on your behalf.

4. We note that there is identifying data in the Supporting Information file <GoogleNews-s, GoogleNews- T and GoogleNews-Ts>. Due to the inclusion of these potentially identifying data, we have removed this file from your file inventory. Prior to sharing human research participant data, authors should consult with an ethics committee to ensure data are shared in accordance with participant consent and all applicable local laws.

Data sharing should never compromise participant privacy. It is therefore not appropriate to publicly share personally identifiable data on human research participants. The following are examples of data that should not be shared:

-Name, initials, physical address

-Ages more specific than whole numbers

-Internet protocol (IP) address

-Specific dates (birth dates, death dates, examination dates, etc.)

-Contact information such as phone number or email address

-Location data

-ID numbers that seem specific (long numbers, include initials, titled “Hospital ID”) rather than random (small numbers in numerical order)

Data that are not directly identifying may also be inappropriate to share, as in combination they can become identifying. For example, data collected from a small group of participants, vulnerable populations, or private groups should not be shared if they involve indirect identifiers (such as sex, ethnicity, location, etc.) that may risk the identification of study participants.

Additional guidance on preparing raw data for publication can be found in our Data Policy (https://journals.plos.org/plosone/s/data-availability#loc-human-research-participant-data-and-other-sensitive-data) and in the following article: http://www.bmj.com/content/340/bmj.c181.long.

Please remove or anonymize all personal information (Name), ensure that the data shared are in accordance with participant consent, and re-upload a fully anonymized data set. Please note that spreadsheet columns with personal information must be removed and not hidden as all hidden columns will appear in the published file.

5. Thank you for stating the following in the Financial Disclosure section: [“The authors received no specific funding for this work.”].

We note that one or more of the authors are employed by a commercial company: name of commercial company.

1. Please provide an amended Funding Statement declaring this commercial affiliation, as well as a statement regarding the Role of Funders in your study. If the funding organization did not play a role in the study design, data collection and analysis, decision to publish, or preparation of the manuscript and only provided financial support in the form of authors' salaries and/or research materials, please review your statements relating to the author contributions, and ensure you have specifically and accurately indicated the role(s) that these authors had in your study. You can update author roles in the Author Contributions section of the online submission form.

Please also include the following statement within your amended Funding Statement.

“The funder provided support in the form of salaries for authors [insert relevant initials], but did not have any additional role in the study design, data collection and analysis, decision to publish, or preparation of the manuscript. The specific roles of these authors are articulated in the ‘author contributions’ section.”

If your commercial affiliation did play a role in your study, please state and explain this role within your updated Funding Statement.

2. Please also provide an updated Competing Interests Statement declaring this commercial affiliation along with any other relevant declarations relating to employment, consultancy, patents, products in development, or marketed products, etc.

Within your Competing Interests Statement, please confirm that this commercial affiliation does not alter your adherence to all PLOS ONE policies on sharing data and materials by including the following statement: ""This does not alter our adherence to  PLOS ONE policies on sharing data and materials.” (as detailed online in our guide for authors http://journals.plos.org/plosone/s/competing-interests) . If this adherence statement is not accurate and  there are restrictions on sharing of data and/or materials, please state these. Please note that we cannot proceed with consideration of your article until this information has been declared.

Please include both an updated Funding Statement and Competing Interests Statement in your cover letter. We will change the online submission form on your behalf.

6. If the reviewer comments include a recommendation to cite specific previously published works, please review and evaluate these publications to determine whether they are relevant and should be cited. There is no requirement to cite these works unless the editor has indicated otherwise.

[Note: HTML markup is below. Please do not edit.]

Reviewers' comments:

Reviewer's Responses to Questions

**Comments to the Author**

1. Is the manuscript technically sound, and do the data support the conclusions?

The manuscript must describe a technically sound piece of scientific research with data that supports the conclusions. Experiments must have been conducted rigorously, with appropriate controls, replication, and sample sizes. The conclusions must be drawn appropriately based on the data presented.

Reviewer #1: Yes

Reviewer #2: Partly

Reviewer #3: Partly

Reviewer #4: Yes

**********

2. Has the statistical analysis been performed appropriately and rigorously?

Reviewer #1: N/A

Reviewer #2: No

Reviewer #3: Yes

Reviewer #4: Yes

**********

3. Have the authors made all data underlying the findings in their manuscript fully available?

The PLOS Data policy requires authors to make all data underlying the findings described in their manuscript fully available without restriction, with rare exception (please refer to the Data Availability Statement in the manuscript PDF file). The data should be provided as part of the manuscript or its supporting information, or deposited to a public repository. For example, in addition to summary statistics, the data points behind means, medians and variance measures should be available. If there are restrictions on publicly sharing data—e.g. participant privacy or use of data from a third party—those must be specified.

Reviewer #1: No

Reviewer #2: Yes

Reviewer #3: Yes

Reviewer #4: Yes

**********

4. Is the manuscript presented in an intelligible fashion and written in standard English?

PLOS ONE does not copyedit accepted manuscripts, so the language in submitted articles must be clear, correct, and unambiguous. Any typographical or grammatical errors should be corrected at revision, so please note any specific errors here.

Reviewer #1: No

Reviewer #2: Yes

Reviewer #3: Yes

Reviewer #4: Yes

**********

5. Review Comments to the Author

Please use the space provided to explain your answers to the questions above. You may also include additional comments for the author, including concerns about dual publication, research ethics, or publication ethics. (Please upload your review as an attachment if it exceeds 20,000 characters)

Reviewer #1: The manuscript presents an experimental evaluation and a technically sophisticated framework. The topic is relevant, and the proposed approach shows potential. However, several issues limit the scientific rigor of the contribution in its current form. Addressing the concerns outlined below is necessary to strengthen the manuscript and improve its credibility.

1. Insufficient Bibliographic Support for Conceptual and Methodological Claims

A major concern throughout the manuscript is the lack of adequate bibliographic support for several conceptual and methodological assertions. This issue is particularly evident in:

• Statements regarding the limitations of contrastive learning in linear embedding spaces;

• General discussions on feature redundancy, anisotropy in embedding spaces, and the curse of dimensionality, which are treated as well-known phenomena but are often not supported by foundational or recent studies;

• Assertions that specific mechanisms, such as mutual information maximization, feature-level redundancy reduction, or KAN-based transformations, effectively or significantly mitigate issues, without references to prior theoretical or empirical work motivating these expectations.

2. Overly Assertive Language and Strength of Claims

The manuscript frequently employs strong and absolute phrasing (e.g., “effectively solves”, “unequivocally outperforms”, “state-of-the-art performance”). In several instances, these claims are not proportionally supported by statistical significance analysis, controlled comparisons, or evaluations across a sufficiently broad range of experimental conditions. Stronger claims should be reinforced with additional quantitative evidence or references to prior studies reporting similar outcomes.

3. Implicit Technical Assumptions and Lack of Definitions

Several technical assumptions are implicitly treated as common knowledge, despite not being universally established. Examples include:

• The relationship between mutual information magnitude and semantic dependency at the feature level;

• The assumed superiority of learnable spline-based activations over linear transformations in high-dimensional clustering contexts;

• The generalization capability of KAN architectures in mitigating the curse of dimensionality.

While these assumptions may be plausible, they should be either supported by references or clearly framed as hypotheses validated empirically in this study. In addition, several technical terms (e.g., training steps, topic popularity, false negative samples) are introduced without definition or are defined only after being used. Definitions should be provided at first occurrence.

4. Limited Engagement with Alternative or Conflicting Viewpoints

Although the Related Work section is extensive, it primarily emphasizes literature that supports the proposed approach. The manuscript would benefit from a more balanced discussion that also considers:

• Alternative paradigms addressing redundancy or anisotropy in embedding spaces;

• Potential drawbacks or trade-offs associated with mutual information–based regularization;

• Known limitations of contrastive learning frameworks that may also apply to the proposed method.

5. Absence of a Dedicated Discussion Section

While the Results section includes localized interpretations of experimental outcomes, the manuscript lacks a dedicated Discussion section. This limits higher-level integration of findings and weakens the connection between empirical results and the broader literature. Although not strictly mandatory, the inclusion of a Discussion section is strongly recommended given the methodological complexity of the proposed framework.

6. Dependence on Hyperparameters and Design Choices

The performance of the proposed method appears sensitive to hyperparameter settings (e.g., λ values, batch size, number of clusters). While hyperparameter tuning is mentioned, several architectural and experimental choices, such as encoder selection, KAN configuration, and loss weighting, are not sufficiently justified through references or systematic empirical analysis. Where possible, ablation studies or references to prior work should be used to justify key design decisions.

7. Interpretation of Visual Analyses

Visualizations based on techniques such as t-SNE and temporal topic popularity trends are informative, but their interpretations are occasionally too strong. Given the qualitative and parameter-sensitive nature of these methods, their illustrative role should be explicitly acknowledged, and they should not be treated as direct quantitative evidence of superiority. In particular, the concept of “topic popularity” requires a clear quantitative definition. The scale used in the corresponding figures (e.g., 0–1000) should be explicitly explained in the text.

Specific Comments

• Consider adding a paragraph at the end of the Introduction outlining the structure of the manuscript.

• Several paragraphs are overly long. Shorter paragraphs (ideally no more than 10–12 lines) would improve readability.

• Figures and tables should be placed closer to their first citation in the text and should be explicitly referenced before appearing.

• Some acronyms and abbreviations are not defined at first occurrence. Please ensure all abbreviations are spelled out in full upon first use.

Abstract

• Lines 13–14: Consider removing the specific days and stating the time frame more generally, for example as “during January and February 2025”.

Introduction

• Lines 18–20: Additional references are required to support the claims made in this passage.

• Lines 25–32: Several key concepts (e.g., contrastive learning, anisotropy, and linear alignment strategies) are introduced without sufficient explanation. Brief contextualization would improve clarity and accessibility for readers less familiar with representation learning.

• Line 33: Please spell out the acronym “SCCL” at its first occurrence in the text. This should be consistently verified for all acronyms throughout the manuscript.

• Line 76: Consider replacing “pitting” with “evaluating”.

• Certain technical terms and claims (e.g., “robust text representations”) could be phrased more precisely or briefly.

• Simplifying long sentences, particularly in the paragraph introducing the proposed method, would improve accessibility.

2. Related Work

• Line 79: Consider replacing “Related work” with “Related Work” for consistency.

• Line 88: Consider using “CNNs”.

• Line 158: Consider using “NLP”.

• Line 161: Please correct the missing space in “generation.For”.

• Line 163: The citation “Pairsupcon” is not numbered and does not appear in the References list.

• Splitting long paragraphs and consolidating overlapping descriptions would improve readability.

3. Proposed Model

• Line 196: Consider replacing “dubbed” with “termed”.

• Line 196: The acronym “MIKAN” is introduced without definition. Please spell out its full meaning at first mention.

• Line 201: Consider replacing “ameliorate” with “mitigate”.

• Lines 207–208: Consider using only “contrastive learning” to avoid redundancy.

• Line 208: Consider replacing “Evidently” with “Consequently” to soften the assertiveness of the statement.

• Line 210: Consider rephrasing as “achieving more robust …” for improved clarity.

• Line 221: Consider replacing “ameliorate” with “mitigate” for consistency.

• The three challenges are currently formulated using “How to …” constructions, which read as implicit questions. Rephrasing these as declarative statements would better suit a methodological section.

• Some statements appear overly assertive (e.g., claims that certain mechanisms “fully capture” specific properties). Softening these formulations would help maintain a cautious scientific tone.

• Certain theoretical statements, such as those regarding the flexibility of KANs due to learnable activation functions, would benefit from supporting references.

• While the overall workflow is clearly presented, briefly clarifying where and how mutual information optimization and feature-level redundancy reduction are implemented within the architecture would further strengthen clarity and reproducibility.

3.1 MIKAN

• Lines 224–226: This passage appears redundant and may be removed, as the same definition is already provided in Lines 194–196.

• Figure 1: Consider using the label “Pre-trained Encoder” for consistency. In addition, the same shade of green is used at two different stages, which may unintentionally suggest that these elements correspond to the same dataset or representation.

3.2 Mutual Information …

• The definition of entropy may need revision. Entropy is typically defined for a random variable rather than a specific realization. Accordingly, the notation should need to be changed from H(x) to H(X) (Formula 1), with the summation taken over the support of the random variable.

3.3 Feature-level Redundancy …

• Line 342 and 380: Consider removing the word “Evidently”.

• Line 369: Please ensure consistent use of quotation marks for ‘easy’.

• The notation appears to be inconsistent throughout the section: batch size is alternately denoted as “b” and “B”, and feature dimensionality as “d” and “D”. Adopting a single notation would improve clarity.

• The normalization of the feature matrix may require clarification. Dividing by the sum of squared feature values does not correspond to standard L2 normalization. If the intention is to project features onto the unit hypersphere, normalization would typically involve the square root of the sum of squared elements, which directly affects feature geometry and similarity computation.

• The probabilistic interpretation of the self-correlation matrix Mself appears to be unclear. While treating a normalized affinity matrix as an approximation of a joint probability distribution can be acceptable in practice, this assumption should be explicitly stated. As currently written, P(Ei, Ej) seems to be treated as a true probability distribution without explicitly satisfying the axioms of probability.

• The definitions of the marginal probability distributions P(Ei) and P(Ej) appear to be ambiguously formatted and do not clearly specify the summation indices. If this interpretation is correct, providing explicit formulations would improve clarity and reproducibility.

• The role of the balancing parameter α is only briefly described. A clearer theoretical interpretation of α (e.g., as smoothing or scaling parameter) would strengthen the proposed loss function.

3.4 Negative Sample ...

• Equations (13–16): Please correct the typo “Achor” to “Anchor” throughout the equations.

• The notation used throughout the section appears to be inconsistent. The total number of samples is denoted interchangeably as “B” and “N”, while additional symbols such as “M”, “L”, and “T” are introduced without clear and unified definitions.

• The loss function associated with hard negative samples (Eq. 14) appears difficult to interpret, as it combines regular negatives, hard negatives, and weighting terms. The interaction between the amplified repulsion term and the standard contrastive denominator may require further clarification.

• While the attraction mechanism proposed for false negative samples is conceptually reasonable, the formulation of the attraction weight and the corresponding loss functions (Eq. 15 and 16) may benefit from clearer theoretical justification. Although an analogy to attention mechanisms is mentioned, this connection does not appear to be formally established.

• The role of the proposed loss LIHSF within the overall optimization framework is not explicitly stated. The authors should clarify whether this loss functions as a primary objective or as an auxiliary regularization term, and how it interacts with the other loss components of the model.

3.5 Kolmogorov–Arnold Networks …

• Lines 420–422: The authors are encouraged to add an appropriate reference.

• Lines 453–454: Please consider adding a reference.

• Line 461: Please ensure consistent use of quotation marks for “curse of dimensionality”.

• While the mathematical formulation of the KAN forward propagation and activation functions appears generally correct, claims regarding model efficiency may require clarification. The assertion that KANs substantially reduce the number of parameters compared to fully connected layers is not sufficiently justified.

• The statement that the proposed approach alleviates the “curse of dimensionality” appears somewhat strong. While KANs can improve function approximation and representation expressiveness in high-dimensional spaces, a more cautious formulation indicating partial mitigation of high-dimensional effects may be more appropriate.

• The computational complexity and scalability of the KAN clustering module are not discussed. Given the use of spline functions and the configuration S = Din, a brief discussion of computational and memory implications would strengthen the section.

• While the clustering loss based on pseudo-labels and cross-view consistency is conceptually appropriate, key operational details remain unclear, such as whether pseudo-labels are updated iteratively, whether gradient flow is stopped during pseudo-label generation, and how training stability is ensured.

• The rationale for producing three separate score matrices (Y’, Y’1, and Y’2) and their precise role within the overall optimization process should be described more explicitly.

4.2 Experimental Dataset

• While the definition of the dataset statistics (N, C, A, and R) is clear, the relevance of the imbalance ratio “R” could be more explicitly discussed.

4.3 Experimental Environment and Parameters

• Line 544: Please ensure consistent quotation formatting for the model name.

• The reported average runtime per dataset (18 h) should be clarified, for example by indicating whether it includes data preprocessing steps.

• When referring to the use of t-SNE for visualization, please clarify whether default parameters were used or specify the key parameter settings adopted.

4.4.1 Comparative Results and Analysis

• Line 569: Consider removing “exemplified by BOW and TF-IDF” due to redundancy.

• Line 570: Consider removing the word “modern”.

• Line 596: Please verify whether the reported value should be “72.69%” instead of “72%”.

• Lines 597–598: The source of the reported values is unclear.

• Line 611: Consider adding “are unavailable in this study” for clarity.

• Line 635: Replace “its” with “MIST” to avoid ambiguity.

• Line 635: The origin of the reported value “9.16%” is unclear.

• Lines 645–646: Please add an appropriate reference.

• Lines 646–648: Please explicitly clarify the stated exception (GoogleNews-TS, SCCL, and NMI).

• Line 672: Replace “chapter” with “section”.

• Lines 675–678: Please add a supporting reference.

• The discussion predominantly emphasizes ACC. A more balanced discussion explicitly referencing NMI values would strengthen the evaluation.

• Reported percentage improvements should clearly specify that they represent absolute percentage-point gains to avoid ambiguity.

• While the critique of traditional methods (e.g., BOW, TF-IDF, GSDPMM) is technically accurate, the tone is occasionally overly strong.

• The discussion would benefit from clearer transitions linking the limitations of existing methods to the motivation and advantages of the proposed approach.

• Terminology referring to the BERT baseline should be standardized (e.g., “BERT-based baseline” or “BERT encoder baseline”).

• Several sentences—particularly in the discussion of MIST—are long and dense. Splitting these sentences would improve readability.

• Explanations for dataset-specific performance degradation (e.g., Biomedical, StackOverflow, Tweet) should be framed more cautiously.

• Claims that KAN “effectively mitigates the curse of dimensionality” should be softened.

• The statement that the results “demonstrate optimal performance across eight datasets” may be interpreted as overly broad, particularly given that the text itself acknowledges cases where the proposed model does not achieve the best results across all metrics.

4.4.2 Ablation Study ...

• Table 5: Please verify the table title. It appears that should be “AgNews, …”.

• Tables 5 and 6: The information presented in these tables is redundant with Figures 3 and 4. The authors are encouraged to consider retaining only one type of visualization (tables or figures).

• The text refers to “changes in clustering metrics across different datasets”, whereas the ablation study is conducted exclusively on the GoogleNews-TS subset.

• The conclusion that the results “conclusively demonstrate the robustness …” is strongly phrased.

4.4.3 Hyperparameter Tuning ...

• Line 712: Consider removing the phrase “which significantly … of 1”, as it is redundant.

• Line 714: Consider removing the phrase “substantially larger … two datasets”.

• The justification for the selected search ranges of λ₁, λ₂, and λ₃ is not explicitly discussed.

• The hyperparameter tuning protocol requires clarification. Why were these values selected?

• The interpretation of the results is largely descriptive and partially repetitive of Figures 5–7. Condensing the narrative and emphasizing insights would improve conciseness.

• The conclusion that λ₁ = 10 and λ₂ = λ₃ = 1×10⁻⁴ are “optimal” should be softened.

4.4.4 Results and Analysis ...

• Figure 8: Please consider increasing the font size of numerical values and textual elements.

• Statements regarding “O(n)” time complexity and the positive correlation between training time and data scale are conceptually correct but not empirically supported.

• The conclusion that the proposed model “necessitates a substantial data scale” to achieve optimal performance should be softened to reflect the experimental scope.

4.4.5 A Case Study ...

• Line 759: Please verify the quotation formatting for “DeepSeek”.

• Lines 763–764: The phrase “a prominent … platform” appears redundant, as the same information is already stated in Line 759.

• Line 764: Replace “chapter” with “subsection”.

• Line 769: Please verify the quotation formatting for “elbow point”.

• Figure 9: Consider translate the terms into English in Figure 9(a).

• Line 779: Replace “The” with “This”.

• Lines 780–782: Please verify the quotation formatting for “DeepSeek”, “Innovation and Development”, and “Robotics”.

• Lines 782–784: The wording is overly strong. Please moderate terms such as “robust”, “superior”, and “strong”.

• Line 796: Consider replacing “curtail” with “reduce”.

• Given the qualitative and parameter-sensitive nature of t-SNE, claims regarding model efficacy or stability should emphasize their illustrative role.

• The criteria used to assign the chosen semantic labels to clusters are not explicitly stated.

• The concept of “topic popularity” is not formally defined. The y-axis scale in Figure 10 (0–1000) should be explained.

• The concluding discussion linking topic popularity trends to broader notions such as “new quality productive forces” is somewhat speculative.

5. Conclusion

• The conclusion does not explicitly state whether the main research objective of the study has been achieved.

• Several performance-related claims are expressed in strong and unqualified terms. While the reported results appear competitive, such statements should be more softened.

• The section does not acknowledge any limitations of the proposed approach.

• The conclusion should include a discussion of future work.

Supplementary Information / Data Availability

• The manuscript lists multiple “Supplementary Files” (Files 1–8). However, the provided links appear to redirect only to publicly available / unvailable dataset repositories rather than to supplementary files prepared by the authors.

• The manuscript lists eight Supplementary Files, including SearchSnippets and Tweet, but these two datasets are not included in the material provided.

Reviewer #2: The manuscript exhibits mixed technical soundness with critical gaps that undermine confidence in the methodology. The most significant technical flaw is the inadequate explanation of the clustering mechanism itself. The paper does not clearly describe how the KAN network transforms embeddings into cluster assignments. Additionally, there is a fundamental conceptual confusion about whether this is truly a clustering method. The approach requires labeled benchmark data for training, yet clustering's primary purpose is to discover structure in unlabeled data. Training on gold-standard labels to learn how to cluster appears contradictory, if labels are available for training, one is performing classification, not unsupervised clustering. The authors do not adequately explain how a method trained on labeled data can generalise to truly unsupervised scenarios like the Weibo dataset, or what advantage this offers over standard supervised classification approaches. I think if this was made clearer then this method has a lot of potential.

On the positive side, the individual components appear theoretically grounded. The mutual information enhancement and contrastive learning components are built on established principles from information theory and self-supervised learning. The ablation studies are properly conducted and demonstrate that each proposed component contributes meaningfully to overall performance, which strengthens confidence in the modular design.

Regarding whether the data support the conclusions, the evidence is mixed. For the benchmark dataset experiments, there is strong quantitative evidence across eight datasets using standard metrics (ACC and NMI) that supports the claims of superior performance compared to existing baselines. However, the Weibo case study presents significant evidential problems. The conclusions about identifying "DeepSeek," "robotics," and "innovation" topic clusters rely solely on word cloud visualisations and manual interpretation, which constitutes insufficient evidence for these claims. To improve upon this, I would like to see a more rigerous approach to verifying the clusters created.

Furthermore, the paper lacks statistical significance testing. Results are presented as point estimates without error bars, confidence intervals, or hypothesis tests. We cannot determine whether the observed improvements over baseline methods represent genuine advances or are within the range of random variance. There is also no mention of multiple experimental runs with different random seeds, which is standard practice for neural network research to account for initialisation sensitivity.

In summary, while the benchmark results provide convincing quantitative support for performance claims, the real-world application conclusions are inadequately supported, and the core methodological description requires substantial clarification before the work can be considered technically sound.

Reviewer #3: PONE-D-25-55666 Comments

Thank you for the opportunity to review the manuscript proposing a text clustering model (MIKAN) that combines mutual information to enhance contrastive learning, negative sample expansion, and KAN network. The research direction has theoretical value and practical application prospects. However, there are many significant issues in the article, including structural integrity, experimental design, literature review, and result analysis. Notably, the expression and support of core novelty, the reproducibility and comprehensiveness of experiments, and the depth of result analysis all need major revision.

ABSTRACT

1.The expression of the core novelty point is not precise enough. It merely mentions “combining mutual information enhanced contrastive learning with KAN network” without explicitly explaining the collaborative mechanism among mutual information enhancement, negative sample expansion, and KAN. Meanwhile, it is unclear how this collaborative mechanism specifically addresses the three core issues of text clustering (insufficient representation robustness, redundancy, and dimensionality disaster). You need to supplement the expression with “maximizing mutual information to explore the nonlinear relationship between positive samples, expanding negative samples to optimize sample discrimination, and adapting the KAN network to the complex structure of high-dimensional data, which work together to solve the three core problems of text clustering”, highlighting the innovative logic.

2.The description of the experimental part is overly concise, merely stating that “it performs better on eight datasets” without mentioning the representative characteristics of the datasets (such as data size, domain diversity, category distribution differences, etc.), nor providing specific improvement margins for key performance indicators, thus lacking persuasiveness.

INTRODUCTION

1.The research background is not sufficiently systematic, and the development trajectory of text clustering is not clearly outlined. It merely lists the limitations of existing methods without explaining the technological evolution logic behind the mainstream adoption of contrastive learning + deep learning. Furthermore, it fails to identify the common root causes of current research gaps, such as neglecting feature-level redundancy and inadequate modeling of nonlinear relationships. You need to refine the research problem.

2.The discussion on related work such as SCCL, RSTC, and MIST merely states “proposed XXX method” without delving into a comparison of the core flaws and potential improvements of each method, which results in the necessity and entry point of this study not being sufficiently prominent. I suggest that you should strengthen literature comparative discussion and commentary to highlight the necessity of the research.

3.The correspondence between research objectives and novelty is not clear. You merely vaguely mentions “solving three major problems” without explicitly explaining how each novelty point precisely corresponds to a research problem, resulting in a broken logical chain.

RELATED WORK

1.The classification system is unreasonable, as it confuses traditional methods, deep learning methods, and contrastive learning methods without establishing a clear classification framework based on technological evolution or method types, leading to a chaotic logic in the literature review. Therefore, please reconstruct the classification framework. Provide a comprehensive review categorized as traditional text clustering methods → deep learning text clustering methods → contrastive learning text clustering methods → high-dimensional data processing methods. For each category, elaborate on the core ideas, representative methods, and limitations, thereby enhancing logical clarity.

2.The overview of positive and negative sample augmentation methods in contrastive learning is not comprehensive enough, omitting important recent research such as the mixed negative sample strategy of MixCSE and the soft negative sample method of SNCSE. Simultaneously, you fail to discuss the applicable scenarios and limitations of different augmentation strategies. Thus, you can add comments on the latest research such as MixCSE constructs difficult negative samples by mixing positive and negative sample features, balancing discrimination and computational efficiency; SNCSE treats the negation form of anchor samples as soft negative samples to alleviate the problem of feature suppression. In addition, you can analyze limitations such as token-level expansion easily destroys semantics, and supervised negative samples rely on labeled data.

3.The review of related research on KAN network is absent, with only a brief mention of KAN in the introduction, and no comprehensive analysis of KAN’s application status in high-dimensional data processing and clustering tasks in the related work section. This results in a lack of literature support for the rationality of introducing this technology.

4.Now, it is 2026 and your references is outdated, as the literature from the past three years are completely insufficient.

PROPOSED MODEL

1.The description of the model architecture diagram (Figure 1) is not detailed enough, as it fails to clearly indicate the input and output of each module, data flow, and core parameters. It prevents readers from intuitively understand the model’s operational process. I suggest that refine the description of the model architecture diagram: the original text can be divided into two branches after preprocessing. One branch is subjected to data augmentation and encoder generation to produce M₁⁺ and M₂⁺, which are then output as M₁⁺' and M₂⁺' after passing through the mutual information enhancement module, and subsequently optimized by the IHSF module. The other branch is encoded by the encoder to generate M, which is output as M' after feature redundancy reduction. Finally, the three inputs are fed into the KAN network to obtain clustering results." In addition, label key parameters such as the encoder output dimension D=768 should be highlighted.

2.The derivation and explanation of key formulas are insufficient, such as the absence of the derivation process for mutual information loss functions (Loss_MII, Loss_MIF), the lack of clarity in the physical significance of hyperparameters like α and β, and inconsistent symbol definitions in the formulas (e.g., some symbols are not explained in the main text).

3.Please clarify the module collaboration mechanism. Supplementing the statement like: The outputs M₁⁺' and M₂⁺' of the mutual information enhancement module are used as inputs to the IHSF module, and the optimized features are fused with M' and input into the KAN network; the total loss function balances clustering loss, contrastive loss, and mutual information loss through λ₁, λ₂, and λ₃, achieving end-to-end collaborative optimization.

4.Please supplement KAN configuration details. You need to clarify parameters such as KAN network hidden layer dimension S=768 (consistent with input dimension), using 3rd order B-spline function, learning rate set to 1e-5, adopting L2 regularization (weight decay coefficient 1e-4), and activation function is x·sigmoid(x) combined with spline function, to ensure reproducibility.

5.The Explanation of the basis for weight setting is required. Thus, please supplementing the statement as follows: The optimal values of λ₁ ∈ {0, 1, ..., 100}, λ₂ ∈ {1e-5, ..., 0.1}, and λ₃ ∈ {1e-5, ..., 0.1} were determined through grid search, and the final settings were λ₁=10, λ₂=1e-4, and λ₃=1e-4. This setting achieved the optimal balance between ACC and NMI on the validation set.

EXPERIMENTS

1.The comprehensiveness of the experimental design is insufficient, as no control variable experiments such as comparisons of different encoders and different data augmentation strategies beyond ablation experiments were set up. This makes it impossible to verify the independent contributions of each component and the robustness of the model.

2.The description of the dataset is not detailed enough. You only provides basic information such as the number of samples and categories, without specifying the data preprocessing steps (such as text cleaning, word segmentation, stop word removal, etc.). Furthermore, it does not provide key features such as the degree of imbalance in category distribution and text length distribution of the dataset, which limits the reproducibility of the experiment.

3.The selection and setting of baseline models are not reasonable enough. Some baseline models (such as STC²-LPI, Self-Train) have not been tested on all datasets, and the parameter configurations of the baseline models (such as encoder type, training steps, etc.) have not been specified, leading to unfair comparison.

4.The analysis of experimental results is superficial, merely comparing ACC and NMI values without analyzing the performance differences and reasons of the model on different types of datasets (such as balanced/unbalanced, short/long texts), nor does it analyze the rationality of clustering results through visualization (such as intra-cluster compactness, inter-cluster separability).

5.Supplementary efficiency analysis should be added. Add a model efficiency comparison table to compare the parameter quantity (such as MIKAN parameter quantity of 12M and SCCL parameter quantity of 10M), training time (such as MIKAN training for 18 hours and RSTC training for 16 hours on the AgNews dataset), and inference speed (such as the number of samples processed per second) of the model and the baseline model, and analyze the advantages and disadvantages of the model’s efficiency.

6.The description of the collection and preprocessing methods for the case data is not detailed enough, and the crawling strategy for Weibo data (such as the specific scope, frequency, and deduplication method of crawling keywords) is not explained. The parameter configuration and embedding dimensions of the Chinese text embedding model (bge base zh-v1.5) are also not explained, which affects the reproducibility of the case.

7.The basis for determining the number of clusters is insufficient. It merely relies on the elbow method and silhouette coefficient to set k=3, without verifying the clustering results for k=2 and k=4, nor does it incorporate domain knowledge to justify the reasonableness of k=3 (such as whether it aligns with the actual classification logic of scientific topics).

8.There is a problem of insufficient analysis and interpretation of the case results. Your results only displays clustering results through word cloud and T-SNE visualization, without in-depth analysis of the temporal evolution rules of each topic (such as the peak popularity of DeepSeek topics and event correlation), user participation characteristics, etc., nor demonstrating the actual application value of the case results.

CONCLUSION

1.The connection between research conclusions and novelty is not close enough, only summarizing the advantages of the model in a general way, without corresponding to the research questions and novelty points raised in the introduction one by one, resulting in an incomplete research logic loop.

2.The explanation of research contributions is not clear enough, without distinguishing between theoretical contributions and practical application contributions, and without highlighting the breakthrough of the research (such as whether KAN has been combined with mutual information enhanced contrastive learning for text clustering for the first time).

3.Your future research directions are too generalized, only mentioning “optimizing model performance” without proposing specific and feasible improvement directions. Some potential directions for your reference: optimizing the clustering performance of imbalanced data through weighted mutual information; extending the model to multi language text clustering, combined with cross language embedding techniques; reducing the number of model parameters and improve real-time inference efficiency.

Good luck!

Reviewer #4: This manuscript proposes a text clustering approach that integrates contrastive learning, information theory, and a Kolmogorov–Arnold Network (KAN) architecture to address major challenges in text clustering, including the curse of dimensionality and data redundancy. The authors enhance the contrastive learning framework through mutual information maximization strategies, thereby producing more robust text representations while minimizing feature-level redundancy. By increasing the information shared within positive pairs and reducing inter-feature redundancy, the model yields more discriminative representations. Furthermore, by replacing traditional multilayer perceptrons with a KAN architecture, the proposed approach improves its capacity to model complex, high-dimensional data structures. Experimental results on eight datasets demonstrate that the method achieves state-of-the-art performance in terms of accuracy and related evaluation metrics.

The experimental design and results section are presented in a detailed and informative manner. The findings are comprehensively compared with existing studies in the literature, and the results are compelling. In Figure 2, the iterative performance improvements of the MIKAN model are visually illustrated. Additionally, a thorough ablation study is conducted to demonstrate the contribution of each component of the model. The methodology for determining the λ hyperparameters is also clearly explained.

Points for Improvement:

1.It is recommended that the authors clarify how eliminating correlation redundancy at the feature level differs from traditional dimensionality reduction techniques, and explicitly discuss the conceptual distinctions.

2.The manuscript could be further strengthened by including references to transformer-based pre-trained models applied to text clustering, such as:

i.Yasin Ortakci,Revolutionary text clustering: Investigating transfer learning capacity of SBERT models through pooling techniques,Engineering Science and Technology, an International Journal,Volume 55,2024,101730,ISSN 2215-0986,https://doi.org/10.1016/j.jestch.2024.101730.

ii. Ortakci, Y., Borhan, B. Optimizing SBERT for long text clustering: two novel approaches with empirical insights. J Supercomput 81, 950 (2025). https://doi.org/10.1007/s11227-025-07414-4

3.On page 4, line 163, a reference citation appears to be missing and should be added.

4.The expression “integrates contrastive learning enhanced by mutual information and negative samples with KAN (Kolmogorov–Arnold Network)” and its variants are repeated multiple times throughout the manuscript. This recurring formulation, used to describe the proposed model, is somewhat redundant and could be streamlined to improve readability.

5.In Section 3.1, the explanation of the proposed MIKAN model based on Figure 1 includes numbered steps (e.g., (1), (2)); however, the chronological ordering and clarity of these steps are insufficient. Rather than enhancing understanding, the current structure tends to create confusion regarding the processing flow. This section would benefit from being reorganized with clearer sequencing and more explicit explanations of each stage.

6.Although the experimental results are detailed, the ACC and NMI scores for the Biomedical dataset are noticeably lower than those for the other datasets. It is recommended that the authors further investigate and discuss the compatibility between the proposed method and the characteristics of the datasets, particularly addressing the reasons behind the relatively lower performance on the Biomedical dataset. Such an analysis would further strengthen the manuscript.

**********

6. PLOS authors have the option to publish the peer review history of their article (what does this mean?). If published, this will include your full peer review and any attached files.

If you choose “no”, your identity will remain anonymous but your review may still be made public.

**Do you want your identity to be public for this peer review?**  For information about this choice, including consent withdrawal, please see our Privacy Policy.

Reviewer #1: **Yes:** Ismael Weber

Reviewer #2: No

Reviewer #3: **Yes:** Zeyu Li

Reviewer #4: No

**********

[NOTE: If reviewer comments were submitted as an attachment file, they will be attached to this email and accessible via the submission site. Please log into your account, locate the manuscript record, and check for the action link "View Attachments". If this link does not appear, there are no attachment files.]

To ensure your figures meet our technical requirements, please review our figure guidelines: https://journals.plos.org/plosone/s/figures

You may also use PLOS’s free figure tool, NAAS, to help you prepare publication quality figures: https://journals.plos.org/plosone/s/figures#loc-tools-for-figure-preparation.

NAAS will assess whether your figures meet our technical requirements by comparing each figure against our figure specifications.

# Author comment: Author response to Decision Letter 1

**DOI:** 10.1371/journal.pone.0354718.r002

29 Apr 2026

Dear Editor and Reviewers,

We are grateful for the insightful feedback and valuable suggestions provided by you and the esteemed reviewers on our manuscript (No.: PONE-D-25-55666; Title: Contrastive Learning with Mutual Information Enhancement and Negative Sample Augmentation Combined with KAN for Text Clustering; Authors: Yuanmin Zhang, Hao Li,Chunzhi Xie,Yong Huang, Zhenyi Wu,Yanjun Li) submitted to PLOS One. Following a comprehensive review of the comments received, we have diligently made all the recommended revisions to our manuscript. All modifications in the manuscript are marked with red font for reviewer-related revisions and blue font for improvements in organization and language, to ensure clarity and traceability.

Please do not hesitate to contact us should you have any further questions or require additional details regarding our revised submission. We kindly request acknowledgment of this revision's receipt and look forward to your response.

Sincerely,

Chunzhi Xie

On behalf of all co-authors

mail: xcz_xihua@sina.com

Address: School of Computer and Software Engineering, Xihua University, Chengdu 610039, China

Response to reviewers

Reviewer #1:

The manuscript presents an experimental evaluation and a technically sophisticated framework. The topic is relevant, and the proposed approach shows potential. However, several issues limit the scientific rigor of the contribution in its current form. Addressing the concerns outlined below is necessary to strengthen the manuscript and improve its credibility.

Response�

We sincerely appreciate you for the thorough, professional, and insightful evaluation of our manuscript. We are grateful for your recognition of the relevance of our topic, the technical soundness of the proposed framework, and the potential of our experimental evaluation. We fully acknowledge that the scientific rigor and credibility of the manuscript need to be further strengthened. In response to all your constructive comments and suggestions, we have carefully revised the manuscript point-by-point, supplemented sufficient literature support, adjusted overly assertive expressions, clarified technical definitions and assumptions, added a dedicated Discussion section, improved the analysis of experimental results and visualizations, and polished the overall presentation. We believe these revisions have greatly improved the academic quality, rigor, and readability of the manuscript. Thank you again for your valuable and constructive comments.

1. Insufficient Bibliographic Support for Conceptual and Methodological Claims

A major concern throughout the manuscript is the lack of adequate bibliographic support for several conceptual and methodological assertions. This issue is particularly evident in:

• Statements regarding the limitations of contrastive learning in linear embedding spaces;

• General discussions on feature redundancy, anisotropy in embedding spaces, and the curse of dimensionality, which are treated as well-known phenomena but are often not supported by foundational or recent studies;

• Assertions that specific mechanisms, such as mutual information maximization, feature-level redundancy reduction, or KAN-based transformations, effectively or significantly mitigate issues, without references to prior theoretical or empirical work motivating these expectations.

Response�

We sincerely appreciate your valuable and constructive comment. In the revised manuscript, we have comprehensively addressed this issue by supplementing authoritative, foundational, and recent references for all relevant assertions, specifically:

• For statements regarding the limitations of contrastive learning in linear embedding spaces, we have cited classic and cutting-edge studies that systematically discuss the constraints of contrastive learning when applied to linear embedding scenarios, clarifying the theoretical basis for our related claims.

• For general discussions on feature redundancy, embedding space anisotropy, and the curse of dimensionality, we have added foundational works that define and validate these phenomena, as well as recent studies that extend related research, ensuring these concepts are supported by sufficient academic evidence rather than being treated as self-evident.

• For assertions about the effectiveness of specific mechanisms (mutual information maximization, feature-level redundancy reduction, KAN-based transformations), we have referenced prior theoretical studies that establish the rationality of these mechanisms and empirical works that verify their effectiveness in similar tasks, clearly linking our approach to existing academic achievements.

All added references are carefully selected to be representative and relevant, and we have ensured consistent formatting between in-text citations and the reference list. These revisions significantly strengthen the academic foundation of the manuscript and enhance the persuasiveness of our conceptual and methodological claims. Thank you again for your patient and meticulous guidance on our work.

2. Overly Assertive Language and Strength of Claims

The manuscript frequently employs strong and absolute phrasing (e.g., “effectively solves”, “unequivocally outperforms”, “state-of-the-art performance”). In several instances, these claims are not proportionally supported by statistical significance analysis, controlled comparisons, or evaluations across a sufficiently broad range of experimental conditions. Stronger claims should be reinforced with additional quantitative evidence or references to prior studies reporting similar outcomes.

Response�

We sincerely appreciate your insightful and constructive comment. In the revised manuscript, we have comprehensively revised the language throughout the text to address this issue:

We have replaced all strong and absolute phrases (e.g., “effectively solves”, “unequivocally outperforms”, “state-of-the-art performance”) with modest, academically appropriate expressions (e.g., “can effectively alleviate”, “achieves competitive performance compared with”, “demonstrates promising performance relative to existing methods”), ensuring our claims are prudent and consistent with academic norms.

For claims that lacked sufficient support, we have supplemented additional quantitative evidence, including statistical significance analysis (e.g., t-tests) to verify the reliability of experimental results, expanded controlled comparisons with baseline methods, and extended evaluations across a broader range of experimental conditions to enhance the persuasiveness of our conclusions.

For stronger claims regarding the effectiveness of our proposed method, we have referenced prior studies that report similar outcomes, establishing a clear link between our findings and existing academic research to further reinforce the rationality of our assertions.

We have carefully checked the entire manuscript to ensure no overly assertive language remains, and all claims are now proportionally supported by evidence or relevant references. These revisions significantly improve the scientific rigor and credibility of the manuscript. Thank you again for your patient and meticulous guidance on our work.

3. Implicit Technical Assumptions and Lack of Definitions

Several technical assumptions are implicitly treated as common knowledge, despite not being universally established. Examples include:

• The relationship between mutual information magnitude and semantic dependency at the feature level;

• The assumed superiority of learnable spline-based activations over linear transformations in high-dimensional clustering contexts;

• The generalization capability of KAN architectures in mitigating the curse of dimensionality.

While these assumptions may be plausible, they should be either supported by references or clearly framed as hypotheses validated empirically in this study. In addition, several technical terms (e.g., training steps, topic popularity, false negative samples) are introduced without definition or are defined only after being used. Definitions should be provided at first occurrence.

Response�

We sincerely appreciate your careful and constructive comment. In the revised manuscript, we have made thorough revisions as follows:

• We have explicitly stated and justified all previously implicit technical assumptions. For the relationship between mutual information and feature-level semantic dependency, the superiority of learnable spline-based activations over linear transformations in high-dimensional clustering, and the generalization ability of KAN in alleviating the curse of dimensionality, we have either added supporting references or clearly described them as hypotheses that are verified through empirical experiments in this work.

• We have checked all technical terms and provided clear definitions at their first occurrence, including training steps, topic popularity, false negative samples, and other key expressions.

These changes ensure that all assumptions are well-supported and all terminologies are clearly explained, which greatly improves the logical consistency and readability of the manuscript. Thank you again for your patient and meticulous guidance on our work.

4. Limited Engagement with Alternative or Conflicting Viewpoints

Although the Related Work section is extensive, it primarily emphasizes literature that supports the proposed approach. The manuscript would benefit from a more balanced discussion that also considers:

• Alternative paradigms addressing redundancy or anisotropy in embedding spaces;

• Potential drawbacks or trade-offs associated with mutual information–based regularization;

• Known limitations of contrastive learning frameworks that may also apply to the proposed method.

Response�

We sincerely appreciate your professional and constructive comment. In the revised manuscript, we have significantly expanded and improved the Related Work section to provide a more impartial review:

We have added a systematic discussion of alternative paradigms for addressing embedding space redundancy and anisotropy, comparing their ideas, strengths, and limitations with our method.

We have explicitly analyzed the potential drawbacks and trade-offs associated with mutual information–based regularization, providing a more comprehensive view of its mechanism.

We have also supplemented a discussion on the inherent limitations of contrastive learning frameworks and have objectively explained which challenges may also apply to our proposed model.

These revisions make the literature review more comprehensive, balanced, and persuasive, and better highlight the positioning and contributions of this work. Thank you again for your patient and meticulous guidance on our work.

5. Absence of a Dedicated Discussion Section

While the Results section includes localized interpretations of experimental outcomes, the manuscript lacks a dedicated Discussion section. This limits higher-level integration of findings and weakens the connection between empirical results and the broader literature. Although not strictly mandatory, the inclusion of a Discussion section is strongly recommended given the methodological complexity of the proposed framework.

Response�

We sincerely appreciate your valuable and professional suggestion. In the revised manuscript, we have added a new, independent Discussion section. In this section, we systematically summarize and interpret the key experimental findings, analyze the effectiveness and rationality of the proposed framework, discuss the insights obtained from the experiments, compare our results with relevant studies in the literature, and objectively address the limitations and potential future improvements of the proposed method. This addition greatly enhances the logical completeness, interpretability, and academic rigor of the manuscript. Thank you again for your patient and meticulous guidance on our work.

6. Dependence on Hyperparameters and Design Choices

The performance of the proposed method appears sensitive to hyperparameter settings (e.g., λ values, batch size, number of clusters). While hyperparameter tuning is mentioned, several architectural and experimental choices, such as encoder selection, KAN configuration, and loss weighting, are not sufficiently justified through references or systematic empirical analysis. Where possible, ablation studies or references to prior work should be used to justify key design decisions.

Response�

We sincerely appreciate your insightful and constructive comment. In the revised manuscript, we have comprehensively supplemented the justification and analysis for all critical hyperparameters and architectural decisions:

• We have added systematic sensitivity analysis for important hyperparameters including λ, batch size, and number of clusters, clearly demonstrating their influence on model performance and the rationale behind our final selection.

• We have supplemented detailed justification for encoder selection, KAN structure configuration, and loss function weighting strategies, supported by relevant references and additional ablation experiments to verify the rationality and effectiveness of these design choices.

• We have clearly explained the motivation and experimental evidence for each key design decision to ensure transparency and sufficiency of justification.

These revisions significantly strengthen the reliability and persuasiveness of the experimental design and model architecture. Thank you again for your patient and meticulous guidance on our work.

7. Interpretation of Visual Analyses

Visualizations based on techniques such as t-SNE and temporal topic popularity trends are informative, but their interpretations are occasionally too strong. Given the qualitative and parameter-sensitive nature of these methods, their illustrative role should be explicitly acknowledged, and they should not be treated as direct quantitative evidence of superiority. In particular, the concept of “topic popularity” requires a clear quantitative definition. The scale used in the corresponding figures (e.g., 0–1000) should be explicitly explained in the text.

Specific Comments

• Consider adding a paragraph at the end of the Introduction outlining the structure of the manuscript.

• Several paragraphs are overly long. Shorter paragraphs (ideally no more than 10–12 lines) would improve readability.

• Figures and tables should be placed closer to their first citation in the text and should be explicitly referenced before appearing.

• Some acronyms and abbreviations are not defined at first occurrence. Please ensure all abbreviations are spelled out in full upon first use.

Response�

We sincerely appreciate your careful and detailed comments. In the revised manuscript, we have addressed all points as follows:

• We have revised the interpretation of t-SNE and temporal topic popularity visualizations. We explicitly acknowledge that these figures serve a qualitative and illustrative purpose rather than providing direct quantitative evidence of performance superiority. We have also added a clear quantitative definition of “topic popularity” and explicitly explained the scale range (e.g., 0–1000) in the text and figure captions.

• We have added a paragraph at the end of the Introduction to clearly outline the overall structure of the manuscript.

• We have split all overly long paragraphs into shorter ones (generally within 10–12 lines) to improve readability.

• We have adjusted the positions of all figures and tables to ensure they appear immediately after their first citation in the text and are properly referenced before they appear.

• We have checked all acronyms and abbreviations carefully and ensured that each abbreviation is defined in full at its first occurrence throughout the manuscript.

We believe these revisions have greatly improved the standardization, clarity, and readability of the manuscript. Thank you again for your patient and meticulous guidance on our work.

Abstract

• Lines 13–14: Consider removing the specific days and stating the time frame more generally, for example as “during January and February 2025”.

Response�

We thank you for the suggestion. We have revised the text to rep

Attachment Submitted filename: response letter (major revison).docx

# Peer review: Decision Letter 1

**DOI:** 10.1371/journal.pone.0354718.r003

**Authors:** Ning Cai

13 Jul 2026

Contrastive Learning with Mutual Information Enhancement and Negative Sample Augmentation Combined with KAN for Text Clustering

PONE-D-25-55666R1

Dear Dr. Xie,

We’re pleased to inform you that your manuscript has been judged scientifically suitable for publication and will be formally accepted for publication once it meets all outstanding technical requirements.

Within one week, you’ll receive an e-mail detailing the required amendments. When these have been addressed, you’ll receive a formal acceptance letter and your manuscript will be scheduled for publication.

An invoice will be generated when your article is formally accepted. Please note, if your institution has a publishing partnership with PLOS and your article meets the relevant criteria, all or part of your publication costs will be covered. Please make sure your user information is up-to-date by logging into Editorial Manager at Editorial Manager® and clicking the ‘Update My Information' link at the top of the page. For questions related to billing, please contact billing support.

If your institution or institutions have a press office, please notify them about your upcoming paper to help maximize its impact. If they’ll be preparing press materials, please inform our press team as soon as possible -- no later than 48 hours after receiving the formal acceptance. Your manuscript will remain under strict press embargo until 2 pm Eastern Time on the date of publication. For more information, please contact onepress@plos.org.

Kind regards,

Ning Cai, Ph.D.

Section Editor

PLOS One

Additional Editor Comments (optional):

The sole reviewer’s minor revision requests are purely editorial in nature. I see no scientific or technical barriers, and I am confident the authors can resolve them during final production. Accordingly, I approve the manuscript for publication as is.

Reviewers' comments:

Reviewer's Responses to Questions

**Comments to the Author**

1. If the authors have adequately addressed your comments raised in a previous round of review and you feel that this manuscript is now acceptable for publication, you may indicate that here to bypass the “Comments to the Author” section, enter your conflict of interest statement in the “Confidential to Editor” section, and submit your "Accept" recommendation.

Reviewer #1: All comments have been addressed

Reviewer #3: All comments have been addressed

Reviewer #4: All comments have been addressed

**********

2. Is the manuscript technically sound, and do the data support the conclusions?

The manuscript must describe a technically sound piece of scientific research with data that supports the conclusions. Experiments must have been conducted rigorously, with appropriate controls, replication, and sample sizes. The conclusions must be drawn appropriately based on the data presented.

Reviewer #1: Yes

Reviewer #3: Yes

Reviewer #4: Yes

**********

3. Has the statistical analysis been performed appropriately and rigorously?

Reviewer #1: Yes

Reviewer #3: Yes

Reviewer #4: Yes

**********

4. Have the authors made all data underlying the findings in their manuscript fully available?

The PLOS Data policy requires authors to make all data underlying the findings described in their manuscript fully available without restriction, with rare exception (please refer to the Data Availability Statement in the manuscript PDF file). The data should be provided as part of the manuscript or its supporting information, or deposited to a public repository. For example, in addition to summary statistics, the data points behind means, medians and variance measures should be available. If there are restrictions on publicly sharing data—e.g. participant privacy or use of data from a third party—those must be specified.

Reviewer #1: Yes

Reviewer #3: Yes

Reviewer #4: Yes

**********

5. Is the manuscript presented in an intelligible fashion and written in standard English?

PLOS ONE does not copyedit accepted manuscripts, so the language in submitted articles must be clear, correct, and unambiguous. Any typographical or grammatical errors should be corrected at revision, so please note any specific errors here.

Reviewer #1: Yes

Reviewer #3: Yes

Reviewer #4: Yes

**********

6. Review Comments to the Author

Please use the space provided to explain your answers to the questions above. You may also include additional comments for the author, including concerns about dual publication, research ethics, or publication ethics. (Please upload your review as an attachment if it exceeds 20,000 characters)

Reviewer #1: I appreciate the considerable effort made by the authors in revising the manuscript. After examining the revised version and the detailed response letter, I am satisfied that the authors have adequately addressed the concerns raised in my previous review. The manuscript has improved substantially in terms of scientific rigor, methodological clarity, bibliographic support, discussion of results, and overall presentation.

The authors have strengthened the theoretical foundation of the study, improved the justification of methodological choices, incorporated additional analyses, clarified several technical aspects, and added a dedicated Discussion section. Overall, the revised manuscript presents a stronger and more convincing contribution than the original submission.

I have only a few minor comments before publication:

1. Although readability has improved, some sections still contain relatively long and dense paragraphs. A final editorial revision aimed at improving readability would be beneficial.

2. The Abstract could be further strengthened by:

* adding a brief introductory sentence to better contextualize the research problem;

* highlighting one or two key quantitative results obtained by the proposed method;

* explicitly stating the main outcome of the Weibo case study validation rather than only mentioning that the validation was performed.

3. There appears to be an incomplete placeholder in line 983 (“peak at k = 3 (0.XX), indicating…”), which should be corrected before publication.

These issues are primarily editorial in nature and do not affect my overall positive assessment of the manuscript. Therefore, I recommend Minor Revision.

Reviewer #3: Thank you for your detailed response. I think that you have addressed all of the concerns. Good luck.

Reviewer #4: I believe that the newly added sections in the discussion have made the article clearer, more coherent, and more compelling. Additionally, the revision of Section 3.1 has successfully resolved the previously existing inconsistencies.

**********

7. PLOS authors have the option to publish the peer review history of their article (what does this mean?). If published, this will include your full peer review and any attached files.

If you choose “no”, your identity will remain anonymous but your review may still be made public.

**Do you want your identity to be public for this peer review?**  For information about this choice, including consent withdrawal, please see our Privacy Policy.

Reviewer #1: **Yes:** Ismael Weber

Reviewer #3: **Yes:** Zeyu Li

Reviewer #4: No

**********

# Editor report: Acceptance letter

**DOI:** 10.1371/journal.pone.0354718.r004

**Authors:** Ning Cai

PONE-D-25-55666R1

PLOS One

Dear Dr. Xie,

I'm pleased to inform you that your manuscript has been deemed suitable for publication in PLOS One. Congratulations! Your manuscript is now being handed over to our production team.

At this stage, our production department will prepare your paper for publication. This includes ensuring the following:

* All references, tables, and figures are properly cited

* All relevant supporting information is included in the manuscript submission,

* There are no issues that prevent the paper from being properly typeset

You will receive further instructions from the production team, including instructions on how to review your proof when it is ready. Please keep in mind that we are working through a large volume of accepted articles, so please give us a few days to review your paper and let you know the next and final steps.

Lastly, if your institution or institutions have a press office, please let them know about your upcoming paper now to help maximize its impact. If they'll be preparing press materials, please inform our press team within the next 48 hours. Your manuscript will remain under strict press embargo until 2 pm Eastern Time on the date of publication. For more information, please contact onepress@plos.org.

You will receive an invoice from PLOS for your publication fee after your manuscript has reached the completed accept phase. If you receive an email requesting payment before acceptance or for any other service, this may be a phishing scheme. Learn how to identify phishing emails and protect your accounts at https://explore.plos.org/phishing.

If we can help with anything else, please email us at customercare@plos.org.

Thank you for submitting your work to PLOS ONE and supporting open access.

Kind regards,

PLOS ONE Editorial Office Staff

on behalf of

Dr. Ning Cai

Section Editor

PLOS One
